# Supplementary material for: GTP Binding Protein Gtr1 Cooperating with ASF1 Regulates Asexual Development in Stemphylium eturmiunum
Source: Int J Mol Sci. 2022 Jul 28;23(15):8355. doi: 10.3390/ijms23158355 (PMC9369126; doi:10.3390/ijms23158355)
Supplement: Supplementary file 1 [file ijms-23-08355-s001.zip › Supplementary figure S1.pdf]

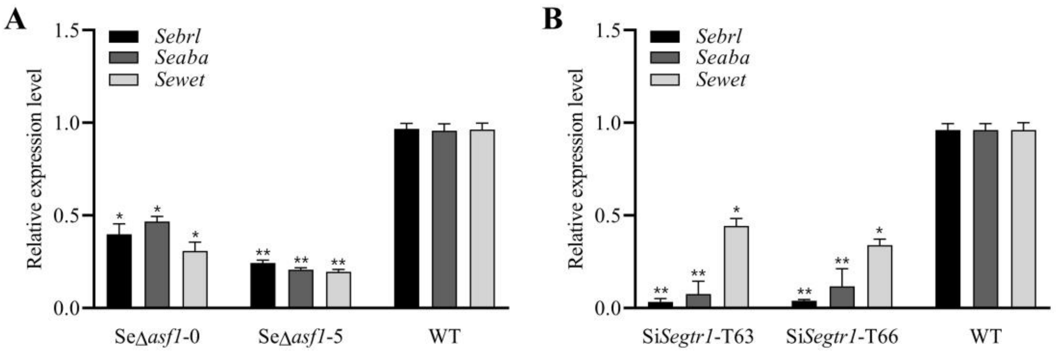

Figure S1. The expression patterns of *Sebrl*, *Seaba*, and *Sewet* in knockout mutants or silenced strains. (A) The expression levels of *Sebrl*, *Seaba*, and *Sewet* in two SeΔ*asf1* mutants were measured by qRT-PCR. (B) The expression levels of *Sebrl*, *Seaba*, and *Sewet* in two Si*Segrt1* lines were measured by qRT-PCR. The degree of WT was assigned to value 1.0. Two *Seasf1* deleted mutants were SeΔ*asf1*-0 and SeΔ*asf1*-5, and two *Segtr1*-silenced lines were Si*Segtr1*-T63 and Si*Segtr1*-T66. The *Actin* in *S. eturmiunum* was used as endogenous control. The bars indicated statistically significant differences (ANOVA; **P* < 0.05, ***P* < 0.01).
