# Supplementary material for: GTP Binding Protein Gtr1 Cooperating with ASF1 Regulates Asexual Development in Stemphylium eturmiunum
Source: Int J Mol Sci. 2022 Jul 28;23(15):8355. doi: 10.3390/ijms23158355 (PMC9369126; doi:10.3390/ijms23158355)
Supplement: Supplementary file 1 [file ijms-23-08355-s001.zip › Supplementary table S1.pdf]

Supplementary Table S1. The differentially expressed genes in SeΔ*asf1* vs WT-sexual

Gene	SeΔ*asf1*	WT-sexual	Log2 (FoldChange)	P-value	Q-value
TR2615-c0_g1	93	0	7.539158811	6.24273E-22	2.48E-20
TR17574-c0_g1	90	0	7.491853096	2.38181E-21	9.14084E-20
TR27799-c0_g1	88	0	7.459431619	5.84558E-21	2.21342E-19
TR16437-c0_g1	86	0	7.426264755	1.44073E-20	5.3133E-19
TR9510-c0_g1	85	0	7.409390936	2.26548E-20	8.28305E-19
TR13441-c0_g1	75	0	7.22881869	2.22861E-18	7.27242E-17
TR4098-c0_g1	74	0	7.209453366	3.54952E-18	1.14946E-16
TR9687-c0_g1	69	0	7.108524457	3.70826E-17	1.14628E-15
TR10557-c0_g1	62	0	6.95419631	1.04847E-15	2.95612E-14
TR26388-c0_g1	122	1	6.930737338	2.08421E-29	1.16945E-27
TR13980-c0_g1	59	0	6.882643049	4.48857E-15	1.23282E-13
TR14126-c0_g1	58	0	6.857980995	7.31087E-15	1.9698E-13
TR7604-c0_g1	58	0	6.857980995	7.31087E-15	1.9698E-13
TR26318-c0_g2	55	0	6.781359714	3.18965E-14	8.16691E-13
TR813-c0_g2	54	0	6.754887502	5.22923E-14	1.31905E-12
TR27245-c0_g1	52	0	6.700439718	1.41272E-13	3.47083E-12
TR11027-c0_g1	50	0	6.64385619	3.84362E-13	9.12663E-12
TR17076-c0_g1	50	0	6.64385619	3.84362E-13	9.12663E-12
TR813-c0_g1	50	0	6.64385619	3.84362E-13	9.12663E-12
TR9834-c0_g1	50	0	6.64385619	3.84362E-13	9.12663E-12
TR25819-c0_g1	49	0	6.614709844	6.35721E-13	1.4745E-11
TR26318-c0_g1	49	0	6.614709844	6.35721E-13	1.4745E-11
TR14753-c0_g1	48	0	6.584962501	1.05342E-12	2.41695E-11
TR26425-c0_g1	47	0	6.554588852	1.74889E-12	3.94344E-11
TR27245-c0_g3	47	0	6.554588852	1.74889E-12	3.94344E-11
TR12587-c0_g1	45	0	6.491853096	4.84872E-12	1.05957E-10
TR13220-c0_g1	45	0	6.491853096	4.84872E-12	1.05957E-10
TR17332-c0_g1	45	0	6.491853096	4.84872E-12	1.05957E-10
TR22378-c0_g1	45	0	6.491853096	4.84872E-12	1.05957E-10
TR13861-c0_g1	44	0	6.459431619	8.09774E-12	1.73829E-10
TR15544-c0_g1	44	0	6.459431619	8.09774E-12	1.73829E-10
TR19676-c0_g1	43	0	6.426264755	1.35517E-11	2.8235E-10
TR27218-c0_g1	43	0	6.426264755	1.35517E-11	2.8235E-10
TR5122-c0_g1	43	0	6.426264755	1.35517E-11	2.8235E-10
TR9819-c0_g1	43	0	6.426264755	1.35517E-11	2.8235E-10
TR10020-c0_g1	168	2	6.392317423	8.48464E-41	6.67825E-39
TR8142-c0_g1	42	0	6.392317423	2.27268E-11	4.64965E-10
TR18132-c0_g1	41	0	6.357552005	3.81957E-11	7.59506E-10
TR372-c0_g1	41	0	6.357552005	3.81957E-11	7.59506E-10
TR8717-c0_g1	41	0	6.357552005	3.81957E-11	7.59506E-10

TR14944-c0_g1	40	0	6.321928095	6.43346E-11	1.24718E-09
TR17193-c0_g1	40	0	6.321928095	6.43346E-11	1.24718E-09
TR21155-c0_g1	39	0	6.285402219	1.08605E-10	2.06305E-09
TR23461-c0_g1	39	0	6.285402219	1.08605E-10	2.06305E-09
TR25912-c0_g2	39	0	6.285402219	1.08605E-10	2.06305E-09
TR27102-c0_g1	39	0	6.285402219	1.08605E-10	2.06305E-09
TR14949-c0_g1	228	3	6.247927513	5.83119E-55	6.43753E-53
TR14794-c0_g1	38	0	6.247927513	1.8376E-10	3.40694E-09
TR10026-c0_g1	224	3	6.222392421	4.62632E-54	4.91585E-52
TR23050-c0_g1	37	0	6.209453366	3.11654E-10	5.68512E-09
TR8382-c0_g1	37	0	6.209453366	3.11654E-10	5.68512E-09
TR3245-c0_g1	72	1	6.169925001	1.60161E-18	5.24652E-17
TR1301-c0_g1	36	0	6.169925001	5.29834E-10	9.40279E-09
TR25390-c0_g1	36	0	6.169925001	5.29834E-10	9.40279E-09
TR4665-c0_g1	36	0	6.169925001	5.29834E-10	9.40279E-09
TR5346-c0_g1	36	0	6.169925001	5.29834E-10	9.40279E-09
TR8249-c0_g2	36	0	6.169925001	5.29834E-10	9.40279E-09
TR9834-c0_g2	36	0	6.169925001	5.29834E-10	9.40279E-09
TR14883-c0_g1	35	0	6.129283017	9.02978E-10	1.56972E-08
TR17253-c0_g2	35	0	6.129283017	9.02978E-10	1.56972E-08
TR23153-c0_g1	35	0	6.129283017	9.02978E-10	1.56972E-08
TR7617-c0_g1	69	1	6.108524457	7.79182E-18	2.48539E-16
TR25116-c0_g1	34	0	6.087462841	1.54281E-09	2.58676E-08
TR25116-c0_g2	34	0	6.087462841	1.54281E-09	2.58676E-08
TR26144-c0_g1	34	0	6.087462841	1.54281E-09	2.58676E-08
TR12604-c0_g1	33	0	6.044394119	2.64285E-09	4.30382E-08
TR7953-c0_g1	33	0	6.044394119	2.64285E-09	4.30382E-08
TR18393-c0_g1	32	0	6	4.53928E-09	7.21251E-08
TR260-c0_g1	32	0	6	4.53928E-09	7.21251E-08
TR5517-c0_g1	32	0	6	4.53928E-09	7.21251E-08
TR9858-c0_g1	32	0	6	4.53928E-09	7.21251E-08
TR9257-c0_g1	62	1	5.95419631	3.25867E-16	9.53557E-15
TR4070-c0_g1	31	0	5.95419631	7.81788E-09	1.20831E-07
TR7982-c0_g1	31	0	5.95419631	7.81788E-09	1.20831E-07
TR26836-c0_g1	123	2	5.942514505	1.33808E-30	7.89899E-29
TR6696-c0_g1	60	1	5.906890596	9.57637E-16	2.71352E-14
TR24769-c0_g1	30	0	5.906890596	1.35024E-08	2.01898E-07
TR6443-c0_g1	30	0	5.906890596	1.35024E-08	2.01898E-07
TR22515-c0_g1	29	0	5.857980995	2.33875E-08	3.41596E-07
TR11944-c0_g1	28	0	5.807354922	4.063E-08	5.7708E-07
TR22162-c0_g1	28	0	5.807354922	4.063E-08	5.7708E-07
TR3982-c0_g1	28	0	5.807354922	4.063E-08	5.7708E-07
TR18804-c0_g1	166	3	5.790076931	9.94186E-41	7.78916E-39
TR27199-c0_g1	165	3	5.781359714	1.70781E-40	1.33188E-38

TR17228-c0_g2	27	0	5.754887502	7.0801E-08	9.62969E-07
TR26574-c0_g1	27	0	5.754887502	7.0801E-08	9.62969E-07
TR27102-c0_g2	27	0	5.754887502	7.0801E-08	9.62969E-07
TR14759-c1_g1	53	1	5.727920455	4.34578E-14	1.10605E-12
TR15887-c0_g1	26	0	5.700439718	1.23766E-07	1.64261E-06
TR26530-c0_g1	26	0	5.700439718	1.23766E-07	1.64261E-06
TR11027-c0_g2	25	0	5.64385619	2.17058E-07	2.78933E-06
TR15603-c0_g1	25	0	5.64385619	2.17058E-07	2.78933E-06
TR24172-c0_g1	25	0	5.64385619	2.17058E-07	2.78933E-06
TR4922-c0_g2	25	0	5.64385619	2.17058E-07	2.78933E-06
TR7513-c0_g1	25	0	5.64385619	2.17058E-07	2.78933E-06
TR9819-c0_g2	25	0	5.64385619	2.17058E-07	2.78933E-06
TR26147-c0_g1	49	1	5.614709844	3.96599E-13	9.40405E-12
TR22378-c0_g2	24	0	5.584962501	3.81953E-07	4.73648E-06
TR8143-c0_g1	24	0	5.584962501	3.81953E-07	4.73648E-06
TR8765-c0_g2	24	0	5.584962501	3.81953E-07	4.73648E-06
TR15995-c0_g1	46	1	5.523561956	2.11549E-12	4.75742E-11
TR13570-c0_g1	23	0	5.523561956	6.74455E-07	8.07509E-06
TR21948-c0_g1	23	0	5.523561956	6.74455E-07	8.07509E-06
TR26215-c0_g1	23	0	5.523561956	6.74455E-07	8.07509E-06
TR320-c0_g1	23	0	5.523561956	6.74455E-07	8.07509E-06
TR717-c0_g1	23	0	5.523561956	6.74455E-07	8.07509E-06
TR2944-c0_g1	45	1	5.491853096	3.70779E-12	8.17605E-11
TR16877-c0_g1	44	1	5.459431619	6.50895E-12	1.4079E-10
TR12524-c0_g1	22	0	5.459431619	1.19524E-06	1.36472E-05
TR13134-c0_g1	22	0	5.459431619	1.19524E-06	1.36472E-05
TR14559-c0_g1	22	0	5.459431619	1.19524E-06	1.36472E-05
TR17253-c0_g1	22	0	5.459431619	1.19524E-06	1.36472E-05
TR22955-c0_g1	22	0	5.459431619	1.19524E-06	1.36472E-05
TR25733-c0_g1	22	0	5.459431619	1.19524E-06	1.36472E-05
TR26963-c0_g1	22	0	5.459431619	1.19524E-06	1.36472E-05
TR346-c0_g1	22	0	5.459431619	1.19524E-06	1.36472E-05
TR10581-c0_g2	42	1	5.392317423	2.01565E-11	4.13373E-10
TR11504-c0_g1	42	1	5.392317423	2.01565E-11	4.13373E-10
TR18440-c0_g1	42	1	5.392317423	2.01565E-11	4.13373E-10
TR23950-c0_g1	42	1	5.392317423	2.01565E-11	4.13373E-10
TR14746-c0_g1	21	0	5.392317423	2.12606E-06	2.32449E-05
TR17127-c0_g2	21	0	5.392317423	2.12606E-06	2.32449E-05
TR21420-c0_g1	21	0	5.392317423	2.12606E-06	2.32449E-05
TR25912-c0_g1	21	0	5.392317423	2.12606E-06	2.32449E-05
TR27739-c0_g1	21	0	5.392317423	2.12606E-06	2.32449E-05
TR5243-c0_g1	21	0	5.392317423	2.12606E-06	2.32449E-05
TR7237-c0_g1	21	0	5.392317423	2.12606E-06	2.32449E-05
TR9063-c0_g2	21	0	5.392317423	2.12606E-06	2.32449E-05

TR9714-c0_g1	21	0	5.392317423	2.12606E-06	2.32449E-05
TR10581-c0_g1	41	1	5.357552005	3.55583E-11	7.1206E-10
TR3404-c0_g1	361	9	5.325930025	7.07253E-86	1.29293E-83
TR11504-c0_g2	40	1	5.321928095	6.28346E-11	1.22088E-09
TR24263-c0_g1	40	1	5.321928095	6.28346E-11	1.22088E-09
TR10887-c0_g1	20	0	5.321928095	3.79642E-06	3.9842E-05
TR13759-c0_g1	20	0	5.321928095	3.79642E-06	3.9842E-05
TR19042-c0_g1	20	0	5.321928095	3.79642E-06	3.9842E-05
TR24172-c0_g3	20	0	5.321928095	3.79642E-06	3.9842E-05
TR3542-c0_g1	20	0	5.321928095	3.79642E-06	3.9842E-05
TR5346-c0_g2	20	0	5.321928095	3.79642E-06	3.9842E-05
TR6213-c0_g1	588	15	5.292781749	1.622E-138	4.5206E-136
TR18751-c0_g1	39	1	5.285402219	1.11224E-10	2.11044E-09
TR4153-c0_g1	76	2	5.247927513	2.26989E-19	7.87575E-18
TR3395-c0_g1	38	1	5.247927513	1.97218E-10	3.6485E-09
TR10131-c0_g1	19	0	5.247927513	6.80635E-06	6.87155E-05
TR10283-c1_g1	19	0	5.247927513	6.80635E-06	6.87155E-05
TR15576-c0_g1	19	0	5.247927513	6.80635E-06	6.87155E-05
TR22265-c0_g1	19	0	5.247927513	6.80635E-06	6.87155E-05
TR24689-c0_g1	19	0	5.247927513	6.80635E-06	6.87155E-05
TR26730-c0_g7	19	0	5.247927513	6.80635E-06	6.87155E-05
TR4922-c0_g1	19	0	5.247927513	6.80635E-06	6.87155E-05
TR22788-c0_g1	373	10	5.221103725	2.327E-88	4.49569E-86
TR966-c0_g1	371	10	5.213347282	7.17482E-88	1.34045E-85
TR3425-c0_g1	37	1	5.209453366	3.50315E-10	6.37668E-09
TR21419-c0_g1	36	1	5.169925001	6.23365E-10	1.09824E-08
TR23361-c0_g5	18	0	5.169925001	1.22537E-05	0.000117967
TR25390-c0_g2	18	0	5.169925001	1.22537E-05	0.000117967
TR26215-c0_g2	18	0	5.169925001	1.22537E-05	0.000117967
TR26923-c0_g1	18	0	5.169925001	1.22537E-05	0.000117967
TR27753-c0_g1	18	0	5.169925001	1.22537E-05	0.000117967
TR3963-c0_g1	18	0	5.169925001	1.22537E-05	0.000117967
TR5517-c0_g2	18	0	5.169925001	1.22537E-05	0.000117967
TR8765-c0_g1	18	0	5.169925001	1.22537E-05	0.000117967
TR9063-c0_g1	18	0	5.169925001	1.22537E-05	0.000117967
TR24531-c0_g1	70	2	5.129283017	6.92045E-18	2.21576E-16
TR20312-c0_g1	139	4	5.118941073	6.57389E-34	4.36581E-32
TR15870-c0_g1	34	1	5.087462841	1.98459E-09	3.28536E-08
TR26957-c0_g1	34	1	5.087462841	1.98459E-09	3.28536E-08
TR14910-c0_g1	17	0	5.087462841	2.21569E-05	0.000203399
TR15563-c0_g1	17	0	5.087462841	2.21569E-05	0.000203399
TR15578-c0_g1	17	0	5.087462841	2.21569E-05	0.000203399
TR26215-c0_g3	17	0	5.087462841	2.21569E-05	0.000203399
TR5415-c0_g1	17	0	5.087462841	2.21569E-05	0.000203399

TR8717-c0_g2	17	0	5.087462841	2.21569E-05	0.000203399
TR8765-c0_g3	17	0	5.087462841	2.21569E-05	0.000203399
TR9146-c0_g1	17	0	5.087462841	2.21569E-05	0.000203399
TR18454-c0_g1	67	2	5.06608919	3.86279E-17	1.19188E-15
TR19907-c0_g1	33	1	5.044394119	3.55089E-09	5.70604E-08
TR21047-c0_g1	33	1	5.044394119	3.55089E-09	5.70604E-08
TR3016-c0_g1	33	1	5.044394119	3.55089E-09	5.70604E-08
TR8070-c0_g1	33	1	5.044394119	3.55089E-09	5.70604E-08
TR13662-c0_g1	161	5	5.008988783	8.42398E-39	6.22691E-37
TR18723-c0_g1	32	1	5	6.36532E-09	9.92834E-08
TR10096-c0_g1	16	0	5	4.02458E-05	0.000348564
TR10323-c1_g1	16	0	5	4.02458E-05	0.000348564
TR1144-c0_g1	16	0	5	4.02458E-05	0.000348564
TR12096-c0_g1	16	0	5	4.02458E-05	0.000348564
TR15901-c0_g1	16	0	5	4.02458E-05	0.000348564
TR1723-c0_g1	16	0	5	4.02458E-05	0.000348564
TR18574-c0_g1	16	0	5	4.02458E-05	0.000348564
TR20766-c0_g2	16	0	5	4.02458E-05	0.000348564
TR22373-c0_g1	16	0	5	4.02458E-05	0.000348564
TR24873-c0_g1	16	0	5	4.02458E-05	0.000348564
TR26992-c0_g1	16	0	5	4.02458E-05	0.000348564
TR4367-c0_g1	16	0	5	4.02458E-05	0.000348564
TR5776-c0_g1	16	0	5	4.02458E-05	0.000348564
TR6064-c0_g1	16	0	5	4.02458E-05	0.000348564
TR9776-c0_g1	16	0	5	4.02458E-05	0.000348564
TR5331-c0_g1	63	2	4.977279923	3.86851E-16	1.12813E-14
TR12818-c0_g1	157	5	4.972692654	8.31159E-38	6.09087E-36
TR10021-c0_g1	125	4	4.965784285	1.95631E-30	1.14295E-28
TR11143-c0_g1	31	1	4.95419631	1.14322E-08	1.72921E-07
TR3458-c0_g1	31	1	4.95419631	1.14322E-08	1.72921E-07
TR10323-c1_g2	15	0	4.906890596	7.34503E-05	0.000596633
TR10486-c0_g1	15	0	4.906890596	7.34503E-05	0.000596633
TR1701-c0_g1	15	0	4.906890596	7.34503E-05	0.000596633
TR1723-c0_g2	15	0	4.906890596	7.34503E-05	0.000596633
TR19390-c0_g1	15	0	4.906890596	7.34503E-05	0.000596633
TR22688-c0_g1	15	0	4.906890596	7.34503E-05	0.000596633
TR23323-c0_g1	15	0	4.906890596	7.34503E-05	0.000596633
TR23527-c0_g3	15	0	4.906890596	7.34503E-05	0.000596633
TR24994-c0_g1	15	0	4.906890596	7.34503E-05	0.000596633
TR25859-c0_g1	15	0	4.906890596	7.34503E-05	0.000596633
TR4266-c0_g1	15	0	4.906890596	7.34503E-05	0.000596633
TR6528-c0_g1	15	0	4.906890596	7.34503E-05	0.000596633
TR6664-c0_g1	15	0	4.906890596	7.34503E-05	0.000596633
TR8264-c0_g4	15	0	4.906890596	7.34503E-05	0.000596633

TR971-c0_g1	15	0	4.906890596	7.34503E-05	0.000596633
TR5850-c0_g1	29	1	4.857980995	3.70916E-08	5.30814E-07
TR26349-c0_g1	114	4	4.832890014	1.10427E-27	5.83047E-26
TR18233-c0_g1	85	3	4.824428435	4.84027E-21	1.84924E-19
TR26282-c0_g3	28	1	4.807354922	6.7009E-08	9.15789E-07
TR11766-c0_g2	14	0	4.807354922	0.000134718	0.001031243
TR13279-c0_g1	14	0	4.807354922	0.000134718	0.001031243
TR14077-c0_g1	14	0	4.807354922	0.000134718	0.001031243
TR15167-c0_g1	14	0	4.807354922	0.000134718	0.001031243
TR15230-c0_g3	14	0	4.807354922	0.000134718	0.001031243
TR17569-c0_g1	14	0	4.807354922	0.000134718	0.001031243
TR18877-c0_g1	14	0	4.807354922	0.000134718	0.001031243
TR20561-c0_g1	14	0	4.807354922	0.000134718	0.001031243
TR20991-c0_g1	14	0	4.807354922	0.000134718	0.001031243
TR23265-c0_g3	14	0	4.807354922	0.000134718	0.001031243
TR24661-c0_g1	14	0	4.807354922	0.000134718	0.001031243
TR26699-c0_g1	14	0	4.807354922	0.000134718	0.001031243
TR26730-c0_g5	14	0	4.807354922	0.000134718	0.001031243
TR2688-c0_g1	14	0	4.807354922	0.000134718	0.001031243
TR27393-c0_g1	14	0	4.807354922	0.000134718	0.001031243
TR3914-c0_g1	14	0	4.807354922	0.000134718	0.001031243
TR4364-c0_g1	14	0	4.807354922	0.000134718	0.001031243
TR5760-c0_g1	14	0	4.807354922	0.000134718	0.001031243
TR5850-c0_g2	14	0	4.807354922	0.000134718	0.001031243
TR6091-c0_g1	14	0	4.807354922	0.000134718	0.001031243
TR8264-c0_g5	14	0	4.807354922	0.000134718	0.001031243
TR8446-c0_g1	14	0	4.807354922	0.000134718	0.001031243
TR8829-c0_g1	14	0	4.807354922	0.000134718	0.001031243
TR8958-c0_g1	14	0	4.807354922	0.000134718	0.001031243
TR7721-c0_g1	53	2	4.727920455	1.30086E-13	3.21459E-12
TR11887-c0_g2	26	1	4.700439718	2.20022E-07	2.81677E-06
TR18262-c0_g1	26	1	4.700439718	2.20022E-07	2.81677E-06
TR8119-c0_g1	26	1	4.700439718	2.20022E-07	2.81677E-06
TR12338-c0_g4	13	0	4.700439718	0.000248387	0.001784075
TR13705-c0_g1	13	0	4.700439718	0.000248387	0.001784075
TR15791-c0_g1	13	0	4.700439718	0.000248387	0.001784075
TR17055-c0_g1	13	0	4.700439718	0.000248387	0.001784075
TR17235-c0_g1	13	0	4.700439718	0.000248387	0.001784075
TR18372-c0_g1	13	0	4.700439718	0.000248387	0.001784075
TR19202-c0_g1	13	0	4.700439718	0.000248387	0.001784075
TR20028-c0_g1	13	0	4.700439718	0.000248387	0.001784075
TR20237-c0_g2	13	0	4.700439718	0.000248387	0.001784075
TR22130-c0_g1	13	0	4.700439718	0.000248387	0.001784075
TR22808-c0_g1	13	0	4.700439718	0.000248387	0.001784075

TR22870-c0_g1	13	0	4.700439718	0.000248387	0.001784075
TR23457-c0_g1	13	0	4.700439718	0.000248387	0.001784075
TR23894-c0_g1	13	0	4.700439718	0.000248387	0.001784075
TR25192-c0_g1	13	0	4.700439718	0.000248387	0.001784075
TR25209-c0_g1	13	0	4.700439718	0.000248387	0.001784075
TR25422-c0_g1	13	0	4.700439718	0.000248387	0.001784075
TR26730-c0_g4	13	0	4.700439718	0.000248387	0.001784075
TR26730-c0_g8	13	0	4.700439718	0.000248387	0.001784075
TR2720-c0_g1	13	0	4.700439718	0.000248387	0.001784075
TR27220-c0_g1	13	0	4.700439718	0.000248387	0.001784075
TR27653-c0_g1	13	0	4.700439718	0.000248387	0.001784075
TR27883-c0_g2	13	0	4.700439718	0.000248387	0.001784075
TR2852-c0_g1	13	0	4.700439718	0.000248387	0.001784075
TR3616-c0_g1	13	0	4.700439718	0.000248387	0.001784075
TR4622-c0_g1	13	0	4.700439718	0.000248387	0.001784075
TR5958-c0_g1	13	0	4.700439718	0.000248387	0.001784075
TR6643-c0_g1	13	0	4.700439718	0.000248387	0.001784075
TR7905-c0_g1	13	0	4.700439718	0.000248387	0.001784075
TR8181-c0_g10	13	0	4.700439718	0.000248387	0.001784075
TR8993-c0_g1	13	0	4.700439718	0.000248387	0.001784075
TR9146-c0_g2	13	0	4.700439718	0.000248387	0.001784075
TR9833-c0_g1	13	0	4.700439718	0.000248387	0.001784075
TR1006-c0_g1	76	3	4.662965013	9.12359E-19	3.02955E-17
TR17566-c0_g1	76	3	4.662965013	9.12359E-19	3.02955E-17
TR3456-c0_g1	76	3	4.662965013	9.12359E-19	3.02955E-17
TR14134-c0_g1	25	1	4.64385619	3.99911E-07	4.94114E-06
TR14722-c0_g1	25	1	4.64385619	3.99911E-07	4.94114E-06
TR20625-c0_g1	25	1	4.64385619	3.99911E-07	4.94114E-06
TR8006-c0_g2	49	2	4.614709844	1.36442E-12	3.11369E-11
TR611-c0_g1	96	4	4.584962501	3.86556E-23	1.61473E-21
TR13063-c0_g1	24	1	4.584962501	7.28378E-07	8.62181E-06
TR15987-c0_g1	24	1	4.584962501	7.28378E-07	8.62181E-06
TR1864-c0_g1	24	1	4.584962501	7.28378E-07	8.62181E-06
TR14562-c0_g1	12	0	4.584962501	0.000460489	0.003071373
TR15244-c0_g1	12	0	4.584962501	0.000460489	0.003071373
TR17079-c0_g1	12	0	4.584962501	0.000460489	0.003071373
TR17127-c0_g3	12	0	4.584962501	0.000460489	0.003071373
TR17276-c0_g1	12	0	4.584962501	0.000460489	0.003071373
TR17555-c0_g1	12	0	4.584962501	0.000460489	0.003071373
TR18614-c0_g2	12	0	4.584962501	0.000460489	0.003071373
TR198-c0_g1	12	0	4.584962501	0.000460489	0.003071373
TR21628-c0_g1	12	0	4.584962501	0.000460489	0.003071373
TR22712-c0_g1	12	0	4.584962501	0.000460489	0.003071373
TR23257-c0_g1	12	0	4.584962501	0.000460489	0.003071373

TR23264-c0_g1	12	0	4.584962501	0.000460489	0.003071373
TR23312-c0_g1	12	0	4.584962501	0.000460489	0.003071373
TR24642-c0_g1	12	0	4.584962501	0.000460489	0.003071373
TR24756-c0_g1	12	0	4.584962501	0.000460489	0.003071373
TR25097-c0_g1	12	0	4.584962501	0.000460489	0.003071373
TR25421-c0_g2	12	0	4.584962501	0.000460489	0.003071373
TR25786-c0_g1	12	0	4.584962501	0.000460489	0.003071373
TR26317-c0_g2	12	0	4.584962501	0.000460489	0.003071373
TR26733-c0_g1	12	0	4.584962501	0.000460489	0.003071373
TR26951-c0_g1	12	0	4.584962501	0.000460489	0.003071373
TR27178-c0_g1	12	0	4.584962501	0.000460489	0.003071373
TR27718-c0_g1	12	0	4.584962501	0.000460489	0.003071373
TR3239-c0_g2	12	0	4.584962501	0.000460489	0.003071373
TR3496-c0_g1	12	0	4.584962501	0.000460489	0.003071373
TR5752-c0_g1	12	0	4.584962501	0.000460489	0.003071373
TR6592-c0_g1	12	0	4.584962501	0.000460489	0.003071373
TR717-c0_g2	12	0	4.584962501	0.000460489	0.003071373
TR7436-c0_g1	12	0	4.584962501	0.000460489	0.003071373
TR750-c0_g1	12	0	4.584962501	0.000460489	0.003071373
TR8185-c1_g1	12	0	4.584962501	0.000460489	0.003071373
TR8603-c0_g1	12	0	4.584962501	0.000460489	0.003071373
TR9938-c0_g1	12	0	4.584962501	0.000460489	0.003071373
TR22022-c0_g1	238	10	4.572889668	8.34159E-55	9.09091E-53
TR12586-c0_g1	71	3	4.564784619	1.70521E-17	5.34886E-16
TR13243-c0_g1	328	14	4.550197083	1.10553E-74	1.67817E-72
TR10509-c0_g1	117	5	4.548436625	9.3599E-28	4.95734E-26
TR12159-c0_g1	69	3	4.523561956	5.51924E-17	1.67277E-15
TR14803-c0_g1	23	1	4.523561956	1.3294E-06	1.49778E-05
TR25536-c0_g1	23	1	4.523561956	1.3294E-06	1.49778E-05
TR7245-c0_g1	23	1	4.523561956	1.3294E-06	1.49778E-05
TR8889-c0_g1	23	1	4.523561956	1.3294E-06	1.49778E-05
TR22976-c0_g1	316	14	4.496425826	1.18645E-71	1.72404E-69
TR20368-c0_g1	67	3	4.48112669	1.78973E-16	5.31954E-15
TR15012-c0_g1	156	7	4.478047297	3.3062E-36	2.36175E-34
TR18838-c0_g1	89	4	4.475733431	2.33077E-21	8.96521E-20
TR2704-c0_g2	66	3	4.459431619	3.22506E-16	9.45349E-15
TR2742-c0_g1	22	1	4.459431619	2.43145E-06	2.63634E-05
TR3863-c0_g1	22	1	4.459431619	2.43145E-06	2.63634E-05
TR10131-c0_g2	11	0	4.459431619	0.000858686	0.005274132
TR10171-c0_g1	11	0	4.459431619	0.000858686	0.005274132
TR1065-c0_g1	11	0	4.459431619	0.000858686	0.005274132
TR10872-c0_g1	11	0	4.459431619	0.000858686	0.005274132
TR10990-c0_g1	11	0	4.459431619	0.000858686	0.005274132
TR11766-c0_g1	11	0	4.459431619	0.000858686	0.005274132

TR12679-c0_g1	11	0	4.459431619	0.000858686	0.005274132
TR14077-c0_g2	11	0	4.459431619	0.000858686	0.005274132
TR14720-c0_g2	11	0	4.459431619	0.000858686	0.005274132
TR15981-c0_g1	11	0	4.459431619	0.000858686	0.005274132
TR17347-c0_g1	11	0	4.459431619	0.000858686	0.005274132
TR17421-c0_g1	11	0	4.459431619	0.000858686	0.005274132
TR17747-c0_g1	11	0	4.459431619	0.000858686	0.005274132
TR18018-c0_g1	11	0	4.459431619	0.000858686	0.005274132
TR20049-c0_g1	11	0	4.459431619	0.000858686	0.005274132
TR21087-c0_g1	11	0	4.459431619	0.000858686	0.005274132
TR21087-c0_g2	11	0	4.459431619	0.000858686	0.005274132
TR21200-c0_g1	11	0	4.459431619	0.000858686	0.005274132
TR21428-c0_g1	11	0	4.459431619	0.000858686	0.005274132
TR21813-c0_g1	11	0	4.459431619	0.000858686	0.005274132
TR21957-c0_g1	11	0	4.459431619	0.000858686	0.005274132
TR23024-c0_g1	11	0	4.459431619	0.000858686	0.005274132
TR23110-c0_g3	11	0	4.459431619	0.000858686	0.005274132
TR23500-c1_g1	11	0	4.459431619	0.000858686	0.005274132
TR24507-c0_g1	11	0	4.459431619	0.000858686	0.005274132
TR25097-c0_g2	11	0	4.459431619	0.000858686	0.005274132
TR26283-c0_g2	11	0	4.459431619	0.000858686	0.005274132
TR27021-c0_g1	11	0	4.459431619	0.000858686	0.005274132
TR2765-c0_g3	11	0	4.459431619	0.000858686	0.005274132
TR2893-c0_g1	11	0	4.459431619	0.000858686	0.005274132
TR3511-c0_g1	11	0	4.459431619	0.000858686	0.005274132
TR3854-c1_g1	11	0	4.459431619	0.000858686	0.005274132
TR3921-c0_g1	11	0	4.459431619	0.000858686	0.005274132
TR3982-c2_g1	11	0	4.459431619	0.000858686	0.005274132
TR4541-c0_g1	11	0	4.459431619	0.000858686	0.005274132
TR4728-c0_g1	11	0	4.459431619	0.000858686	0.005274132
TR4745-c0_g1	11	0	4.459431619	0.000858686	0.005274132
TR5104-c0_g1	11	0	4.459431619	0.000858686	0.005274132
TR527-c0_g1	11	0	4.459431619	0.000858686	0.005274132
TR5415-c0_g2	11	0	4.459431619	0.000858686	0.005274132
TR5835-c0_g1	11	0	4.459431619	0.000858686	0.005274132
TR5961-c0_g1	11	0	4.459431619	0.000858686	0.005274132
TR6529-c0_g1	11	0	4.459431619	0.000858686	0.005274132
TR7356-c0_g2	11	0	4.459431619	0.000858686	0.005274132
TR753-c0_g2	11	0	4.459431619	0.000858686	0.005274132
TR8181-c0_g2	11	0	4.459431619	0.000858686	0.005274132
TR23624-c0_g1	175	8	4.451211112	2.66804E-40	2.03409E-38
TR895-c0_g1	87	4	4.442943496	7.54048E-21	2.82375E-19
TR12534-c0_g1	65	3	4.437405312	5.81408E-16	1.67822E-14
TR27611-c0_g1	65	3	4.437405312	5.81408E-16	1.67822E-14

TR842-c0_g1	43	2	4.426264755	4.748E-11	9.34286E-10
TR20320-c2_g1	1180	56	4.397216222	1.5016E-259	9.4555E-257
TR15689-c0_g1	147	7	4.392317423	6.38034E-34	4.25389E-32
TR26177-c0_g1	42	2	4.392317423	8.60237E-11	1.64884E-09
TR4887-c0_g2	42	2	4.392317423	8.60237E-11	1.64884E-09
TR11557-c0_g1	21	1	4.392317423	4.45646E-06	4.61986E-05
TR12683-c0_g1	21	1	4.392317423	4.45646E-06	4.61986E-05
TR24844-c0_g1	21	1	4.392317423	4.45646E-06	4.61986E-05
TR7955-c0_g1	21	1	4.392317423	4.45646E-06	4.61986E-05
TR11184-c0_g1	712	34	4.38827059	2.9523E-157	9.8418E-155
TR625-c0_g1	82	4	4.357552005	1.42693E-19	5.00201E-18
TR842-c0_g2	41	2	4.357552005	1.55973E-10	2.91081E-09
TR15481-c0_g1	81	4	4.339850003	2.57142E-19	8.88567E-18
TR22237-c0_g1	80	4	4.321928095	4.63508E-19	1.57921E-17
TR4887-c0_g1	40	2	4.321928095	2.83007E-10	5.19599E-09
TR15679-c0_g1	20	1	4.321928095	8.18526E-06	8.12852E-05
TR11058-c1_g1	10	0	4.321928095	0.001611108	0.009106037
TR11928-c0_g1	10	0	4.321928095	0.001611108	0.009106037
TR12210-c0_g1	10	0	4.321928095	0.001611108	0.009106037
TR1355-c0_g1	10	0	4.321928095	0.001611108	0.009106037
TR13720-c0_g1	10	0	4.321928095	0.001611108	0.009106037
TR14241-c0_g1	10	0	4.321928095	0.001611108	0.009106037
TR15082-c0_g1	10	0	4.321928095	0.001611108	0.009106037
TR15631-c0_g1	10	0	4.321928095	0.001611108	0.009106037
TR16486-c0_g1	10	0	4.321928095	0.001611108	0.009106037
TR16498-c0_g1	10	0	4.321928095	0.001611108	0.009106037
TR16948-c0_g1	10	0	4.321928095	0.001611108	0.009106037
TR19133-c0_g1	10	0	4.321928095	0.001611108	0.009106037
TR19573-c1_g1	10	0	4.321928095	0.001611108	0.009106037
TR19820-c0_g1	10	0	4.321928095	0.001611108	0.009106037
TR20034-c0_g1	10	0	4.321928095	0.001611108	0.009106037
TR212-c0_g1	10	0	4.321928095	0.001611108	0.009106037
TR21464-c0_g3	10	0	4.321928095	0.001611108	0.009106037
TR21644-c0_g2	10	0	4.321928095	0.001611108	0.009106037
TR23110-c0_g1	10	0	4.321928095	0.001611108	0.009106037
TR23197-c0_g1	10	0	4.321928095	0.001611108	0.009106037
TR2333-c0_g1	10	0	4.321928095	0.001611108	0.009106037
TR23379-c0_g1	10	0	4.321928095	0.001611108	0.009106037
TR24046-c0_g1	10	0	4.321928095	0.001611108	0.009106037
TR24914-c0_g1	10	0	4.321928095	0.001611108	0.009106037
TR25246-c0_g1	10	0	4.321928095	0.001611108	0.009106037
TR26022-c0_g1	10	0	4.321928095	0.001611108	0.009106037
TR26317-c0_g1	10	0	4.321928095	0.001611108	0.009106037
TR26337-c0_g1	10	0	4.321928095	0.001611108	0.009106037

TR27386-c0_g1	10	0	4.321928095	0.001611108	0.009106037
TR27883-c0_g1	10	0	4.321928095	0.001611108	0.009106037
TR4591-c0_g2	10	0	4.321928095	0.001611108	0.009106037
TR4859-c0_g1	10	0	4.321928095	0.001611108	0.009106037
TR5191-c0_g1	10	0	4.321928095	0.001611108	0.009106037
TR5364-c1_g1	10	0	4.321928095	0.001611108	0.009106037
TR5821-c0_g2	10	0	4.321928095	0.001611108	0.009106037
TR5917-c0_g1	10	0	4.321928095	0.001611108	0.009106037
TR6685-c0_g1	10	0	4.321928095	0.001611108	0.009106037
TR753-c0_g1	10	0	4.321928095	0.001611108	0.009106037
TR90-c0_g2	10	0	4.321928095	0.001611108	0.009106037
TR9180-c0_g1	10	0	4.321928095	0.001611108	0.009106037
TR9572-c0_g1	10	0	4.321928095	0.001611108	0.009106037
TR9655-c0_g2	10	0	4.321928095	0.001611108	0.009106037
TR20352-c0_g1	705	36	4.291554446	5.1781E-154	1.6303E-151
TR8516-c0_g1	117	6	4.285402219	5.06205E-27	2.55375E-25
TR9354-c0_g2	39	2	4.285402219	5.1387E-10	9.17697E-09
TR10737-c0_g1	19	1	4.247927513	1.50658E-05	0.000142616
TR11286-c0_g1	19	1	4.247927513	1.50658E-05	0.000142616
TR18430-c0_g1	19	1	4.247927513	1.50658E-05	0.000142616
TR2782-c0_g2	19	1	4.247927513	1.50658E-05	0.000142616
TR90-c0_g1	19	1	4.247927513	1.50658E-05	0.000142616
TR9328-c0_g1	19	1	4.247927513	1.50658E-05	0.000142616
TR7542-c0_g1	493	26	4.245004118	1.1206E-107	2.352E-105
TR10161-c0_g1	604	32	4.238404739	2.2537E-131	5.8054E-129
TR5649-c0_g1	93	5	4.217230716	1.22374E-21	4.78279E-20
TR14545-c0_g1	130	7	4.215012891	1.36394E-29	7.70392E-28
TR14914-c0_g1	464	25	4.214124805	4.6095E-101	9.3295E-99
TR12313-c0_g1	147	8	4.199672345	3.5305E-33	2.29096E-31
TR13280-c0_g1	90	5	4.169925001	7.17063E-21	2.69117E-19
TR7098-c0_g1	36	2	4.169925001	3.08878E-09	5.00605E-08
TR15645-c0_g1	18	1	4.169925001	2.77885E-05	0.000249705
TR18201-c0_g1	18	1	4.169925001	2.77885E-05	0.000249705
TR22383-c0_g1	18	1	4.169925001	2.77885E-05	0.000249705
TR22612-c0_g2	18	1	4.169925001	2.77885E-05	0.000249705
TR5523-c0_g2	18	1	4.169925001	2.77885E-05	0.000249705
TR5657-c0_g1	18	1	4.169925001	2.77885E-05	0.000249705
TR786-c0_g1	18	1	4.169925001	2.77885E-05	0.000249705
TR8806-c0_g1	18	1	4.169925001	2.77885E-05	0.000249705
TR10000-c0_g1	9	0	4.169925001	0.003042725	0.015332054
TR10171-c0_g2	9	0	4.169925001	0.003042725	0.015332054
TR10427-c0_g1	9	0	4.169925001	0.003042725	0.015332054
TR10610-c0_g1	9	0	4.169925001	0.003042725	0.015332054
TR11382-c0_g1	9	0	4.169925001	0.003042725	0.015332054

TR11439-c0_g1	9	0	4.169925001	0.003042725	0.015332054
TR11537-c0_g1	9	0	4.169925001	0.003042725	0.015332054
TR12182-c0_g1	9	0	4.169925001	0.003042725	0.015332054
TR12206-c0_g1	9	0	4.169925001	0.003042725	0.015332054
TR13-c0_g1	9	0	4.169925001	0.003042725	0.015332054
TR13675-c0_g1	9	0	4.169925001	0.003042725	0.015332054
TR14285-c0_g1	9	0	4.169925001	0.003042725	0.015332054
TR14666-c0_g1	9	0	4.169925001	0.003042725	0.015332054
TR14666-c0_g2	9	0	4.169925001	0.003042725	0.015332054
TR15230-c0_g1	9	0	4.169925001	0.003042725	0.015332054
TR15230-c0_g2	9	0	4.169925001	0.003042725	0.015332054
TR16112-c0_g1	9	0	4.169925001	0.003042725	0.015332054
TR16538-c0_g1	9	0	4.169925001	0.003042725	0.015332054
TR16600-c0_g1	9	0	4.169925001	0.003042725	0.015332054
TR16984-c0_g1	9	0	4.169925001	0.003042725	0.015332054
TR17127-c0_g1	9	0	4.169925001	0.003042725	0.015332054
TR18102-c0_g1	9	0	4.169925001	0.003042725	0.015332054
TR18214-c0_g1	9	0	4.169925001	0.003042725	0.015332054
TR18417-c0_g1	9	0	4.169925001	0.003042725	0.015332054
TR18608-c0_g1	9	0	4.169925001	0.003042725	0.015332054
TR18719-c0_g1	9	0	4.169925001	0.003042725	0.015332054
TR19016-c0_g2	9	0	4.169925001	0.003042725	0.015332054
TR19557-c0_g8	9	0	4.169925001	0.003042725	0.015332054
TR19900-c0_g1	9	0	4.169925001	0.003042725	0.015332054
TR2091-c0_g1	9	0	4.169925001	0.003042725	0.015332054
TR23621-c0_g1	9	0	4.169925001	0.003042725	0.015332054
TR23656-c0_g1	9	0	4.169925001	0.003042725	0.015332054
TR23691-c0_g2	9	0	4.169925001	0.003042725	0.015332054
TR24914-c0_g2	9	0	4.169925001	0.003042725	0.015332054
TR25859-c0_g2	9	0	4.169925001	0.003042725	0.015332054
TR26283-c0_g1	9	0	4.169925001	0.003042725	0.015332054
TR26381-c0_g1	9	0	4.169925001	0.003042725	0.015332054
TR27208-c0_g1	9	0	4.169925001	0.003042725	0.015332054
TR27474-c2_g1	9	0	4.169925001	0.003042725	0.015332054
TR2749-c0_g3	9	0	4.169925001	0.003042725	0.015332054
TR27567-c0_g1	9	0	4.169925001	0.003042725	0.015332054
TR27704-c0_g3	9	0	4.169925001	0.003042725	0.015332054
TR3149-c0_g1	9	0	4.169925001	0.003042725	0.015332054
TR3188-c0_g1	9	0	4.169925001	0.003042725	0.015332054
TR3459-c0_g1	9	0	4.169925001	0.003042725	0.015332054
TR42-c0_g1	9	0	4.169925001	0.003042725	0.015332054
TR4700-c0_g1	9	0	4.169925001	0.003042725	0.015332054
TR5045-c0_g1	9	0	4.169925001	0.003042725	0.015332054
TR5140-c0_g1	9	0	4.169925001	0.003042725	0.015332054

TR5335-c0_g1	9	0	4.169925001	0.003042725	0.015332054
TR5711-c0_g1	9	0	4.169925001	0.003042725	0.015332054
TR6731-c0_g2	9	0	4.169925001	0.003042725	0.015332054
TR6984-c0_g1	9	0	4.169925001	0.003042725	0.015332054
TR7327-c0_g1	9	0	4.169925001	0.003042725	0.015332054
TR8974-c0_g1	9	0	4.169925001	0.003042725	0.015332054
TR9511-c0_g1	9	0	4.169925001	0.003042725	0.015332054
TR9636-c0_g1	9	0	4.169925001	0.003042725	0.015332054
TR9636-c0_g2	9	0	4.169925001	0.003042725	0.015332054
TR9672-c0_g1	9	0	4.169925001	0.003042725	0.015332054
TR2894-c0_g2	539	30	4.167250867	2.2627E-116	5.3429E-114
TR1048-c0_g1	53	3	4.142957954	7.06989E-13	1.63757E-11
TR7132-c0_g1	35	2	4.129283017	5.6232E-09	8.82749E-08
TR18163-c0_g1	52	3	4.115477217	1.28074E-12	2.92665E-11
TR1489-c0_g2	34	2	4.087462841	1.02431E-08	1.56045E-07
TR13261-c0_g1	17	1	4.087462841	5.1362E-05	0.000433146
TR21231-c0_g1	17	1	4.087462841	5.1362E-05	0.000433146
TR22216-c0_g1	17	1	4.087462841	5.1362E-05	0.000433146
TR25132-c0_g1	17	1	4.087462841	5.1362E-05	0.000433146
TR27088-c0_g1	17	1	4.087462841	5.1362E-05	0.000433146
TR2742-c0_g2	17	1	4.087462841	5.1362E-05	0.000433146
TR3831-c0_g1	17	1	4.087462841	5.1362E-05	0.000433146
TR5900-c0_g1	17	1	4.087462841	5.1362E-05	0.000433146
TR2699-c0_g2	67	4	4.06608919	1.00342E-15	2.83381E-14
TR24851-c0_g1	99	6	4.044394119	2.01515E-22	8.11854E-21
TR6730-c0_g1	33	2	4.044394119	1.86689E-08	2.75517E-07
TR15479-c0_g1	49	3	4.029747343	7.62548E-12	1.64522E-10
TR2699-c0_g1	81	5	4.017921908	1.45153E-18	4.78255E-17
TR2704-c0_g1	80	5	4	2.61962E-18	8.5157E-17
TR13986-c0_g1	48	3	4	1.38268E-11	2.87026E-10
TR15635-c0_g1	48	3	4	1.38268E-11	2.87026E-10
TR12972-c0_g3	32	2	4	3.40428E-08	4.89655E-07
TR1489-c0_g1	32	2	4	3.40428E-08	4.89655E-07
TR14935-c0_g1	32	2	4	3.40428E-08	4.89655E-07
TR927-c0_g1	32	2	4	3.40428E-08	4.89655E-07
TR14258-c0_g1	16	1	4	9.51281E-05	0.000757753
TR17111-c0_g1	16	1	4	9.51281E-05	0.000757753
TR188-c0_g1	16	1	4	9.51281E-05	0.000757753
TR3247-c0_g1	16	1	4	9.51281E-05	0.000757753
TR5763-c0_g1	16	1	4	9.51281E-05	0.000757753
TR14776-c0_g1	47	3	3.969626351	2.50757E-11	5.10563E-10
TR5911-c0_g1	47	3	3.969626351	2.50757E-11	5.10563E-10
TR7098-c0_g2	31	2	3.95419631	6.21057E-08	8.58802E-07
TR5269-c0_g1	122	8	3.930737338	8.44413E-27	4.18547E-25

TR8581-c0_g1	406	27	3.910448415	7.5284E-85	1.34729E-82
TR16020-c0_g1	30	2	3.906890596	1.13348E-07	1.50788E-06
TR6154-c0_g1	30	2	3.906890596	1.13348E-07	1.50788E-06
TR1143-c0_g1	15	1	3.906890596	0.00017654	0.001318142
TR12711-c0_g2	15	1	3.906890596	0.00017654	0.001318142
TR20105-c0_g1	15	1	3.906890596	0.00017654	0.001318142
TR2091-c0_g2	15	1	3.906890596	0.00017654	0.001318142
TR22946-c0_g1	15	1	3.906890596	0.00017654	0.001318142
TR6156-c0_g1	15	1	3.906890596	0.00017654	0.001318142
TR6391-c0_g1	15	1	3.906890596	0.00017654	0.001318142
TR839-c0_g1	15	1	3.906890596	0.00017654	0.001318142
TR18436-c0_g1	44	3	3.874469118	1.49681E-10	2.79645E-09
TR15497-c0_g1	131	9	3.863498	2.37963E-28	1.27624E-26
TR22915-c0_g1	160	11	3.862496476	3.00064E-34	2.0164E-32
TR21278-c0_g1	28	2	3.807354922	3.77927E-07	4.71405E-06
TR10155-c0_g1	14	1	3.807354922	0.000328256	0.002301372
TR14402-c0_g1	14	1	3.807354922	0.000328256	0.002301372
TR14506-c0_g1	14	1	3.807354922	0.000328256	0.002301372
TR16038-c0_g1	14	1	3.807354922	0.000328256	0.002301372
TR16186-c0_g1	14	1	3.807354922	0.000328256	0.002301372
TR16674-c0_g1	14	1	3.807354922	0.000328256	0.002301372
TR19555-c0_g1	14	1	3.807354922	0.000328256	0.002301372
TR22274-c0_g1	14	1	3.807354922	0.000328256	0.002301372
TR22691-c0_g1	14	1	3.807354922	0.000328256	0.002301372
TR23552-c0_g1	14	1	3.807354922	0.000328256	0.002301372
TR23644-c0_g1	14	1	3.807354922	0.000328256	0.002301372
TR6232-c0_g1	14	1	3.807354922	0.000328256	0.002301372
TR9027-c0_g1	251	18	3.801618553	3.43825E-52	3.45887E-50
TR1938-c0_g1	41	3	3.772589504	8.93798E-10	1.55854E-08
TR10605-c0_g3	27	2	3.754887502	6.90328E-07	8.21884E-06
TR12122-c1_g1	27	2	3.754887502	6.90328E-07	8.21884E-06
TR12886-c0_g1	27	2	3.754887502	6.90328E-07	8.21884E-06
TR13957-c0_g1	27	2	3.754887502	6.90328E-07	8.21884E-06
TR14772-c0_g1	27	2	3.754887502	6.90328E-07	8.21884E-06
TR22612-c0_g1	27	2	3.754887502	6.90328E-07	8.21884E-06
TR4560-c0_g3	27	2	3.754887502	6.90328E-07	8.21884E-06
TR10549-c0_g1	160	12	3.736965594	1.63737E-33	1.06657E-31
TR5826-c0_g2	80	6	3.736965594	1.45916E-17	4.59401E-16
TR19951-c0_g1	40	3	3.736965594	1.62137E-09	2.70781E-08
TR8264-c0_g9	4325	326	3.729756263	0	0
TR2579-c5_g1	670	51	3.715591943	4.8219E-134	1.2809E-131
TR7780-c0_g1	65	5	3.700439718	1.83161E-14	4.76144E-13
TR537-c0_g1	39	3	3.700439718	2.94082E-09	4.78448E-08
TR26712-c1_g1	26	2	3.700439718	1.26112E-06	1.43034E-05

TR5431-c0_g1	26	2	3.700439718	1.26112E-06	1.43034E-05
TR8106-c0_g1	26	2	3.700439718	1.26112E-06	1.43034E-05
TR870-c0_g1	26	2	3.700439718	1.26112E-06	1.43034E-05
TR16233-c0_g2	13	1	3.700439718	0.000611466	0.003949748
TR18031-c0_g1	13	1	3.700439718	0.000611466	0.003949748
TR1811-c0_g1	13	1	3.700439718	0.000611466	0.003949748
TR20681-c0_g1	13	1	3.700439718	0.000611466	0.003949748
TR21303-c0_g2	13	1	3.700439718	0.000611466	0.003949748
TR25240-c0_g1	13	1	3.700439718	0.000611466	0.003949748
TR26880-c1_g1	13	1	3.700439718	0.000611466	0.003949748
TR27646-c0_g1	13	1	3.700439718	0.000611466	0.003949748
TR27707-c0_g2	13	1	3.700439718	0.000611466	0.003949748
TR2848-c0_g1	13	1	3.700439718	0.000611466	0.003949748
TR5523-c0_g1	13	1	3.700439718	0.000611466	0.003949748
TR6017-c0_g1	13	1	3.700439718	0.000611466	0.003949748
TR71-c0_g1	13	1	3.700439718	0.000611466	0.003949748
TR8314-c0_g2	13	1	3.700439718	0.000611466	0.003949748
TR997-c0_g1	13	1	3.700439718	0.000611466	0.003949748
TR12859-c6_g1	673	52	3.694022976	4.4776E-134	1.2083E-131
TR25215-c0_g1	38	3	3.662965013	5.333E-09	8.38742E-08
TR27791-c0_g1	38	3	3.662965013	5.333E-09	8.38742E-08
TR4117-c0_g1	38	3	3.662965013	5.333E-09	8.38742E-08
TR7217-c0_g1	38	3	3.662965013	5.333E-09	8.38742E-08
TR17623-c0_g1	202	16	3.658211483	3.05671E-41	2.41712E-39
TR13621-c0_g1	25	2	3.64385619	2.30394E-06	2.50287E-05
TR19698-c0_g1	25	2	3.64385619	2.30394E-06	2.50287E-05
TR20825-c0_g1	25	2	3.64385619	2.30394E-06	2.50287E-05
TR3346-c0_g1	25	2	3.64385619	2.30394E-06	2.50287E-05
TR870-c0_g2	25	2	3.64385619	2.30394E-06	2.50287E-05
TR923-c0_g1	25	2	3.64385619	2.30394E-06	2.50287E-05
TR21965-c0_g1	37	3	3.624490865	9.66866E-09	1.47426E-07
TR22232-c0_g1	37	3	3.624490865	9.66866E-09	1.47426E-07
TR3265-c0_g1	37	3	3.624490865	9.66866E-09	1.47426E-07
TR623-c0_g1	37	3	3.624490865	9.66866E-09	1.47426E-07
TR9354-c0_g1	37	3	3.624490865	9.66866E-09	1.47426E-07
TR24223-c0_g1	49	4	3.614709844	4.26013E-11	8.45133E-10
TR7682-c0_g1	193	16	3.592457037	5.73116E-39	4.27357E-37
TR7284-c0_g1	60	5	3.584962501	3.47526E-13	8.3217E-12
TR1878-c0_g1	36	3	3.584962501	1.75237E-08	2.59066E-07
TR10978-c0_g1	24	2	3.584962501	4.20869E-06	4.37636E-05
TR10978-c0_g2	24	2	3.584962501	4.20869E-06	4.37636E-05
TR27553-c0_g1	24	2	3.584962501	4.20869E-06	4.37636E-05
TR4925-c0_g1	24	2	3.584962501	4.20869E-06	4.37636E-05
TR5431-c0_g2	24	2	3.584962501	4.20869E-06	4.37636E-05

TR14148-c0_g1	12	1	3.584962501	0.001140939	0.006775478
TR16575-c0_g1	12	1	3.584962501	0.001140939	0.006775478
TR17267-c0_g1	12	1	3.584962501	0.001140939	0.006775478
TR18753-c0_g1	12	1	3.584962501	0.001140939	0.006775478
TR19750-c0_g1	12	1	3.584962501	0.001140939	0.006775478
TR19858-c0_g1	12	1	3.584962501	0.001140939	0.006775478
TR20148-c0_g1	12	1	3.584962501	0.001140939	0.006775478
TR2102-c0_g1	12	1	3.584962501	0.001140939	0.006775478
TR2183-c0_g1	12	1	3.584962501	0.001140939	0.006775478
TR25464-c0_g2	12	1	3.584962501	0.001140939	0.006775478
TR8178-c1_g2	12	1	3.584962501	0.001140939	0.006775478
TR8314-c0_g1	12	1	3.584962501	0.001140939	0.006775478
TR5445-c0_g2	35	3	3.544320516	3.17482E-08	4.59763E-07
TR7775-c0_g1	128	11	3.540568381	3.92784E-26	1.89174E-24
TR13258-c0_g1	81	7	3.532495081	4.31371E-17	1.32381E-15
TR15934-c0_g1	81	7	3.532495081	4.31371E-17	1.32381E-15
TR4493-c0_g2	46	4	3.523561956	2.50216E-10	4.60391E-09
TR14815-c0_g1	23	2	3.523561956	7.68658E-06	7.66914E-05
TR22279-c0_g1	23	2	3.523561956	7.68658E-06	7.66914E-05
TR24258-c0_g1	23	2	3.523561956	7.68658E-06	7.66914E-05
TR27216-c1_g1	23	2	3.523561956	7.68658E-06	7.66914E-05
TR27563-c0_g1	23	2	3.523561956	7.68658E-06	7.66914E-05
TR379-c0_g1	23	2	3.523561956	7.68658E-06	7.66914E-05
TR4061-c0_g1	23	2	3.523561956	7.68658E-06	7.66914E-05
TR12824-c7_g1	16194	1418	3.513529945	0	0
TR2894-c0_g1	251	22	3.512111935	2.62332E-49	2.51977E-47
TR17479-c0_g1	68	6	3.502500341	1.6646E-14	4.34721E-13
TR12850-c0_g1	34	3	3.502500341	5.74927E-08	8.0119E-07
TR17957-c0_g1	34	3	3.502500341	5.74927E-08	8.0119E-07
TR22295-c0_g1	34	3	3.502500341	5.74927E-08	8.0119E-07
TR293-c0_g1	34	3	3.502500341	5.74927E-08	8.0119E-07
TR17479-c0_g4	147	13	3.499232627	1.65964E-29	9.34306E-28
TR15490-c0_g1	67	6	3.48112669	2.98625E-14	7.6692E-13
TR1657-c0_g1	67	6	3.48112669	2.98625E-14	7.6692E-13
TR22304-c0_g1	67	6	3.48112669	2.98625E-14	7.6692E-13
TR10161-c0_g2	266	24	3.470319935	1.1453E-51	1.14539E-49
TR22967-c0_g1	66	6	3.459431619	5.35484E-14	1.34475E-12
TR4875-c0_g1	66	6	3.459431619	5.35484E-14	1.34475E-12
TR4912-c0_g1	55	5	3.459431619	6.53481E-12	1.41169E-10
TR13900-c0_g1	33	3	3.459431619	1.04057E-07	1.3908E-06
TR17879-c0_g1	33	3	3.459431619	1.04057E-07	1.3908E-06
TR653-c0_g1	33	3	3.459431619	1.04057E-07	1.3908E-06
TR19688-c0_g1	22	2	3.459431619	1.40335E-05	0.000133738
TR24255-c1_g1	22	2	3.459431619	1.40335E-05	0.000133738

TR26197-c0_g1	22	2	3.459431619	1.40335E-05	0.000133738
TR1023-c0_g1	11	1	3.459431619	0.002132049	0.011448734
TR1156-c0_g1	11	1	3.459431619	0.002132049	0.011448734
TR14458-c0_g1	11	1	3.459431619	0.002132049	0.011448734
TR15375-c0_g1	11	1	3.459431619	0.002132049	0.011448734
TR15922-c0_g1	11	1	3.459431619	0.002132049	0.011448734
TR16796-c0_g1	11	1	3.459431619	0.002132049	0.011448734
TR17522-c0_g1	11	1	3.459431619	0.002132049	0.011448734
TR18497-c0_g1	11	1	3.459431619	0.002132049	0.011448734
TR19039-c0_g2	11	1	3.459431619	0.002132049	0.011448734
TR19428-c0_g1	11	1	3.459431619	0.002132049	0.011448734
TR1955-c0_g1	11	1	3.459431619	0.002132049	0.011448734
TR20123-c0_g2	11	1	3.459431619	0.002132049	0.011448734
TR20652-c0_g1	11	1	3.459431619	0.002132049	0.011448734
TR22561-c0_g2	11	1	3.459431619	0.002132049	0.011448734
TR23306-c0_g1	11	1	3.459431619	0.002132049	0.011448734
TR23576-c0_g1	11	1	3.459431619	0.002132049	0.011448734
TR24016-c0_g1	11	1	3.459431619	0.002132049	0.011448734
TR25132-c0_g2	11	1	3.459431619	0.002132049	0.011448734
TR25464-c0_g1	11	1	3.459431619	0.002132049	0.011448734
TR25821-c0_g1	11	1	3.459431619	0.002132049	0.011448734
TR26260-c0_g1	11	1	3.459431619	0.002132049	0.011448734
TR27664-c0_g2	11	1	3.459431619	0.002132049	0.011448734
TR27786-c0_g1	11	1	3.459431619	0.002132049	0.011448734
TR3059-c0_g1	11	1	3.459431619	0.002132049	0.011448734
TR3691-c0_g2	11	1	3.459431619	0.002132049	0.011448734
TR732-c0_g2	11	1	3.459431619	0.002132049	0.011448734
TR9130-c0_g1	11	1	3.459431619	0.002132049	0.011448734
TR12854-c0_g1	120	11	3.447458977	4.07483E-24	1.7855E-22
TR12265-c0_g2	54	5	3.432959407	1.17341E-11	2.46901E-10
TR24933-c0_g1	268	25	3.422233001	1.7909E-51	1.78056E-49
TR5151-c0_g1	32	3	3.415037499	1.88214E-07	2.44079E-06
TR8507-c3_g2	1956	184	3.410128699	0	0
TR13376-c0_g1	42	4	3.392317423	2.63296E-09	4.29594E-08
TR16446-c0_g1	21	2	3.392317423	2.56081E-05	0.000232943
TR19849-c0_g1	21	2	3.392317423	2.56081E-05	0.000232943
TR22168-c0_g2	21	2	3.392317423	2.56081E-05	0.000232943
TR11265-c0_g1	125	12	3.380821784	1.11781E-24	4.98797E-23
TR8507-c3_g1	1916	184	3.38031989	0	0
TR4493-c0_g1	52	5	3.378511623	3.77675E-11	7.53636E-10
TR10283-c1_g3	31	3	3.36923381	3.40183E-07	4.26516E-06
TR17111-c0_g2	31	3	3.36923381	3.40183E-07	4.26516E-06
TR5696-c0_g1	31	3	3.36923381	3.40183E-07	4.26516E-06
TR8264-c0_g7	102	10	3.350497247	2.73576E-20	9.89607E-19

TR12265-c0_g1	51	5	3.350497247	6.76905E-11	1.30627E-09
TR3825-c0_g1	51	5	3.350497247	6.76905E-11	1.30627E-09
TR8006-c0_g1	51	5	3.350497247	6.76905E-11	1.30627E-09
TR14900-c0_g1	81	8	3.339850003	2.15847E-16	6.38206E-15
TR7289-c0_g1	101	10	3.336283388	4.86992E-20	1.74306E-18
TR11197-c0_g3	50	5	3.321928095	1.21234E-10	2.2775E-09
TR10151-c0_g2	20	2	3.321928095	4.66961E-05	0.000397544
TR13354-c0_g1	20	2	3.321928095	4.66961E-05	0.000397544
TR14799-c0_g1	20	2	3.321928095	4.66961E-05	0.000397544
TR14913-c0_g1	20	2	3.321928095	4.66961E-05	0.000397544
TR17841-c1_g1	20	2	3.321928095	4.66961E-05	0.000397544
TR26197-c0_g2	20	2	3.321928095	4.66961E-05	0.000397544
TR26872-c0_g1	20	2	3.321928095	4.66961E-05	0.000397544
TR4146-c0_g1	20	2	3.321928095	4.66961E-05	0.000397544
TR9286-c0_g1	20	2	3.321928095	4.66961E-05	0.000397544
TR1132-c0_g1	10	1	3.321928095	0.003988987	0.019376588
TR12465-c0_g1	10	1	3.321928095	0.003988987	0.019376588
TR1296-c0_g1	10	1	3.321928095	0.003988987	0.019376588
TR14691-c0_g1	10	1	3.321928095	0.003988987	0.019376588
TR15730-c0_g2	10	1	3.321928095	0.003988987	0.019376588
TR17534-c0_g1	10	1	3.321928095	0.003988987	0.019376588
TR18762-c0_g1	10	1	3.321928095	0.003988987	0.019376588
TR18932-c0_g1	10	1	3.321928095	0.003988987	0.019376588
TR205-c0_g1	10	1	3.321928095	0.003988987	0.019376588
TR20838-c0_g2	10	1	3.321928095	0.003988987	0.019376588
TR21387-c0_g1	10	1	3.321928095	0.003988987	0.019376588
TR22412-c0_g1	10	1	3.321928095	0.003988987	0.019376588
TR22561-c0_g1	10	1	3.321928095	0.003988987	0.019376588
TR23224-c0_g1	10	1	3.321928095	0.003988987	0.019376588
TR23628-c0_g1	10	1	3.321928095	0.003988987	0.019376588
TR23924-c0_g1	10	1	3.321928095	0.003988987	0.019376588
TR24172-c0_g2	10	1	3.321928095	0.003988987	0.019376588
TR25829-c0_g1	10	1	3.321928095	0.003988987	0.019376588
TR26049-c0_g1	10	1	3.321928095	0.003988987	0.019376588
TR26358-c0_g1	10	1	3.321928095	0.003988987	0.019376588
TR27146-c0_g1	10	1	3.321928095	0.003988987	0.019376588
TR27251-c0_g1	10	1	3.321928095	0.003988987	0.019376588
TR27468-c0_g3	10	1	3.321928095	0.003988987	0.019376588
TR2848-c0_g2	10	1	3.321928095	0.003988987	0.019376588
TR3783-c0_g1	10	1	3.321928095	0.003988987	0.019376588
TR3783-c0_g2	10	1	3.321928095	0.003988987	0.019376588
TR3812-c0_g1	10	1	3.321928095	0.003988987	0.019376588
TR4902-c0_g1	10	1	3.321928095	0.003988987	0.019376588
TR5437-c0_g2	10	1	3.321928095	0.003988987	0.019376588

TR647-c0_g1	10	1	3.321928095	0.003988987	0.019376588
TR8127-c0_g1	10	1	3.321928095	0.003988987	0.019376588
TR9033-c0_g2	10	1	3.321928095	0.003988987	0.019376588
TR11198-c0_g1	89	9	3.30580843	1.02454E-17	3.23764E-16
TR21092-c0_g1	49	5	3.292781749	2.16964E-10	4.00508E-09
TR8535-c0_g1	39	4	3.285402219	1.52695E-08	2.26925E-07
TR16584-c0_g1	29	3	3.273018494	1.10835E-06	1.27766E-05
TR19824-c0_g1	29	3	3.273018494	1.10835E-06	1.27766E-05
TR24228-c0_g1	29	3	3.273018494	1.10835E-06	1.27766E-05
TR10081-c0_g1	144	15	3.263034406	2.20448E-27	1.13918E-25
TR10318-c1_g16	1581	165	3.260299438	1.3916E-282	1.0286E-279
TR1450-c0_g2	19	2	3.247927513	8.50715E-05	0.000684166
TR17241-c0_g1	19	2	3.247927513	8.50715E-05	0.000684166
TR23265-c0_g2	19	2	3.247927513	8.50715E-05	0.000684166
TR7662-c0_g1	19	2	3.247927513	8.50715E-05	0.000684166
TR8179-c3_g8	19	2	3.247927513	8.50715E-05	0.000684166
TR839-c0_g2	19	2	3.247927513	8.50715E-05	0.000684166
TR9798-c0_g1	19	2	3.247927513	8.50715E-05	0.000684166
TR27508-c0_g1	66	7	3.237039197	2.60879E-13	6.29118E-12
TR9585-c0_g1	66	7	3.237039197	2.60879E-13	6.29118E-12
TR4019-c0_g1	1302	139	3.22757266	5.3093E-231	2.9118E-228
TR880-c0_g1	28	3	3.222392421	1.99742E-06	2.19656E-05
TR23223-c0_g2	121	13	3.218423519	5.24887E-23	2.17653E-21
TR2807-c0_g1	341	37	3.204174563	1.63884E-61	2.03376E-59
TR9388-c1_g1	343	38	3.174137253	2.36985E-61	2.89861E-59
TR4493-c0_g3	63	7	3.169925001	1.46378E-12	3.32704E-11
TR14480-c0_g1	54	6	3.169925001	5.64586E-11	1.10203E-09
TR18721-c0_g1	54	6	3.169925001	5.64586E-11	1.10203E-09
TR22283-c0_g1	54	6	3.169925001	5.64586E-11	1.10203E-09
TR19097-c0_g2	27	3	3.169925001	3.59515E-06	3.80587E-05
TR20825-c0_g2	27	3	3.169925001	3.59515E-06	3.80587E-05
TR3160-c0_g1	27	3	3.169925001	3.59515E-06	3.80587E-05
TR6153-c0_g2	27	3	3.169925001	3.59515E-06	3.80587E-05
TR6701-c0_g1	27	3	3.169925001	3.59515E-06	3.80587E-05
TR9161-c0_g1	27	3	3.169925001	3.59515E-06	3.80587E-05
TR10523-c0_g1	18	2	3.169925001	0.000154802	0.001173357
TR24514-c0_g1	18	2	3.169925001	0.000154802	0.001173357
TR833-c0_g2	457	51	3.163625013	6.85308E-81	1.14227E-78
TR14478-c0_g1	80	9	3.152003093	1.78827E-15	5.00873E-14
TR22384-c0_g3	62	7	3.146841388	2.59679E-12	5.79381E-11
TR11702-c9_g1	9810	1113	3.139799544	0	0
TR11197-c0_g1	44	5	3.137503524	3.92904E-09	6.29585E-08
TR6703-c0_g1	79	9	3.133855747	3.16301E-15	8.75821E-14
TR7512-c0_g1	140	16	3.129283017	9.72144E-26	4.57832E-24

TR16338-c0_g1	35	4	3.129283017	1.5691E-07	2.06888E-06
TR6217-c0_g1	35	4	3.129283017	1.5691E-07	2.06888E-06
TR8135-c0_g1	35	4	3.129283017	1.5691E-07	2.06888E-06
TR5152-c0_g3	96	11	3.125530882	3.93547E-18	1.27202E-16
TR7303-c0_g1	148	17	3.121990524	4.63187E-27	2.34369E-25
TR22299-c0_g1	182	21	3.115477217	7.80367E-33	4.95047E-31
TR4863-c0_g1	458	53	3.111283334	7.49308E-80	1.22493E-77
TR11248-c0_g1	43	5	3.10433666	6.99001E-09	1.0843E-07
TR12931-c0_g1	17	2	3.087462841	0.000281275	0.001998436
TR16657-c0_g2	17	2	3.087462841	0.000281275	0.001998436
TR23265-c0_g1	17	2	3.087462841	0.000281275	0.001998436
TR7884-c0_g1	17	2	3.087462841	0.000281275	0.001998436
TR9506-c0_g1	17	2	3.087462841	0.000281275	0.001998436
TR5826-c0_g1	118	14	3.075288127	1.28134E-21	4.99644E-20
TR3987-c0_g1	84	10	3.070389328	8.11676E-16	2.30762E-14
TR11197-c0_g2	42	5	3.070389328	1.24202E-08	1.86702E-07
TR16323-c0_g1	42	5	3.070389328	1.24202E-08	1.86702E-07
TR17319-c0_g1	42	5	3.070389328	1.24202E-08	1.86702E-07
TR21295-c1_g1	42	5	3.070389328	1.24202E-08	1.86702E-07
TR10318-c1_g9	842	101	3.05946494	5.9239E-143	1.707E-140
TR9798-c0_g2	25	3	3.058893689	1.15953E-05	0.000112584
TR4211-c0_g1	66	8	3.044394119	1.17034E-12	2.68158E-11
TR13163-c0_g1	33	4	3.044394119	4.99122E-07	6.08297E-06
TR27576-c0_g1	33	4	3.044394119	4.99122E-07	6.08297E-06
TR6640-c0_g1	33	4	3.044394119	4.99122E-07	6.08297E-06
TR2616-c3_g1	280	34	3.041820176	1.62737E-48	1.52859E-46
TR18817-c0_g1	82	10	3.03562391	2.51513E-15	6.98701E-14
TR9270-c0_g1	41	5	3.03562391	2.20394E-08	3.23575E-07
TR14645-c0_g1	147	18	3.029747343	3.49865E-26	1.70246E-24
TR10373-c3_g2	49	6	3.029747343	9.8971E-10	1.70826E-08
TR12211-c0_g1	49	6	3.029747343	9.8971E-10	1.70826E-08
TR14956-c0_g1	163	20	3.026800059	7.92366E-29	4.31771E-27
TR10479-c0_g1	73	9	3.019899557	9.52368E-14	2.37412E-12
TR11702-c0_g1	234	29	3.012383724	1.81418E-40	1.40838E-38
TR12542-c2_g1	9022	1119	3.01123725	0	0
TR2442-c0_g2	72	9	3	1.67461E-13	4.09649E-12
TR12325-c0_g1	48	6	3	1.74876E-09	2.90343E-08
TR26982-c0_g1	40	5	3	3.90529E-08	5.57474E-07
TR16889-c0_g1	32	4	3	8.88102E-07	1.03701E-05
TR25188-c0_g1	32	4	3	8.88102E-07	1.03701E-05
TR15017-c0_g1	24	3	3	2.07697E-05	0.000191909
TR325-c0_g1	24	3	3	2.07697E-05	0.000191909
TR9166-c0_g1	24	3	3	2.07697E-05	0.000191909
TR12711-c0_g1	16	2	3	0.00051015	0.003365626

TR15977-c0_g1	16	2	3	0.00051015	0.003365626
TR16023-c0_g1	16	2	3	0.00051015	0.003365626
TR16657-c0_g1	16	2	3	0.00051015	0.003365626
TR1991-c0_g1	16	2	3	0.00051015	0.003365626
TR24749-c0_g1	16	2	3	0.00051015	0.003365626
TR26151-c0_g1	16	2	3	0.00051015	0.003365626
TR26201-c0_g1	16	2	3	0.00051015	0.003365626
TR3754-c0_g1	16	2	3	0.00051015	0.003365626
TR7352-c0_g2	16	2	3	0.00051015	0.003365626
TR9708-c0_g1	16	2	3	0.00051015	0.003365626
TR12265-c0_g3	71	9	2.979822118	2.94173E-13	7.05406E-12
TR4594-c0_g1	47	6	2.969626351	3.08581E-09	5.00599E-08
TR466-c0_g1	39	5	2.963474124	6.90947E-08	9.42022E-07
TR25172-c0_g1	31	4	2.95419631	1.57745E-06	1.76671E-05
TR3076-c0_g1	209	27	2.95247163	1.18191E-35	8.33776E-34
TR13796-c0_g1	46	6	2.938599455	5.43743E-09	8.54376E-08
TR1045-c0_g1	23	3	2.938599455	3.71273E-05	0.000327053
TR24256-c0_g1	23	3	2.938599455	3.71273E-05	0.000327053
TR9228-c0_g1	23	3	2.938599455	3.71273E-05	0.000327053
TR19601-c0_g1	98	13	2.914270126	2.27119E-17	7.11108E-16
TR26909-c0_g1	978	130	2.911322842	5.6239E-158	1.9513E-155
TR26180-c0_g1	60	8	2.906890596	3.44966E-11	6.93248E-10
TR14482-c0_g1	30	4	2.906890596	2.79649E-06	3.01676E-05
TR15465-c0_g1	30	4	2.906890596	2.79649E-06	3.01676E-05
TR12842-c0_g1	15	2	2.906890596	0.000923206	0.005629744
TR14410-c0_g1	15	2	2.906890596	0.000923206	0.005629744
TR18389-c0_g1	15	2	2.906890596	0.000923206	0.005629744
TR20838-c0_g1	15	2	2.906890596	0.000923206	0.005629744
TR3582-c1_g1	15	2	2.906890596	0.000923206	0.005629744
TR6156-c0_g2	15	2	2.906890596	0.000923206	0.005629744
TR6681-c0_g1	15	2	2.906890596	0.000923206	0.005629744
TR7449-c0_g1	15	2	2.906890596	0.000923206	0.005629744
TR7621-c0_g1	15	2	2.906890596	0.000923206	0.005629744
TR786-c0_g2	15	2	2.906890596	0.000923206	0.005629744
TR10321-c4_g3	179	24	2.898853277	2.99254E-30	1.73052E-28
TR11135-c0_g1	929	125	2.893750502	2.9355E-149	8.9121E-147
TR539-c3_g1	111	15	2.887525271	2.65634E-19	9.1605E-18
TR13788-c0_g1	37	5	2.887525271	2.15204E-07	2.7781E-06
TR4009-c0_g1	37	5	2.887525271	2.15204E-07	2.7781E-06
TR10293-c0_g13	458	62	2.885007478	2.52301E-74	3.76267E-72
TR7788-c0_g1	198	27	2.874469118	5.15636E-33	3.32064E-31
TR8766-c0_g2	44	6	2.874469118	1.68056E-08	2.48883E-07
TR13156-c0_g1	22	3	2.874469118	6.62154E-05	0.000545686
TR1898-c0_g1	22	3	2.874469118	6.62154E-05	0.000545686

TR21822-c0_g1	22	3	2.874469118	6.62154E-05	0.000545686
TR22404-c0_g2	22	3	2.874469118	6.62154E-05	0.000545686
TR24976-c0_g2	22	3	2.874469118	6.62154E-05	0.000545686
TR7184-c0_g1	22	3	2.874469118	6.62154E-05	0.000545686
TR15412-c0_g1	95	13	2.86941589	1.20084E-16	3.58804E-15
TR10097-c2_g1	109	15	2.861293729	8.02963E-19	2.69791E-17
TR17879-c1_g1	29	4	2.857980995	4.94726E-06	5.09757E-05
TR22931-c0_g1	29	4	2.857980995	4.94726E-06	5.09757E-05
TR23828-c0_g1	29	4	2.857980995	4.94726E-06	5.09757E-05
TR27358-c0_g1	29	4	2.857980995	4.94726E-06	5.09757E-05
TR16785-c0_g1	65	9	2.852442812	8.4516E-12	1.80968E-10
TR15953-c4_g1	180	25	2.847996907	6.74764E-30	3.86258E-28
TR17051-c0_g1	36	5	2.847996907	3.78758E-07	4.71841E-06
TR7806-c0_g1	200	28	2.836501268	6.66149E-33	4.25768E-31
TR6523-c0_g1	695	98	2.826159323	1.6104E-109	3.4656E-107
TR21423-c0_g4	184	26	2.823122238	2.89387E-30	1.67917E-28
TR5571-c0_g1	63	9	2.807354922	2.56376E-11	5.21379E-10
TR6175-c0_g1	49	7	2.807354922	4.05164E-09	6.48008E-08
TR13669-c0_g1	35	5	2.807354922	6.65278E-07	7.99336E-06
TR261-c0_g1	35	5	2.807354922	6.65278E-07	7.99336E-06
TR16434-c0_g1	28	4	2.807354922	8.73222E-06	8.60634E-05
TR10334-c0_g3	21	3	2.807354922	0.000117788	0.000921561
TR10592-c0_g1	21	3	2.807354922	0.000117788	0.000921561
TR14823-c0_g1	21	3	2.807354922	0.000117788	0.000921561
TR27638-c0_g1	21	3	2.807354922	0.000117788	0.000921561
TR3922-c0_g1	21	3	2.807354922	0.000117788	0.000921561
TR11683-c0_g1	14	2	2.807354922	0.001666191	0.009342828
TR11736-c0_g1	14	2	2.807354922	0.001666191	0.009342828
TR13245-c0_g1	14	2	2.807354922	0.001666191	0.009342828
TR1450-c0_g1	14	2	2.807354922	0.001666191	0.009342828
TR14632-c0_g1	14	2	2.807354922	0.001666191	0.009342828
TR24819-c0_g1	14	2	2.807354922	0.001666191	0.009342828
TR26156-c0_g1	14	2	2.807354922	0.001666191	0.009342828
TR26743-c0_g1	14	2	2.807354922	0.001666191	0.009342828
TR392-c0_g1	14	2	2.807354922	0.001666191	0.009342828
TR5493-c0_g2	14	2	2.807354922	0.001666191	0.009342828
TR6153-c0_g1	14	2	2.807354922	0.001666191	0.009342828
TR1136-c0_g1	139	20	2.797012978	4.68403E-23	1.95183E-21
TR9466-c1_g1	708	103	2.781105023	1.0294E-109	2.2438E-107
TR16773-c0_g1	34	5	2.765534746	1.16603E-06	1.33856E-05
TR9456-c0_g1	34	5	2.765534746	1.16603E-06	1.33856E-05
TR157-c0_g1	27	4	2.754887502	1.53746E-05	0.000145216
TR20732-c0_g1	27	4	2.754887502	1.53746E-05	0.000145216
TR19680-c0_g1	121	18	2.748938236	6.13559E-20	2.17319E-18

TR24012-c0_g1	47	7	2.74723393	1.22934E-08	1.85452E-07
TR21010-c0_g1	715	107	2.740332445	4.2805E-109	9.0967E-107
TR16294-c0_g2	20	3	2.736965594	0.000208919	0.001545647
TR2109-c0_g1	20	3	2.736965594	0.000208919	0.001545647
TR2109-c0_g3	20	3	2.736965594	0.000208919	0.001545647
TR23208-c0_g1	20	3	2.736965594	0.000208919	0.001545647
TR27198-c0_g1	20	3	2.736965594	0.000208919	0.001545647
TR4172-c0_g1	20	3	2.736965594	0.000208919	0.001545647
TR526-c0_g1	20	3	2.736965594	0.000208919	0.001545647
TR9501-c0_g1	20	3	2.736965594	0.000208919	0.001545647
TR11293-c0_g1	53	8	2.727920455	1.68513E-09	2.80327E-08
TR24175-c0_g1	53	8	2.727920455	1.68513E-09	2.80327E-08
TR833-c0_g1	311	47	2.726181918	3.13494E-48	2.91246E-46
TR24051-c0_g1	46	7	2.716207034	2.13557E-08	3.14079E-07
TR2582-c4_g1	1107	170	2.703048571	1.9299E-165	7.1328E-163
TR12558-c1_g1	78	12	2.700439718	3.58721E-13	8.57769E-12
TR10293-c0_g17	39	6	2.700439718	2.73567E-07	3.46572E-06
TR14866-c0_g1	26	4	2.700439718	2.69961E-05	0.000244263
TR157-c0_g2	26	4	2.700439718	2.69961E-05	0.000244263
TR6808-c0_g2	26	4	2.700439718	2.69961E-05	0.000244263
TR10318-c2_g1	13	2	2.700439718	0.002997266	0.015332054
TR11044-c0_g1	13	2	2.700439718	0.002997266	0.015332054
TR11683-c0_g2	13	2	2.700439718	0.002997266	0.015332054
TR12165-c0_g1	13	2	2.700439718	0.002997266	0.015332054
TR14020-c0_g2	13	2	2.700439718	0.002997266	0.015332054
TR14170-c0_g1	13	2	2.700439718	0.002997266	0.015332054
TR14809-c0_g1	13	2	2.700439718	0.002997266	0.015332054
TR15527-c0_g1	13	2	2.700439718	0.002997266	0.015332054
TR16411-c0_g1	13	2	2.700439718	0.002997266	0.015332054
TR22168-c0_g1	13	2	2.700439718	0.002997266	0.015332054
TR24018-c0_g1	13	2	2.700439718	0.002997266	0.015332054
TR24325-c0_g2	13	2	2.700439718	0.002997266	0.015332054
TR25173-c0_g1	13	2	2.700439718	0.002997266	0.015332054
TR25173-c0_g2	13	2	2.700439718	0.002997266	0.015332054
TR26323-c0_g1	13	2	2.700439718	0.002997266	0.015332054
TR347-c0_g1	13	2	2.700439718	0.002997266	0.015332054
TR3756-c0_g1	13	2	2.700439718	0.002997266	0.015332054
TR4068-c0_g1	13	2	2.700439718	0.002997266	0.015332054
TR4847-c0_g1	13	2	2.700439718	0.002997266	0.015332054
TR4847-c0_g2	13	2	2.700439718	0.002997266	0.015332054
TR6154-c0_g2	13	2	2.700439718	0.002997266	0.015332054
TR71-c0_g2	13	2	2.700439718	0.002997266	0.015332054
TR8112-c0_g1	13	2	2.700439718	0.002997266	0.015332054
TR9400-c0_g2	13	2	2.700439718	0.002997266	0.015332054

TR4863-c0_g2	310	48	2.691161905	1.92666E-47	1.76106E-45
TR3949-c0_g1	64	10	2.678071905	5.52752E-11	1.08391E-09
TR10321-c4_g1	211	33	2.676705069	1.1667E-32	7.31935E-31
TR14515-c0_g1	38	6	2.662965013	4.74983E-07	5.80125E-06
TR20177-c0_g1	19	3	2.662965013	0.00036934	0.002549442
TR21734-c0_g1	19	3	2.662965013	0.00036934	0.002549442
TR22384-c0_g1	19	3	2.662965013	0.00036934	0.002549442
TR22654-c0_g2	19	3	2.662965013	0.00036934	0.002549442
TR24524-c0_g1	19	3	2.662965013	0.00036934	0.002549442
TR25298-c0_g1	19	3	2.662965013	0.00036934	0.002549442
TR640-c0_g1	19	3	2.662965013	0.00036934	0.002549442
TR9331-c0_g1	19	3	2.662965013	0.00036934	0.002549442
TR942-c0_g1	19	3	2.662965013	0.00036934	0.002549442
TR9865-c0_g1	19	3	2.662965013	0.00036934	0.002549442
TR13310-c0_g1	44	7	2.652076697	6.40655E-08	8.81942E-07
TR18947-c0_g1	44	7	2.652076697	6.40655E-08	8.81942E-07
TR22951-c0_g1	50	8	2.64385619	8.70729E-09	1.3409E-07
TR24126-c0_g4	25	4	2.64385619	4.72616E-05	0.000401755
TR4288-c0_g1	25	4	2.64385619	4.72616E-05	0.000401755
TR14955-c0_g1	131	21	2.641105579	1.29214E-20	4.78608E-19
TR24332-c0_g2	31	5	2.632268215	6.1878E-06	6.28815E-05
TR12181-c0_g1	37	6	2.624490865	8.22736E-07	9.66662E-06
TR19926-c0_g1	37	6	2.624490865	8.22736E-07	9.66662E-06
TR3112-c0_g1	37	6	2.624490865	8.22736E-07	9.66662E-06
TR14819-c0_g1	49	8	2.614709844	1.49964E-08	2.23256E-07
TR20289-c0_g1	120	20	2.584962501	1.32114E-18	4.36139E-17
TR11118-c0_g1	84	14	2.584962501	1.76089E-13	4.29519E-12
TR15031-c0_g1	48	8	2.584962501	2.57753E-08	3.74862E-07
TR8163-c0_g1	48	8	2.584962501	2.57753E-08	3.74862E-07
TR18042-c0_g1	36	6	2.584962501	1.4215E-06	1.5952E-05
TR18067-c0_g1	36	6	2.584962501	1.4215E-06	1.5952E-05
TR329-c0_g1	36	6	2.584962501	1.4215E-06	1.5952E-05
TR6813-c0_g1	36	6	2.584962501	1.4215E-06	1.5952E-05
TR23971-c0_g1	30	5	2.584962501	1.07345E-05	0.000104585
TR9289-c0_g1	30	5	2.584962501	1.07345E-05	0.000104585
TR13645-c0_g1	24	4	2.584962501	8.24713E-05	0.000666091
TR19035-c0_g2	24	4	2.584962501	8.24713E-05	0.000666091
TR25313-c0_g1	24	4	2.584962501	8.24713E-05	0.000666091
TR4832-c0_g1	24	4	2.584962501	8.24713E-05	0.000666091
TR11924-c0_g1	18	3	2.584962501	0.000650525	0.004168785
TR14517-c0_g1	18	3	2.584962501	0.000650525	0.004168785
TR14616-c0_g1	18	3	2.584962501	0.000650525	0.004168785
TR14740-c0_g1	18	3	2.584962501	0.000650525	0.004168785
TR14851-c0_g1	18	3	2.584962501	0.000650525	0.004168785

TR16945-c0_g1	18	3	2.584962501	0.000650525	0.004168785
TR21232-c1_g1	18	3	2.584962501	0.000650525	0.004168785
TR21984-c0_g1	18	3	2.584962501	0.000650525	0.004168785
TR23616-c0_g1	18	3	2.584962501	0.000650525	0.004168785
TR27350-c0_g1	18	3	2.584962501	0.000650525	0.004168785
TR3607-c0_g2	18	3	2.584962501	0.000650525	0.004168785
TR5181-c0_g1	18	3	2.584962501	0.000650525	0.004168785
TR8033-c0_g1	18	3	2.584962501	0.000650525	0.004168785
TR8264-c0_g3	18	3	2.584962501	0.000650525	0.004168785
TR8766-c0_g1	18	3	2.584962501	0.000650525	0.004168785
TR27329-c0_g1	71	12	2.564784619	1.56072E-11	3.22409E-10
TR19883-c0_g1	47	8	2.554588852	4.4207E-08	6.25273E-07
TR4072-c0_g1	129	22	2.551795637	1.27971E-19	4.4952E-18
TR11250-c0_g1	41	7	2.550197083	3.27468E-07	4.11485E-06
TR7253-c0_g1	41	7	2.550197083	3.27468E-07	4.11485E-06
TR3454-c0_g1	76	13	2.547487795	3.68092E-12	8.12733E-11
TR8513-c0_g1	76	13	2.547487795	3.68092E-12	8.12733E-11
TR13673-c0_g1	29	5	2.5360529	1.85649E-05	0.000173422
TR1458-c0_g1	29	5	2.5360529	1.85649E-05	0.000173422
TR1515-c0_g1	29	5	2.5360529	1.85649E-05	0.000173422
TR18160-c0_g1	29	5	2.5360529	1.85649E-05	0.000173422
TR5272-c0_g1	29	5	2.5360529	1.85649E-05	0.000173422
TR2946-c0_g1	69	12	2.523561956	4.52559E-11	8.95705E-10
TR12317-c0_g1	23	4	2.523561956	0.0001434	0.001092289
TR19035-c0_g1	23	4	2.523561956	0.0001434	0.001092289
TR26980-c0_g1	23	4	2.523561956	0.0001434	0.001092289
TR27215-c0_g1	23	4	2.523561956	0.0001434	0.001092289
TR2982-c0_g1	23	4	2.523561956	0.0001434	0.001092289
TR16294-c0_g1	40	7	2.514573173	5.61365E-07	6.82197E-06
TR17979-c0_g1	119	21	2.502500341	7.46245E-18	2.3848E-16
TR13709-c0_g1	34	6	2.502500341	4.2087E-06	4.37636E-05
TR4938-c0_g1	34	6	2.502500341	4.2087E-06	4.37636E-05
TR4959-c0_g1	34	6	2.502500341	4.2087E-06	4.37636E-05
TR13343-c0_g1	17	3	2.502500341	0.001140981	0.006775478
TR1363-c0_g1	17	3	2.502500341	0.001140981	0.006775478
TR15915-c0_g1	17	3	2.502500341	0.001140981	0.006775478
TR18194-c0_g1	17	3	2.502500341	0.001140981	0.006775478
TR1990-c0_g1	17	3	2.502500341	0.001140981	0.006775478
TR20177-c0_g2	17	3	2.502500341	0.001140981	0.006775478
TR2109-c0_g2	17	3	2.502500341	0.001140981	0.006775478
TR24062-c0_g1	17	3	2.502500341	0.001140981	0.006775478
TR2428-c0_g1	17	3	2.502500341	0.001140981	0.006775478
TR24332-c0_g1	17	3	2.502500341	0.001140981	0.006775478
TR25734-c0_g2	17	3	2.502500341	0.001140981	0.006775478

TR3964-c0_g1	17	3	2.502500341	0.001140981	0.006775478
TR485-c0_g1	17	3	2.502500341	0.001140981	0.006775478
TR8365-c0_g1	17	3	2.502500341	0.001140981	0.006775478
TR4912-c0_g2	62	11	2.494764692	5.58015E-10	9.872E-09
TR10373-c3_g7	152	27	2.493040011	2.81269E-22	1.12516E-20
TR626-c0_g1	140	25	2.485426827	1.40989E-20	5.21086E-19
TR26401-c0_g1	84	15	2.485426827	5.87786E-13	1.36892E-11
TR15176-c0_g1	28	5	2.485426827	3.20015E-05	0.000285001
TR18851-c0_g1	28	5	2.485426827	3.20015E-05	0.000285001
TR22373-c0_g3	28	5	2.485426827	3.20015E-05	0.000285001
TR26915-c0_g1	28	5	2.485426827	3.20015E-05	0.000285001
TR3276-c0_g1	28	5	2.485426827	3.20015E-05	0.000285001
TR477-c0_g1	28	5	2.485426827	3.20015E-05	0.000285001
TR3474-c0_g1	162	29	2.481869008	1.59587E-23	6.73249E-22
TR15613-c0_g1	39	7	2.478047297	9.5978E-07	1.11764E-05
TR26197-c1_g1	39	7	2.478047297	9.5978E-07	1.11764E-05
TR1637-c0_g1	150	27	2.473931188	7.97349E-22	3.15255E-20
TR19904-c0_g1	144	26	2.469485283	5.6414E-21	2.14567E-19
TR3440-c0_g1	155	28	2.468769483	1.89665E-22	7.6775E-21
TR16398-c0_g1	44	8	2.459431619	2.19956E-07	2.81677E-06
TR6248-c0_g1	44	8	2.459431619	2.19956E-07	2.81677E-06
TR15474-c0_g1	33	6	2.459431619	7.20961E-06	7.25283E-05
TR1129-c0_g1	22	4	2.459431619	0.000248369	0.001784075
TR13969-c0_g1	22	4	2.459431619	0.000248369	0.001784075
TR16327-c0_g1	22	4	2.459431619	0.000248369	0.001784075
TR17527-c0_g1	22	4	2.459431619	0.000248369	0.001784075
TR2251-c1_g1	22	4	2.459431619	0.000248369	0.001784075
TR5934-c0_g1	22	4	2.459431619	0.000248369	0.001784075
TR3966-c0_g1	395	72	2.455783842	2.7969E-54	3.00956E-52
TR20361-c0_g1	1484	272	2.447812535	4.5883E-198	2.0002E-195
TR16136-c0_g1	60	11	2.447458977	1.6025E-09	2.68155E-08
TR10321-c4_g10	321	59	2.443786438	3.37061E-44	2.8229E-42
TR10321-c4_g12	54	10	2.432959407	1.16893E-08	1.76651E-07
TR13393-c0_g1	27	5	2.432959407	5.4967E-05	0.00046126
TR14465-c0_g1	43	8	2.426264755	3.73635E-07	4.66394E-06
TR19761-c0_g1	43	8	2.426264755	3.73635E-07	4.66394E-06
TR16168-c0_g1	64	12	2.415037499	6.28789E-10	1.10665E-08
TR27483-c0_g1	48	9	2.415037499	8.5728E-08	1.1595E-06
TR7545-c0_g1	48	9	2.415037499	8.5728E-08	1.1595E-06
TR27804-c0_g1	32	6	2.415037499	1.23104E-05	0.000118378
TR4959-c0_g2	32	6	2.415037499	1.23104E-05	0.000118378
TR10742-c0_g1	16	3	2.415037499	0.001991715	0.010888033
TR13688-c0_g1	16	3	2.415037499	0.001991715	0.010888033

TR17834-c0_g1	16	3	2.415037499	0.001991715	0.010888033
TR18442-c0_g1	16	3	2.415037499	0.001991715	0.010888033
TR19557-c0_g1	16	3	2.415037499	0.001991715	0.010888033
TR24976-c0_g1	16	3	2.415037499	0.001991715	0.010888033
TR25505-c0_g1	16	3	2.415037499	0.001991715	0.010888033
TR27609-c0_g1	16	3	2.415037499	0.001991715	0.010888033
TR3573-c0_g1	16	3	2.415037499	0.001991715	0.010888033
TR5608-c0_g1	16	3	2.415037499	0.001991715	0.010888033
TR7586-c0_g1	16	3	2.415037499	0.001991715	0.010888033
TR18847-c0_g1	1463	275	2.411426246	6.3299E-192	2.6904E-189
TR20314-c0_g1	74	14	2.402098444	3.41342E-11	6.86778E-10
TR13200-c0_g1	37	7	2.402098444	2.78176E-06	3.00467E-05
TR13810-c3_g3	37	7	2.402098444	2.78176E-06	3.00467E-05
TR13765-c0_g1	63	12	2.392317423	1.05837E-09	1.81754E-08
TR13727-c0_g1	42	8	2.392317423	6.32972E-07	7.65933E-06
TR14652-c0_g2	21	4	2.392317423	0.000428331	0.002917546
TR21311-c2_g2	21	4	2.392317423	0.000428331	0.002917546
TR3591-c0_g1	21	4	2.392317423	0.000428331	0.002917546
TR4176-c0_g1	21	4	2.392317423	0.000428331	0.002917546
TR21952-c0_g1	52	10	2.378511623	3.32901E-08	4.80455E-07
TR26394-c0_g1	26	5	2.378511623	9.40516E-05	0.00075141
TR26874-c0_g1	26	5	2.378511623	9.40516E-05	0.00075141
TR3538-c0_g1	26	5	2.378511623	9.40516E-05	0.00075141
TR25382-c0_g2	291	56	2.377520421	5.3094E-39	3.97651E-37
TR886-c0_g1	135	26	2.376375879	5.85255E-19	1.97423E-17
TR10321-c5_g6	259	50	2.372952098	7.9805E-35	5.51541E-33
TR17638-c0_g1	31	6	2.36923381	2.09475E-05	0.000193342
TR406-c0_g1	31	6	2.36923381	2.09475E-05	0.000193342
TR24235-c0_g1	797	155	2.362311509	6.4256E-103	1.3162E-100
TR10106-c3_g1	413	81	2.350147969	6.84225E-54	7.13665E-52
TR26149-c0_g1	56	11	2.347923303	1.2894E-08	1.93653E-07
TR10365-c1_g4	183	36	2.345774837	9.02281E-25	4.04749E-23
TR13814-c2_g5	620	122	2.345387068	8.40995E-80	1.36172E-77
TR10587-c0_g4	578	114	2.342035668	2.1412E-74	3.22152E-72
TR10321-c4_g13	166	33	2.330645312	1.86152E-22	7.55329E-21
TR15872-c0_g1	166	33	2.330645312	1.86152E-22	7.55329E-21
TR17611-c0_g1	375	75	2.321928095	2.06538E-48	1.92935E-46
TR20311-c0_g3	85	17	2.321928095	3.39818E-12	7.5324E-11
TR20641-c0_g1	65	13	2.321928095	1.15433E-09	1.9704E-08
TR146-c0_g1	40	8	2.321928095	1.80093E-06	2.00256E-05
TR26762-c0_g1	40	8	2.321928095	1.80093E-06	2.00256E-05
TR4938-c0_g2	35	7	2.321928095	7.96246E-06	7.91651E-05
TR8517-c3_g1	35	7	2.321928095	7.96246E-06	7.91651E-05
TR9376-c0_g1	35	7	2.321928095	7.96246E-06	7.91651E-05

TR10545-c0_g1	30	6	2.321928095	3.55138E-05	0.000314268
TR3591-c0_g2	30	6	2.321928095	3.55138E-05	0.000314268
TR578-c0_g1	30	6	2.321928095	3.55138E-05	0.000314268
TR8803-c0_g1	30	6	2.321928095	3.55138E-05	0.000314268
TR17454-c0_g1	25	5	2.321928095	0.000160261	0.001209342
TR4678-c0_g1	25	5	2.321928095	0.000160261	0.001209342
TR6808-c0_g1	25	5	2.321928095	0.000160261	0.001209342
TR6963-c0_g1	25	5	2.321928095	0.000160261	0.001209342
TR13503-c0_g1	20	4	2.321928095	0.000735202	0.004672677
TR15295-c1_g2	20	4	2.321928095	0.000735202	0.004672677
TR4080-c0_g1	20	4	2.321928095	0.000735202	0.004672677
TR5066-c0_g1	20	4	2.321928095	0.000735202	0.004672677
TR5945-c0_g1	20	4	2.321928095	0.000735202	0.004672677
TR681-c0_g1	20	4	2.321928095	0.000735202	0.004672677
TR10151-c0_g1	15	3	2.321928095	0.003458025	0.017261009
TR16502-c0_g1	15	3	2.321928095	0.003458025	0.017261009
TR18951-c0_g1	15	3	2.321928095	0.003458025	0.017261009
TR25176-c0_g2	15	3	2.321928095	0.003458025	0.017261009
TR25313-c0_g2	15	3	2.321928095	0.003458025	0.017261009
TR26584-c0_g1	15	3	2.321928095	0.003458025	0.017261009
TR8033-c0_g2	15	3	2.321928095	0.003458025	0.017261009
TR8171-c0_g1	15	3	2.321928095	0.003458025	0.017261009
TR610-c1_g1	184	37	2.31410859	1.61438E-24	7.12899E-23
TR10319-c3_g1	49	10	2.292781749	1.57097E-07	2.06888E-06
TR22908-c0_g1	44	9	2.289506617	6.87217E-07	8.21884E-06
TR13359-c3_g1	361	74	2.286401661	7.79573E-46	6.86724E-44
TR22065-c0_g1	439	90	2.286224033	2.37658E-55	2.67582E-53
TR13659-c0_g1	78	16	2.285402219	4.01981E-11	7.9839E-10
TR10293-c0_g3	107	22	2.282035368	1.09951E-14	2.90718E-13
TR24758-c0_g1	97	20	2.277984747	1.99386E-13	4.84261E-12
TR3161-c0_g1	58	12	2.273018494	1.38538E-08	2.06971E-07
TR14833-c0_g1	29	6	2.273018494	5.99729E-05	0.000500058
TR18785-c0_g1	29	6	2.273018494	5.99729E-05	0.000500058
TR24474-c0_g1	29	6	2.273018494	5.99729E-05	0.000500058
TR27114-c0_g1	193	40	2.270528942	4.36063E-25	1.98226E-23
TR3954-c3_g1	702	146	2.265502661	1.8145E-86	3.35315E-84
TR12317-c0_g2	24	5	2.263034406	0.000271856	0.001940348
TR16176-c0_g1	24	5	2.263034406	0.000271856	0.001940348
TR17371-c0_g1	24	5	2.263034406	0.000271856	0.001940348
TR20202-c0_g2	24	5	2.263034406	0.000271856	0.001940348
TR21563-c0_g1	24	5	2.263034406	0.000271856	0.001940348
TR22084-c0_g1	24	5	2.263034406	0.000271856	0.001940348
TR23665-c0_g3	24	5	2.263034406	0.000271856	0.001940348
TR3789-c1_g1	24	5	2.263034406	0.000271856	0.001940348

TR5199-c0_g1	91	19	2.259867127	1.40672E-12	3.2059E-11
TR15136-c0_g1	158	33	2.259386629	1.03292E-20	3.84266E-19
TR14757-c0_g1	43	9	2.256339753	1.14814E-06	1.32248E-05
TR4652-c0_g1	358	75	2.254997087	9.88893E-45	8.36442E-43
TR12837-c0_g1	57	12	2.247927513	2.30137E-08	3.37296E-07
TR1049-c0_g1	38	8	2.247927513	5.06013E-06	5.19498E-05
TR15605-c0_g1	38	8	2.247927513	5.06013E-06	5.19498E-05
TR26291-c0_g2	38	8	2.247927513	5.06013E-06	5.19498E-05
TR6132-c0_g1	38	8	2.247927513	5.06013E-06	5.19498E-05
TR6839-c0_g1	38	8	2.247927513	5.06013E-06	5.19498E-05
TR14652-c0_g1	19	4	2.247927513	0.001255357	0.007372276
TR15295-c1_g5	19	4	2.247927513	0.001255357	0.007372276
TR15332-c0_g1	19	4	2.247927513	0.001255357	0.007372276
TR22654-c0_g1	19	4	2.247927513	0.001255357	0.007372276
TR24899-c0_g1	19	4	2.247927513	0.001255357	0.007372276
TR26439-c0_g1	19	4	2.247927513	0.001255357	0.007372276
TR26442-c0_g1	19	4	2.247927513	0.001255357	0.007372276
TR27130-c0_g1	19	4	2.247927513	0.001255357	0.007372276
TR3568-c0_g1	19	4	2.247927513	0.001255357	0.007372276
TR6284-c0_g1	19	4	2.247927513	0.001255357	0.007372276
TR8484-c0_g1	19	4	2.247927513	0.001255357	0.007372276
TR9525-c0_g1	19	4	2.247927513	0.001255357	0.007372276
TR15551-c0_g1	33	7	2.237039197	2.24755E-05	0.000206213
TR14762-c2_g1	707	150	2.236747714	9.78206E-86	1.76923E-83
TR3766-c0_g1	61	13	2.230297619	8.80662E-09	1.35375E-07
TR10293-c0_g7	159	34	2.225420114	1.78259E-20	6.54564E-19
TR7519-c0_g1	28	6	2.222392421	0.000100853	0.000799735
TR8077-c0_g1	28	6	2.222392421	0.000100853	0.000799735
TR14687-c0_g1	107	23	2.21790503	3.14655E-14	8.06871E-13
TR10321-c4_g2	79	17	2.216317907	7.00293E-11	1.34988E-09
TR12600-c0_g1	784	169	2.213830408	1.09659E-93	2.16784E-91
TR20311-c0_g2	88	19	2.211504105	6.30045E-12	1.36628E-10
TR19884-c0_g1	125	27	2.210896782	2.61718E-16	7.72492E-15
TR7776-c0_g1	37	8	2.209453366	8.4386E-06	8.36057E-05
TR16522-c0_g1	60	13	2.206450877	1.45529E-08	2.17225E-07
TR15016-c0_g1	46	10	2.201633861	7.23444E-07	8.58305E-06
TR10371-c0_g1	23	5	2.201633861	0.000458919	0.003071373
TR11921-c0_g1	23	5	2.201633861	0.000458919	0.003071373
TR22384-c0_g2	23	5	2.201633861	0.000458919	0.003071373
TR25176-c0_g1	23	5	2.201633861	0.000458919	0.003071373
TR2636-c0_g1	23	5	2.201633861	0.000458919	0.003071373
TR5251-c0_g1	23	5	2.201633861	0.000458919	0.003071373
TR14491-c0_g1	55	12	2.196397213	6.30333E-08	8.70552E-07
TR13277-c0_g1	32	7	2.192645078	3.75436E-05	0.000330549

TR21729-c0_g1	41	9	2.187627003	3.17448E-06	3.40507E-05
TR8464-c0_g1	200	44	2.184424571	8.52959E-25	3.83636E-23
TR19859-c0_g1	199	44	2.177193002	1.39002E-24	6.17027E-23
TR8501-c0_g1	252	56	2.169925001	1.43243E-30	8.39768E-29
TR10318-c1_g14	207	46	2.169925001	2.09308E-25	9.69623E-24
TR15433-c0_g1	108	24	2.169925001	5.33596E-14	1.34398E-12
TR17356-c0_g1	81	18	2.169925001	7.24931E-11	1.39421E-09
TR26943-c0_g1	63	14	2.169925001	9.13756E-09	1.40208E-07
TR19409-c0_g1	54	12	2.169925001	1.0391E-07	1.3908E-06
TR17978-c0_g1	36	8	2.169925001	1.40214E-05	0.000133738
TR3908-c0_g1	36	8	2.169925001	1.40214E-05	0.000133738
TR13162-c0_g1	27	6	2.169925001	0.000168837	0.001268425
TR8093-c0_g1	27	6	2.169925001	0.000168837	0.001268425
TR13994-c0_g1	18	4	2.169925001	0.002131187	0.011448734
TR15321-c0_g1	18	4	2.169925001	0.002131187	0.011448734
TR19481-c0_g1	18	4	2.169925001	0.002131187	0.011448734
TR21578-c0_g2	18	4	2.169925001	0.002131187	0.011448734
TR23699-c0_g1	18	4	2.169925001	0.002131187	0.011448734
TR3999-c0_g1	18	4	2.169925001	0.002131187	0.011448734
TR7212-c0_g1	18	4	2.169925001	0.002131187	0.011448734
TR7740-c0_g1	18	4	2.169925001	0.002131187	0.011448734
TR9057-c0_g2	18	4	2.169925001	0.002131187	0.011448734
TR9300-c0_g1	18	4	2.169925001	0.002131187	0.011448734
TR11273-c0_g1	80	18	2.152003093	1.18619E-10	2.23953E-09
TR26456-c0_g1	40	9	2.152003093	5.25193E-06	5.37891E-05
TR14464-c0_g1	31	7	2.146841388	6.24534E-05	0.0005182
TR26399-c0_g1	31	7	2.146841388	6.24534E-05	0.0005182
TR27114-c0_g2	84	19	2.144389909	4.53997E-11	8.97506E-10
TR22934-c0_g1	128	29	2.142019005	4.53026E-16	1.31884E-14
TR5571-c0_g2	75	17	2.141355849	5.07144E-10	9.06637E-09
TR15942-c0_g1	110	25	2.137503524	5.45778E-14	1.36858E-12
TR24238-c0_g1	44	10	2.137503524	1.97234E-06	2.17319E-05
TR11829-c0_g1	22	5	2.137503524	0.000770618	0.00487408
TR20387-c0_g1	22	5	2.137503524	0.000770618	0.00487408
TR359-c0_g1	22	5	2.137503524	0.000770618	0.00487408
TR76-c0_g2	22	5	2.137503524	0.000770618	0.00487408
TR10785-c0_g2	57	13	2.132450296	6.4698E-08	8.88168E-07
TR13814-c2_g2	455	104	2.129283017	1.41891E-52	1.43591E-50
TR21333-c0_g1	35	8	2.129283017	2.32081E-05	0.000212019
TR4079-c0_g1	35	8	2.129283017	2.32081E-05	0.000212019
TR8905-c0_g1	35	8	2.129283017	2.32081E-05	0.000212019
TR3435-c0_g1	354	81	2.127755547	2.94942E-41	2.34318E-39
TR13672-c0_g1	61	14	2.123382416	2.45369E-08	3.57464E-07
TR22912-c0_g1	52	12	2.115477217	2.80039E-07	3.53718E-06

TR11034-c0_g1	39	9	2.115477217	8.65783E-06	8.53867E-05
TR15451-c0_g1	39	9	2.115477217	8.65783E-06	8.53867E-05
TR16851-c0_g1	39	9	2.115477217	8.65783E-06	8.53867E-05
TR11945-c0_g1	26	6	2.115477217	0.000281287	0.001998436
TR24242-c0_g1	26	6	2.115477217	0.000281287	0.001998436
TR12128-c0_g15	56	13	2.106915204	1.05825E-07	1.41215E-06
TR7524-c0_g1	43	10	2.10433666	3.24056E-06	3.4631E-05
TR19409-c0_g2	103	24	2.101538026	6.091E-13	1.41662E-11
TR10560-c0_g1	60	14	2.099535674	4.00563E-08	5.70361E-07
TR19465-c0_g2	30	7	2.099535674	0.000103433	0.000816385
TR13814-c2_g8	268	63	2.088809267	6.073E-31	3.59753E-29
TR8362-c0_g2	34	8	2.087462841	3.82578E-05	0.000335448
TR16720-c0_g2	17	4	2.087462841	0.003594985	0.017762134
TR20123-c0_g1	17	4	2.087462841	0.003594985	0.017762134
TR21368-c0_g1	17	4	2.087462841	0.003594985	0.017762134
TR21946-c0_g1	17	4	2.087462841	0.003594985	0.017762134
TR24366-c0_g1	17	4	2.087462841	0.003594985	0.017762134
TR24366-c0_g2	17	4	2.087462841	0.003594985	0.017762134
TR2702-c0_g1	17	4	2.087462841	0.003594985	0.017762134
TR3986-c0_g1	17	4	2.087462841	0.003594985	0.017762134
TR6464-c0_g1	17	4	2.087462841	0.003594985	0.017762134
TR8590-c0_g1	17	4	2.087462841	0.003594985	0.017762134
TR10785-c0_g1	55	13	2.080919995	1.72613E-07	2.2505E-06
TR471-c0_g1	604	143	2.078533403	3.84955E-67	5.11308E-65
TR27130-c0_g2	38	9	2.078002512	1.42189E-05	0.000135353
TR18843-c0_g1	59	14	2.075288127	6.52198E-08	8.92772E-07
TR24205-c0_g1	59	14	2.075288127	6.52198E-08	8.92772E-07
TR9482-c0_g1	59	14	2.075288127	6.52198E-08	8.92772E-07
TR6488-c0_g1	42	10	2.070389328	5.30569E-06	5.43069E-05
TR1307-c0_g2	21	5	2.070389328	0.001286609	0.007524612
TR13710-c0_g1	21	5	2.070389328	0.001286609	0.007524612
TR21729-c0_g2	21	5	2.070389328	0.001286609	0.007524612
TR25208-c0_g1	21	5	2.070389328	0.001286609	0.007524612
TR26981-c0_g1	21	5	2.070389328	0.001286609	0.007524612
TR3912-c0_g1	21	5	2.070389328	0.001286609	0.007524612
TR6454-c1_g1	595	142	2.066998739	1.05272E-65	1.37674E-63
TR24038-c0_g1	46	11	2.064130337	1.98644E-06	2.1859E-05
TR8068-c1_g2	46	11	2.064130337	1.98644E-06	2.1859E-05
TR5613-c0_g1	146	35	2.060541542	2.63614E-17	8.22347E-16
TR19694-c0_g1	100	24	2.058893689	2.57564E-12	5.75418E-11
TR28-c0_g2	25	6	2.058893689	0.000466213	0.003103462
TR2808-c0_g1	25	6	2.058893689	0.000466213	0.003103462
TR3904-c0_g1	25	6	2.058893689	0.000466213	0.003103462
TR916-c0_g1	25	6	2.058893689	0.000466213	0.003103462

TR23578-c0_g1	129	31	2.057030945	1.94377E-15	5.41748E-14
TR26930-c0_g1	83	20	2.053111336	1.9559E-10	3.62233E-09
TR1612-c0_g2	58	14	2.050626073	1.05903E-07	1.41215E-06
TR19465-c0_g1	29	7	2.050626073	0.000170498	0.001278646
TR26924-c0_g1	29	7	2.050626073	0.000170498	0.001278646
TR21978-c0_g1	124	30	2.047305715	8.14624E-15	2.18105E-13
TR27101-c0_g1	124	30	2.047305715	8.14624E-15	2.18105E-13
TR18783-c0_g1	293	71	2.045009735	8.3901E-33	5.3027E-31
TR3180-c0_g1	66	16	2.044394119	1.51525E-08	2.25382E-07
TR16672-c0_g1	37	9	2.039528364	2.32599E-05	0.000212378
TR10321-c4_g5	41	10	2.03562391	8.65517E-06	8.53867E-05
TR12118-c0_g2	41	10	2.03562391	8.65517E-06	8.53867E-05
TR8068-c0_g1	41	10	2.03562391	8.65517E-06	8.53867E-05
TR11155-c0_g1	86	21	2.033947332	1.20188E-10	2.26035E-09
TR26965-c0_g1	86	21	2.033947332	1.20188E-10	2.26035E-09
TR620-c0_g1	86	21	2.033947332	1.20188E-10	2.26035E-09
TR21423-c0_g5	135	33	2.032421478	7.39859E-16	2.11761E-14
TR10092-c0_g1	45	11	2.032421478	3.23298E-06	3.4631E-05
TR23993-c0_g1	45	11	2.032421478	3.23298E-06	3.4631E-05
TR15997-c0_g1	49	12	2.029747343	1.21152E-06	1.38238E-05
TR24205-c0_g3	57	14	2.025535092	1.71482E-07	2.23746E-06
TR13979-c0_g1	61	15	2.023846742	6.47267E-08	8.88168E-07
TR14779-c0_g1	61	15	2.023846742	6.47267E-08	8.88168E-07
TR19551-c4_g1	61	15	2.023846742	6.47267E-08	8.88168E-07
TR18728-c0_g1	65	16	2.022367813	2.44764E-08	3.56888E-07
TR2565-c0_g1	65	16	2.022367813	2.44764E-08	3.56888E-07
TR18770-c11_g1	2432	607	2.002374807	2.9266E-252	1.7157E-249
TR9463-c0_g1	68	17	2	1.49239E-08	2.22371E-07
TR1679-c0_g1	60	15	2	1.04388E-07	1.39414E-06
TR10445-c0_g1	56	14	2	2.76865E-07	3.50228E-06
TR16522-c0_g2	56	14	2	2.76865E-07	3.50228E-06
TR15640-c0_g1	52	13	2	7.3597E-07	8.70129E-06
TR15416-c1_g1	48	12	2	1.96144E-06	2.16258E-05
TR27509-c0_g1	44	11	2	5.24316E-06	5.37316E-05
TR16743-c0_g1	40	10	2	1.40653E-05	0.000133966
TR18316-c0_g1	36	9	2	3.78919E-05	0.000333271
TR27565-c0_g1	36	9	2	3.78919E-05	0.000333271
TR24217-c0_g1	32	8	2	0.000102608	0.000810577
TR24913-c0_g2	32	8	2	0.000102608	0.000810577
TR25703-c0_g1	32	8	2	0.000102608	0.000810577
TR4184-c0_g1	32	8	2	0.000102608	0.000810577
TR13396-c0_g1	24	6	2	0.000768423	0.00486744
TR1365-c0_g1	24	6	2	0.000768423	0.00486744
TR23572-c0_g1	24	6	2	0.000768423	0.00486744

TR25019-c0_g1	24	6	2	0.000768423	0.00486744
TR2636-c0_g2	24	6	2	0.000768423	0.00486744
TR560-c0_g1	24	6	2	0.000768423	0.00486744
TR76-c0_g1	24	6	2	0.000768423	0.00486744
TR12740-c0_g1	20	5	2	0.002134686	0.011448734
TR14904-c0_g1	20	5	2	0.002134686	0.011448734
TR19025-c0_g2	20	5	2	0.002134686	0.011448734
TR19609-c0_g1	20	5	2	0.002134686	0.011448734
TR25233-c0_g1	20	5	2	0.002134686	0.011448734
TR3209-c0_g1	20	5	2	0.002134686	0.011448734
TR3439-c0_g1	20	5	2	0.002134686	0.011448734
TR20834-c0_g1	63	16	1.977279923	6.33684E-08	8.7376E-07
TR6075-c0_g1	63	16	1.977279923	6.33684E-08	8.7376E-07
TR10682-c0_g2	55	14	1.974004791	4.45674E-07	5.47079E-06
TR12128-c0_g5	39	10	1.963474124	2.27661E-05	0.000208429
TR12269-c0_g1	39	10	1.963474124	2.27661E-05	0.000208429
TR5938-c0_g1	39	10	1.963474124	2.27661E-05	0.000208429
TR618-c6_g1	308	79	1.963005793	1.1375E-32	7.16262E-31
TR11210-c0_g2	2237	575	1.959931395	1.0685E-225	5.3431E-223
TR19449-c0_g2	284	73	1.959922561	3.4E-30	1.95285E-28
TR25790-c0_g2	35	9	1.959358016	6.14597E-05	0.000510702
TR26246-c0_g1	35	9	1.959358016	6.14597E-05	0.000510702
TR3858-c1_g1	690	178	1.954719121	1.23357E-70	1.74769E-68
TR16415-c0_g1	31	8	1.95419631	0.000166857	0.001254658
TR16485-c0_g1	31	8	1.95419631	0.000166857	0.001254658
TR3294-c0_g1	31	8	1.95419631	0.000166857	0.001254658
TR381-c0_g1	31	8	1.95419631	0.000166857	0.001254658
TR14687-c0_g2	58	15	1.9510904	2.69198E-07	3.41802E-06
TR6632-c0_g1	85	22	1.949959318	4.8251E-10	8.64417E-09
TR2214-c0_g1	27	7	1.94753258	0.000456224	0.003071373
TR2218-c0_g1	27	7	1.94753258	0.000456224	0.003071373
TR1612-c0_g1	50	13	1.943416472	1.90481E-06	2.10835E-05
TR24205-c0_g2	50	13	1.943416472	1.90481E-06	2.10835E-05
TR9645-c0_g2	276	72	1.938599455	5.4932E-29	3.02238E-27
TR19834-c0_g1	92	24	1.938599455	1.1145E-10	2.11238E-09
TR20791-c0_g2	69	18	1.938599455	2.31988E-08	3.39423E-07
TR20834-c0_g2	69	18	1.938599455	2.31988E-08	3.39423E-07
TR9575-c1_g1	46	12	1.938599455	5.08788E-06	5.22032E-05
TR22230-c0_g1	23	6	1.938599455	0.001258959	0.007383226
TR24073-c0_g1	23	6	1.938599455	0.001258959	0.007383226
TR26630-c0_g1	23	6	1.938599455	0.001258959	0.007383226
TR9324-c0_g1	23	6	1.938599455	0.001258959	0.007383226
TR8568-c0_g1	88	23	1.935869663	2.92401E-10	5.36268E-09
TR11210-c0_g1	2221	581	1.934599324	2.9962E-220	1.3767E-217

TR7250-c0_g1	61	16	1.930737338	1.62268E-07	2.12869E-06
TR10483-c0_g1	19	5	1.925999419	0.003517639	0.017502052
TR13678-c0_g1	19	5	1.925999419	0.003517639	0.017502052
TR15668-c0_g1	19	5	1.925999419	0.003517639	0.017502052
TR16133-c0_g1	19	5	1.925999419	0.003517639	0.017502052
TR21402-c1_g1	19	5	1.925999419	0.003517639	0.017502052
TR27253-c0_g2	19	5	1.925999419	0.003517639	0.017502052
TR27787-c0_g1	19	5	1.925999419	0.003517639	0.017502052
TR3945-c0_g1	19	5	1.925999419	0.003517639	0.017502052
TR4121-c0_g1	19	5	1.925999419	0.003517639	0.017502052
TR5734-c0_g1	19	5	1.925999419	0.003517639	0.017502052
TR8474-c1_g1	300	79	1.925037942	4.40457E-31	2.62749E-29
TR20748-c0_g1	102	27	1.91753784	1.56054E-11	3.22409E-10
TR21238-c0_g1	34	9	1.91753784	9.92292E-05	0.000787963
TR21971-c0_g1	34	9	1.91753784	9.92292E-05	0.000787963
TR1679-c0_g2	83	22	1.915607813	1.21935E-09	2.07514E-08
TR15672-c0_g1	64	17	1.912537159	9.76484E-08	1.31445E-06
TR8487-c0_g1	64	17	1.912537159	9.76484E-08	1.31445E-06
TR27395-c0_g2	79	21	1.911463325	3.20565E-09	5.18064E-08
TR9359-c0_g2	60	16	1.906890596	2.58546E-07	3.29507E-06
TR10373-c3_g5	206	55	1.905140814	1.43073E-21	5.56619E-20
TR10132-c0_g1	101	27	1.903323981	2.46859E-11	5.03834E-10
TR25078-c0_g2	41	11	1.898120386	2.18607E-05	0.000201552
TR9535-c0_g1	108	29	1.896906507	5.70466E-12	1.24183E-10
TR26906-c0_g1	186	50	1.895302621	1.67123E-19	5.82236E-18
TR15261-c0_g1	26	7	1.893084796	0.000740075	0.00470189
TR17635-c0_g1	85	23	1.88582898	1.16318E-09	1.98352E-08
TR18856-c0_g1	576	156	1.884522783	1.73328E-56	2.00463E-54
TR1962-c0_g1	48	13	1.884522783	4.86262E-06	5.02254E-05
TR26919-c0_g1	107	29	1.883485991	8.99435E-12	1.91864E-10
TR13321-c0_g1	1958	531	1.882596999	2.1649E-187	8.7634E-185
TR17931-c0_g1	44	12	1.874469118	1.30035E-05	0.000124551
TR21971-c0_g2	33	9	1.874469118	0.000159436	0.001205258
TR10293-c0_g11	22	6	1.874469118	0.002049337	0.011156634
TR22225-c0_g1	22	6	1.874469118	0.002049337	0.011156634
TR25659-c0_g1	22	6	1.874469118	0.002049337	0.011156634
TR26118-c0_g1	22	6	1.874469118	0.002049337	0.011156634
TR3639-c0_g1	22	6	1.874469118	0.002049337	0.011156634
TR4233-c0_g1	22	6	1.874469118	0.002049337	0.011156634
TR7374-c0_g1	22	6	1.874469118	0.002049337	0.011156634
TR9639-c0_g1	22	6	1.874469118	0.002049337	0.011156634
TR9825-c0_g1	22	6	1.874469118	0.002049337	0.011156634
TR3282-c0_g1	1812	495	1.872082525	3.0627E-172	1.1571E-169
TR20791-c0_g1	62	17	1.866733469	2.45662E-07	3.13793E-06

TR16522-c0_g3	51	14	1.86507042	2.89792E-06	3.12221E-05
TR21445-c0_g1	138	38	1.860596943	1.55901E-14	4.07772E-13
TR22988-c0_g1	69	19	1.860596943	5.56492E-08	7.78692E-07
TR25814-c0_g2	69	19	1.860596943	5.56492E-08	7.78692E-07
TR20347-c0_g1	58	16	1.857980995	6.50425E-07	7.84261E-06
TR26752-c0_g2	58	16	1.857980995	6.50425E-07	7.84261E-06
TR8568-c0_g2	58	16	1.857980995	6.50425E-07	7.84261E-06
TR253-c0_g1	29	8	1.857980995	0.000434416	0.002951899
TR10682-c0_g1	47	13	1.854149134	7.7268E-06	7.70022E-05
TR25078-c0_g1	47	13	1.854149134	7.7268E-06	7.70022E-05
TR25286-c0_g1	54	15	1.847996907	1.72551E-06	1.92494E-05
TR9645-c0_g1	93	26	1.838719093	3.9639E-10	7.17695E-09
TR12117-c0_g1	25	7	1.836501268	0.001193247	0.007066102
TR22552-c0_g2	25	7	1.836501268	0.001193247	0.007066102
TR280-c0_g1	25	7	1.836501268	0.001193247	0.007066102
TR19924-c0_g1	32	9	1.830074999	0.00025487	0.001826781
TR5901-c0_g1	32	9	1.830074999	0.00025487	0.001826781
TR6493-c0_g1	32	9	1.830074999	0.00025487	0.001826781
TR11146-c0_g1	78	22	1.8259706	1.18955E-08	1.79609E-07
TR10587-c0_g2	39	11	1.8259706	5.54697E-05	0.000464561
TR16835-c0_g1	39	11	1.8259706	5.54697E-05	0.000464561
TR26945-c0_g1	39	11	1.8259706	5.54697E-05	0.000464561
TR24882-c0_g1	46	13	1.823122238	1.22309E-05	0.000117967
TR6647-c0_g1	53	15	1.821029859	2.72293E-06	2.94488E-05
TR13746-c4_g2	2023	573	1.819889276	8.0221E-185	3.0997E-182
TR19449-c0_g1	127	36	1.818759685	4.01626E-13	9.50998E-12
TR18898-c0_g1	141	40	1.817623258	2.15257E-14	5.57873E-13
TR10318-c1_g17	133	38	1.807354922	1.4465E-13	3.54868E-12
TR8158-c0_g1	91	26	1.807354922	9.69726E-10	1.67888E-08
TR18850-c0_g1	84	24	1.807354922	4.24313E-09	6.76725E-08
TR20355-c0_g1	84	24	1.807354922	4.24313E-09	6.76725E-08
TR13291-c0_g1	77	22	1.807354922	1.86245E-08	2.751E-07
TR13363-c0_g1	56	16	1.807354922	1.61553E-06	1.80699E-05
TR24821-c0_g1	56	16	1.807354922	1.61553E-06	1.80699E-05
TR4089-c0_g1	49	14	1.807354922	7.23706E-06	7.27614E-05
TR6766-c0_g1	35	10	1.807354922	0.000149535	0.001136983
TR2222-c0_g1	28	8	1.807354922	0.000695075	0.004437817
TR6202-c0_g1	28	8	1.807354922	0.000695075	0.004437817
TR9157-c0_g1	21	6	1.807354922	0.003312672	0.016608611
TR8113-c0_g1	181	52	1.799406169	8.18097E-18	2.59491E-16
TR11138-c1_g1	90	26	1.791413378	1.51181E-09	2.54718E-08
TR26182-c0_g1	45	13	1.791413378	1.92835E-05	0.000179053
TR11144-c0_g1	38	11	1.788495895	8.77623E-05	0.000704141
TR18671-c0_g1	38	11	1.788495895	8.77623E-05	0.000704141

TR26125-c0_g1	38	11	1.788495895	8.77623E-05	0.000704141
TR1020-c0_g1	31	9	1.784271309	0.000405239	0.002781431
TR10519-c0_g1	31	9	1.784271309	0.000405239	0.002781431
TR15638-c0_g1	31	9	1.784271309	0.000405239	0.002781431
TR18310-c0_g1	31	9	1.784271309	0.000405239	0.002781431
TR18774-c0_g1	31	9	1.784271309	0.000405239	0.002781431
TR9527-c0_g1	55	16	1.781359714	2.5333E-06	2.74327E-05
TR349-c0_g1	48	14	1.777607579	1.13722E-05	0.000110481
TR21066-c0_g1	24	7	1.777607579	0.001911415	0.010512017
TR1298-c0_g2	82	24	1.772589504	1.03043E-08	1.56697E-07
TR21151-c0_g1	82	24	1.772589504	1.03043E-08	1.56697E-07
TR21833-c0_g1	41	12	1.772589504	5.1533E-05	0.000434157
TR9262-c0_g1	41	12	1.772589504	5.1533E-05	0.000434157
TR15971-c0_g1	34	10	1.765534746	0.000236511	0.001729146
TR9344-c0_g1	156	46	1.761840263	3.72599E-15	1.02669E-13
TR12567-c0_g1	61	18	1.760812336	8.87075E-07	1.03701E-05
TR17538-c0_g1	88	26	1.7589919	3.64973E-09	5.85933E-08
TR8349-c0_g1	44	13	1.7589919	3.02769E-05	0.000270777
TR488-c0_g1	179	53	1.755895323	4.32948E-17	1.32625E-15
TR18471-c0_g1	27	8	1.754887502	0.001105394	0.006636004
TR18683-c0_g1	27	8	1.754887502	0.001105394	0.006636004
TR24637-c0_g1	27	8	1.754887502	0.001105394	0.006636004
TR3431-c0_g1	27	8	1.754887502	0.001105394	0.006636004
TR3988-c0_g1	64	19	1.752072487	5.25062E-07	6.38994E-06
TR8811-c0_g1	64	19	1.752072487	5.25062E-07	6.38994E-06
TR10318-c1_g5	188	56	1.74723393	9.30316E-18	2.94536E-16
TR13656-c0_g1	94	28	1.74723393	1.29267E-09	2.18896E-08
TR10360-c0_g13	47	14	1.74723393	1.77994E-05	0.000166913
TR8068-c0_g2	47	14	1.74723393	1.77994E-05	0.000166913
TR15935-c0_g1	57	17	1.745427173	2.33672E-06	2.53686E-05
TR3724-c0_g2	77	23	1.743224585	4.17566E-08	5.91598E-07
TR7996-c0_g2	77	23	1.743224585	4.17566E-08	5.91598E-07
TR8075-c0_g1	294	88	1.740240726	9.91193E-27	4.89872E-25
TR3724-c0_g1	90	27	1.736965594	3.359E-09	5.41817E-08
TR15024-c0_g1	60	18	1.736965594	1.37988E-06	1.55362E-05
TR22910-c0_g1	30	9	1.736965594	0.00064067	0.004128975
TR9504-c3_g1	799	240	1.735161097	2.02745E-69	2.84871E-67
TR27395-c0_g1	73	22	1.73039294	1.09028E-07	1.45268E-06
TR15871-c0_g1	116	35	1.728697978	2.22621E-11	4.56007E-10
TR9473-c10_g1	1332	402	1.728326676	6.969E-114	1.6011E-111
TR8784-c0_g1	53	16	1.727920455	6.16326E-06	6.26696E-05
TR25121-c0_g1	96	29	1.726981506	1.18706E-09	2.02221E-08
TR23607-c0_g1	86	26	1.725825037	8.72947E-09	1.3431E-07
TR17479-c0_g2	43	13	1.725825037	4.73329E-05	0.000402135

TR10318-c1_g4	284	86	1.723482365	1.51686E-25	7.06539E-24
TR12916-c0_g1	33	10	1.722466024	0.000372084	0.002565258
TR13718-c0_g1	33	10	1.722466024	0.000372084	0.002565258
TR17958-c0_g1	33	10	1.722466024	0.000372084	0.002565258
TR6501-c0_g1	155	47	1.721535554	1.2484E-14	3.29571E-13
TR10360-c0_g5	122	37	1.721283972	7.90499E-12	1.70336E-10
TR960-c9_g1	1332	404	1.721166884	3.1811E-113	7.1162E-111
TR1124-c0_g1	23	7	1.716207034	0.003040506	0.015332054
TR24394-c0_g1	23	7	1.716207034	0.003040506	0.015332054
TR25044-c0_g2	23	7	1.716207034	0.003040506	0.015332054
TR6491-c0_g1	23	7	1.716207034	0.003040506	0.015332054
TR6745-c0_g2	23	7	1.716207034	0.003040506	0.015332054
TR8167-c0_g1	23	7	1.716207034	0.003040506	0.015332054
TR20328-c0_g1	108	33	1.710493383	1.48443E-10	2.77637E-09
TR7734-c0_g1	36	11	1.710493383	0.000216503	0.001597584
TR3752-c0_g1	85	26	1.708951218	1.34519E-08	2.01498E-07
TR22003-c0_g1	49	15	1.707819249	1.6279E-05	0.000153187
TR10891-c0_g1	75	23	1.705256734	9.96494E-08	1.33927E-06
TR4867-c2_g1	91	28	1.700439718	4.73194E-09	7.4976E-08
TR15025-c1_g1	65	20	1.700439718	7.44253E-07	8.79311E-06
TR14539-c0_g1	39	12	1.700439718	0.000126191	0.000979639
TR21125-c0_g1	39	12	1.700439718	0.000126191	0.000979639
TR12707-c0_g2	26	8	1.700439718	0.001746598	0.009735893
TR25119-c0_g1	26	8	1.700439718	0.001746598	0.009735893
TR496-c2_g1	1953	601	1.700255053	2.3834E-162	8.6214E-160
TR14997-c2_g1	1885	582	1.695473465	3.9084E-156	1.2778E-153
TR7996-c0_g3	68	21	1.695145418	4.39326E-07	5.40458E-06
TR8688-c0_g1	84	26	1.691877705	2.0678E-08	3.04638E-07
TR3483-c0_g1	71	22	1.690315501	2.59459E-07	3.30423E-06
TR18814-c0_g1	100	31	1.689659879	9.88933E-10	1.70826E-08
TR14566-c0_g1	58	18	1.688055994	3.30533E-06	3.52983E-05
TR1594-c0_g2	29	9	1.688055994	0.001006804	0.006116087
TR4650-c0_g1	45	14	1.684498174	4.3064E-05	0.000371082
TR5978-c0_g1	45	14	1.684498174	4.3064E-05	0.000371082
TR8996-c0_g1	45	14	1.684498174	4.3064E-05	0.000371082
TR15448-c0_g3	61	19	1.682809824	1.94574E-06	2.14871E-05
TR5973-c0_g1	61	19	1.682809824	1.94574E-06	2.14871E-05
TR525-c5_g1	443	138	1.682638432	1.16965E-37	8.53461E-36
TR4104-c0_g1	77	24	1.68182404	9.06217E-08	1.22374E-06
TR18252-c0_g1	80	25	1.678071905	5.35915E-08	7.52375E-07
TR19857-c0_g1	80	25	1.678071905	5.35915E-08	7.52375E-07
TR13039-c0_g1	32	10	1.678071905	0.000582093	0.00379316
TR13833-c0_g1	32	10	1.678071905	0.000582093	0.00379316
TR14522-c0_g1	32	10	1.678071905	0.000582093	0.00379316

TR17931-c0_g2	32	10	1.678071905	0.000582093	0.00379316
TR22221-c0_g1	32	10	1.678071905	0.000582093	0.00379316
TR6075-c0_g2	32	10	1.678071905	0.000582093	0.00379316
TR10141-c0_g1	99	31	1.67516031	1.51321E-09	2.54718E-08
TR27706-c0_g1	70	22	1.669851398	3.98502E-07	4.93449E-06
TR27395-c0_g3	89	28	1.668378509	1.11098E-08	1.68343E-07
TR12118-c0_g4	143	45	1.66801824	4.46979E-13	1.05107E-11
TR10587-c0_g1	1083	342	1.662965013	4.8582E-88	9.17732E-86
TR10588-c0_g1	38	12	1.662965013	0.000196028	0.001457252
TR13668-c0_g1	218	69	1.659659868	5.20795E-19	1.76379E-17
TR24296-c0_g1	82	26	1.657112286	4.84889E-08	6.82995E-07
TR25121-c0_g2	82	26	1.657112286	4.84889E-08	6.82995E-07
TR13975-c0_g1	41	13	1.657112286	0.000114113	0.000896525
TR899-c0_g1	249	79	1.656221184	1.98311E-21	7.64523E-20
TR4894-c0_g1	66	21	1.652076697	1.0358E-06	1.19877E-05
TR309-c0_g1	44	14	1.652076697	6.6546E-05	0.000548144
TR10489-c0_g1	22	7	1.652076697	0.004800413	0.023074179
TR15262-c0_g1	22	7	1.652076697	0.004800413	0.023074179
TR17600-c0_g1	22	7	1.652076697	0.004800413	0.023074179
TR22333-c0_g1	22	7	1.652076697	0.004800413	0.023074179
TR25044-c0_g1	22	7	1.652076697	0.004800413	0.023074179
TR25211-c0_g1	22	7	1.652076697	0.004800413	0.023074179
TR26291-c0_g1	22	7	1.652076697	0.004800413	0.023074179
TR12589-c0_g1	279	89	1.648387881	1.14951E-23	4.89806E-22
TR10366-c5_g5	47	15	1.647698256	3.88678E-05	0.000340095
TR16471-c0_g1	47	15	1.647698256	3.88678E-05	0.000340095
TR24279-c0_g1	75	24	1.64385619	2.12197E-07	2.74345E-06
TR9318-c0_g1	50	16	1.64385619	2.27338E-05	0.000208429
TR11340-c0_g1	25	8	1.64385619	0.00274079	0.014421866
TR25234-c0_g1	25	8	1.64385619	0.00274079	0.014421866
TR25475-c0_g2	25	8	1.64385619	0.00274079	0.014421866
TR13150-c0_g1	28	9	1.637429921	0.001572135	0.009011588
TR20372-c0_g1	28	9	1.637429921	0.001572135	0.009011588
TR25790-c0_g1	28	9	1.637429921	0.001572135	0.009011588
TR7996-c0_g1	87	28	1.635588574	2.58318E-08	3.75363E-07
TR12567-c0_g2	59	19	1.634715536	4.58182E-06	4.74114E-05
TR12567-c0_g3	59	19	1.634715536	4.58182E-06	4.74114E-05
TR15448-c0_g1	59	19	1.634715536	4.58182E-06	4.74114E-05
TR26371-c0_g1	31	10	1.632268215	0.000905263	0.005542193
TR3490-c0_g1	31	10	1.632268215	0.000905263	0.005542193
TR8068-c1_g1	31	10	1.632268215	0.000905263	0.005542193
TR11474-c0_g2	34	11	1.628031223	0.000522959	0.003441212
TR631-c0_g1	284	92	1.626185163	1.27043E-23	5.38629E-22
TR10373-c3_g1	108	35	1.625604485	6.58865E-10	1.15719E-08

TR13741-c0_g1	111	36	1.624490865	3.90631E-10	7.08022E-09
TR8491-c0_g1	37	12	1.624490865	0.000302943	0.002143337
TR581-c0_g1	234	76	1.622437206	1.1064E-19	3.90256E-18
TR15571-c0_g1	40	13	1.621488377	0.000175911	0.001318082
TR3758-c0_g1	83	27	1.620151929	6.64293E-08	9.08597E-07
TR10293-c0_g6	86	28	1.618909833	3.92416E-08	5.59697E-07
TR17541-c0_g1	43	14	1.618909833	0.000102361	0.00081018
TR3208-c0_g1	43	14	1.618909833	0.000102361	0.00081018
TR9202-c0_g1	43	14	1.618909833	0.000102361	0.00081018
TR25309-c0_g1	46	15	1.61667136	5.96719E-05	0.000498281
TR27754-c0_g1	46	15	1.61667136	5.96719E-05	0.000498281
TR15448-c0_g2	58	19	1.610053482	6.99353E-06	7.03963E-05
TR5887-c0_g3	61	20	1.608809243	4.10415E-06	4.28862E-05
TR10293-c0_g15	140	46	1.605721061	3.20625E-12	7.11625E-11
TR13379-c0_g1	188	62	1.600392541	7.91012E-16	2.25264E-14
TR21080-c1_g1	103	34	1.599037686	2.5432E-09	4.15349E-08
TR10299-c2_g3	745	246	1.59858211	8.27158E-58	9.69847E-56
TR9359-c0_g1	115	38	1.597562538	3.13462E-10	5.71197E-09
TR25382-c0_g1	497	165	1.590779827	7.07488E-39	5.25251E-37
TR6570-c0_g1	108	36	1.584962501	1.34432E-09	2.27415E-08
TR10562-c0_g1	96	32	1.584962501	1.09522E-08	1.66103E-07
TR23337-c0_g1	66	22	1.584962501	2.14996E-06	2.3476E-05
TR14787-c0_g1	45	15	1.584962501	9.11987E-05	0.000730334
TR16599-c0_g1	45	15	1.584962501	9.11987E-05	0.000730334
TR2745-c0_g1	42	14	1.584962501	0.0001567	0.001185629
TR27467-c0_g1	42	14	1.584962501	0.0001567	0.001185629
TR10588-c0_g2	39	13	1.584962501	0.000269804	0.001933004
TR15907-c0_g1	36	12	1.584962501	0.000465645	0.003103462
TR10830-c0_g1	33	11	1.584962501	0.00080585	0.00504623
TR11474-c0_g1	33	11	1.584962501	0.00080585	0.00504623
TR12962-c0_g1	33	11	1.584962501	0.00080585	0.00504623
TR15968-c0_g1	33	11	1.584962501	0.00080585	0.00504623
TR14724-c0_g2	30	10	1.584962501	0.001399101	0.008139916
TR483-c0_g1	30	10	1.584962501	0.001399101	0.008139916
TR9337-c0_g1	30	10	1.584962501	0.001399101	0.008139916
TR10591-c0_g1	27	9	1.584962501	0.002438394	0.012963077
TR5131-c0_g1	27	9	1.584962501	0.002438394	0.012963077
TR7054-c0_g1	27	9	1.584962501	0.002438394	0.012963077
TR9182-c0_g1	27	9	1.584962501	0.002438394	0.012963077
TR1190-c0_g1	24	8	1.584962501	0.004269387	0.020691343
TR21232-c0_g1	629	211	1.575817018	4.30113E-48	3.97418E-46
TR15980-c1_g1	333	112	1.572023445	3.63197E-26	1.75921E-24
TR8037-c0_g2	95	32	1.569855608	1.6484E-08	2.44332E-07
TR10098-c0_g1	68	23	1.563900885	1.90992E-06	2.11263E-05

TR2665-c0_g1	53	18	1.557995453	2.75375E-05	0.000248896
TR12128-c0_g9	50	17	1.556393349	4.71308E-05	0.000401044
TR27784-c0_g1	44	15	1.552541023	0.000138731	0.001060527
TR9242-c0_g1	44	15	1.552541023	0.000138731	0.001060527
TR15009-c0_g1	82	28	1.550197083	2.03561E-07	2.63379E-06
TR18220-c0_g1	79	27	1.548893246	3.4522E-07	4.32335E-06
TR18749-c0_g1	114	39	1.547487795	9.4325E-10	1.63638E-08
TR26896-c0_g1	38	13	1.547487795	0.000411626	0.002819846
TR12836-c0_g1	321	110	1.545069774	1.10604E-24	4.94848E-23
TR11167-c0_g1	70	24	1.544320516	1.69084E-06	1.8875E-05
TR17232-c0_g1	35	12	1.544320516	0.000711689	0.004533404
TR18844-c0_g1	32	11	1.540568381	0.001234235	0.007278403
TR25814-c0_g1	32	11	1.540568381	0.001234235	0.007278403
TR10321-c5_g5	151	52	1.537965021	2.38588E-12	5.34427E-11
TR13019-c0_g1	29	10	1.5360529	0.002148158	0.011510093
TR17931-c0_g3	29	10	1.5360529	0.002148158	0.011510093
TR24165-c0_g1	29	10	1.5360529	0.002148158	0.011510093
TR26529-c0_g1	52	18	1.530514717	4.15826E-05	0.000359592
TR10109-c0_g1	26	9	1.530514717	0.003754985	0.018493544
TR17209-c0_g2	26	9	1.530514717	0.003754985	0.018493544
TR25119-c0_g2	26	9	1.530514717	0.003754985	0.018493544
TR11116-c0_g1	75	26	1.528378972	8.78497E-07	1.02862E-05
TR1347-c0_g1	49	17	1.527247003	7.12219E-05	0.000584216
TR3261-c0_g1	46	16	1.523561956	0.000122188	0.000952043
TR1858-c0_g1	66	23	1.520832163	4.31618E-06	4.48538E-05
TR2580-c0_g1	63	22	1.517848305	7.35048E-06	7.38145E-05
TR10321-c4_g11	123	43	1.516249751	3.85912E-10	7.00214E-09
TR21998-c0_g1	80	28	1.514573173	4.56017E-07	5.58969E-06
TR26434-c0_g1	60	21	1.514573173	1.25306E-05	0.00012036
TR10373-c3_g6	277	97	1.513829324	6.40281E-21	2.41366E-19
TR10318-c1_g2	208	73	1.510615159	4.75118E-16	1.37844E-14
TR11823-c0_g1	187	66	1.502500341	1.77674E-14	4.62588E-13
TR24044-c0_g1	34	12	1.502500341	0.001081311	0.006505208
TR19958-c0_g1	48	17	1.497499659	0.000107145	0.000844511
TR10830-c0_g2	31	11	1.494764692	0.001878293	0.010374743
TR23091-c0_g1	31	11	1.494764692	0.001878293	0.010374743
TR26233-c0_g3	31	11	1.494764692	0.001878293	0.010374743
TR6177-c0_g1	183	65	1.493332025	4.40256E-14	1.11882E-12
TR11134-c0_g1	121	43	1.492598483	8.48615E-10	1.48127E-08
TR9586-c0_g2	45	16	1.491853096	0.000183922	0.001372058
TR11232-c0_g1	42	15	1.485426827	0.000316322	0.002230565
TR11892-c0_g1	28	10	1.485426827	0.003275405	0.016441165
TR17232-c0_g2	28	10	1.485426827	0.003275405	0.016441165
TR23976-c0_g1	28	10	1.485426827	0.003275405	0.016441165

TR7054-c0_g2	28	10	1.485426827	0.003275405	0.016441165
TR10293-c0_g4	162	58	1.481869008	1.65385E-12	3.74903E-11
TR4682-c0_g1	50	18	1.473931188	9.36012E-05	0.000748868
TR16200-c0_g1	36	13	1.469485283	0.000942129	0.005738959
TR25212-c0_g1	36	13	1.469485283	0.000942129	0.005738959
TR2665-c0_g2	47	17	1.46712601	0.000160441	0.001210164
TR13189-c0_g1	80	29	1.4639471	8.82491E-07	1.03259E-05
TR22296-c0_g1	55	20	1.459431619	4.76889E-05	0.00040478
TR11255-c0_g1	44	16	1.459431619	0.000275486	0.001963787
TR25575-c0_g1	44	16	1.459431619	0.000275486	0.001963787
TR1270-c0_g2	33	12	1.459431619	0.001632725	0.009206795
TR19772-c0_g1	33	12	1.459431619	0.001632725	0.009206795
TR26740-c0_g1	33	12	1.459431619	0.001632725	0.009206795
TR15963-c0_g1	52	19	1.452512205	8.15043E-05	0.000659848
TR14634-c0_g1	41	15	1.450661409	0.000473951	0.003151266
TR323-c0_g1	41	15	1.450661409	0.000473951	0.003151266
TR12688-c0_g1	30	11	1.447458977	0.002839259	0.014784427
TR15962-c0_g1	30	11	1.447458977	0.002839259	0.014784427
TR18238-c0_g1	30	11	1.447458977	0.002839259	0.014784427
TR18409-c0_g1	30	11	1.447458977	0.002839259	0.014784427
TR26914-c0_g1	30	11	1.447458977	0.002839259	0.014784427
TR27792-c0_g1	30	11	1.447458977	0.002839259	0.014784427
TR7310-c0_g1	30	11	1.447458977	0.002839259	0.014784427
TR1601-c0_g1	38	14	1.440572591	0.000817208	0.005109828
TR8529-c0_g1	38	14	1.440572591	0.000817208	0.005109828
TR19701-c0_g1	65	24	1.437405312	1.24235E-05	0.000119398
TR2055-c0_g2	73	27	1.434937057	3.74449E-06	3.94431E-05
TR16209-c0_g1	27	10	1.432959407	0.004957615	0.023749229
TR27481-c1_g1	27	10	1.432959407	0.004957615	0.023749229
TR2793-c0_g1	27	10	1.432959407	0.004957615	0.023749229
TR4210-c0_g1	27	10	1.432959407	0.004957615	0.023749229
TR547-c0_g1	143	53	1.431950882	1.02488E-10	1.95779E-09
TR7125-c0_g1	70	26	1.428843299	6.35205E-06	6.45121E-05
TR18016-c0_g1	35	13	1.428843299	0.00141268	0.008210635
TR1601-c0_g2	43	16	1.426264755	0.000410532	0.002815487
TR24339-c0_g1	51	19	1.424497829	0.000120926	0.000942641
TR11723-c0_g2	59	22	1.423211431	3.59735E-05	0.000317713
TR15930-c0_g1	59	22	1.423211431	3.59735E-05	0.000317713
TR13821-c0_g1	225	84	1.421463768	7.50175E-16	2.13993E-14
TR8134-c0_g1	211	79	1.417318441	6.71786E-15	1.82157E-13
TR26353-c0_g1	56	21	1.415037499	6.13012E-05	0.000510133
TR4203-c0_g1	56	21	1.415037499	6.13012E-05	0.000510133
TR10591-c0_g2	32	12	1.415037499	0.002449324	0.013000856
TR17891-c0_g1	32	12	1.415037499	0.002449324	0.013000856

TR2602-c0_g1	32	12	1.415037499	0.002449324	0.013000856
TR4110-c0_g1	32	12	1.415037499	0.002449324	0.013000856
TR7395-c0_g1	32	12	1.415037499	0.002449324	0.013000856
TR11823-c0_g2	93	35	1.409875794	2.60013E-07	3.30881E-06
TR5642-c0_g1	53	20	1.40599236	0.000104572	0.000824996
TR1231-c0_g1	45	17	1.404390255	0.00035454	0.00247136
TR13307-c0_g1	37	14	1.402098444	0.001217733	0.007198123
TR16559-c0_g1	37	14	1.402098444	0.001217733	0.007198123
TR7578-c0_g1	37	14	1.402098444	0.001217733	0.007198123
TR277-c0_g1	66	25	1.40053793	1.58477E-05	0.000149436
TR4027-c0_g1	153	58	1.399406848	5.08505E-11	9.99453E-10
TR10481-c0_g1	58	22	1.398549376	5.29842E-05	0.00044572
TR2559-c0_g1	29	11	1.398549376	0.004261552	0.020665151
TR5601-c0_g1	29	11	1.398549376	0.004261552	0.020665151
TR15999-c0_g1	50	19	1.395928676	0.000178596	0.001332908
TR17698-c0_g1	42	16	1.392317423	0.000608545	0.003949748
TR10869-c0_g1	118	45	1.390789953	9.4189E-09	1.44395E-07
TR3794-c0_g1	55	21	1.389042291	9.02348E-05	0.000723638
TR7523-c0_g1	123	47	1.387925654	4.85917E-09	7.69202E-08
TR24339-c0_g2	47	18	1.38466385	0.000305412	0.002155419
TR13628-c0_g1	219	84	1.382469637	6.96322E-15	1.8821E-13
TR7125-c0_g2	73	28	1.382469637	6.97631E-06	7.02645E-05
TR15923-c1_g1	44	17	1.371968777	0.000523023	0.003441212
TR921-c0_g1	44	17	1.371968777	0.000523023	0.003441212
TR11723-c0_g1	62	24	1.36923381	3.93666E-05	0.000344105
TR25894-c0_g1	62	24	1.36923381	3.93666E-05	0.000344105
TR14531-c0_g1	31	12	1.36923381	0.003649302	0.017999121
TR14981-c0_g1	31	12	1.36923381	0.003649302	0.017999121
TR18257-c0_g1	31	12	1.36923381	0.003649302	0.017999121
TR23634-c0_g1	31	12	1.36923381	0.003649302	0.017999121
TR27763-c0_g1	31	12	1.36923381	0.003649302	0.017999121
TR2612-c0_g1	80	31	1.367731785	3.07067E-06	3.29788E-05
TR18863-c0_g1	149	58	1.361187525	2.2325E-10	4.11664E-09
TR26888-c0_g1	1911	744	1.360953252	1.8264E-114	4.2537E-112
TR22938-c0_g1	554	216	1.358854664	2.43329E-34	1.64163E-32
TR13336-c0_g1	223	87	1.357956404	9.36653E-15	2.48818E-13
TR1347-c0_g2	46	18	1.353636955	0.000448568	0.003046843
TR20335-c0_g1	212	83	1.352881023	4.99601E-14	1.26397E-12
TR13684-c0_g1	240	94	1.352301744	1.13369E-15	3.18583E-14
TR313-c0_g1	51	20	1.350497247	0.000225246	0.001649926
TR10293-c0_g2	698	274	1.349051143	2.30602E-42	1.84931E-40
TR1298-c0_g1	33	13	1.343954401	0.003115819	0.015681776
TR21295-c0_g1	33	13	1.343954401	0.003115819	0.015681776
TR7771-c0_g1	38	15	1.341036918	0.001541529	0.008848086

TR15923-c1_g2	43	17	1.338801913	0.000767464	0.00486744
TR14783-c0_g1	58	23	1.334419039	9.72863E-05	0.00077398
TR13814-c2_g15	247	98	1.333657387	9.00019E-16	2.55451E-14
TR361-c0_g1	78	31	1.331205908	6.45081E-06	6.54369E-05
TR18512-c0_g1	113	45	1.328325866	5.94288E-08	8.26139E-07
TR24091-c0_g1	50	20	1.321928095	0.000328258	0.002301372
TR25442-c0_g1	45	18	1.321928095	0.000655418	0.004198559
TR15493-c0_g1	40	16	1.321928095	0.00131499	0.007685312
TR24251-c0_g1	35	14	1.321928095	0.002654181	0.014013849
TR13695-c0_g1	77	31	1.31259023	9.30431E-06	9.15426E-05
TR10318-c1_g1	62	25	1.310340121	7.12078E-05	0.000584216
TR4703-c0_g1	57	23	1.309328058	0.000141033	0.001077153
TR23196-c0_g1	52	21	1.308122295	0.000280225	0.001996728
TR11777-c0_g1	47	19	1.306661338	0.00055892	0.003661801
TR25516-c0_g1	42	17	1.304854582	0.001119929	0.006716145
TR13448-c0_g1	74	30	1.30256277	1.56494E-05	0.000147647
TR13428-c0_g1	32	13	1.299560282	0.004580418	0.022116774
TR1497-c0_g2	86	35	1.296981738	3.4726E-06	3.69454E-05
TR10091-c0_g1	76	31	1.293731203	1.33752E-05	0.000127967
TR22015-c0_g1	66	27	1.289506617	5.19153E-05	0.000437162
TR11172-c0_g1	61	25	1.286881148	0.000102649	0.000810577
TR2055-c0_g1	102	42	1.280107919	5.67673E-07	6.88879E-06
TR10293-c0_g5	97	40	1.277984747	1.10847E-06	1.27766E-05
TR11887-c0_g1	41	17	1.270089163	0.001624909	0.009180994
TR15215-c0_g1	53	22	1.268488836	0.000344313	0.002405002
TR15480-c0_g1	53	22	1.268488836	0.000344313	0.002405002
TR13814-c2_g11	130	54	1.267480311	2.11088E-08	3.10717E-07
TR22906-c0_g1	60	25	1.263034406	0.000147356	0.001121421
TR7805-c0_g1	36	15	1.263034406	0.00328287	0.016473768
TR11160-c1_g1	247	103	1.261866704	1.40398E-14	3.68927E-13
TR21703-c0_g1	175	73	1.261386553	9.43762E-11	1.80689E-09
TR4209-c0_g1	194	81	1.260062839	9.58852E-12	2.03772E-10
TR3906-c0_g1	55	23	1.257797757	0.00029247	0.002070098
TR10318-c1_g10	153	64	1.257387843	1.54485E-09	2.58764E-08
TR15022-c0_g1	251	105	1.257298036	1.02846E-14	2.72354E-13
TR7546-c0_g1	43	18	1.256339753	0.001376651	0.00802912
TR19810-c0_g1	148	62	1.255257055	2.99155E-09	4.86237E-08
TR3900-c0_g1	186	78	1.253756592	3.05399E-11	6.17383E-10
TR12128-c0_g17	50	21	1.251538767	0.000582571	0.003793365
TR14630-c0_g1	50	21	1.251538767	0.000582571	0.003793365
TR3795-c0_g1	69	29	1.250543462	5.38873E-05	0.00045287
TR14373-c0_g1	38	16	1.247927513	0.002774804	0.014567361

TR20682-c0_g1	140	59	1.246639968	9.58929E-09	1.46874E-07
TR21589-c1_g1	500	211	1.244685096	2.42635E-27	1.24626E-25
TR20775-c0_g2	187	79	1.243113712	3.67634E-11	7.35325E-10
TR8508-c3_g1	1517	641	1.242824824	2.63425E-79	4.14682E-77
TR26739-c0_g1	111	47	1.239827015	3.6437E-07	4.55833E-06
TR1497-c0_g1	59	25	1.23878686	0.00021063	0.001557629
TR17543-c0_g1	245	104	1.236198221	4.70082E-14	1.19106E-12
TR18416-c0_g1	40	17	1.234465254	0.002343584	0.012494207
TR19727-c0_g1	68	29	1.229481846	7.66667E-05	0.000621868
TR13692-c0_g1	180	77	1.225066556	1.40018E-10	2.62748E-09
TR3968-c0_g1	264	113	1.224215157	8.09819E-15	2.17504E-13
TR18893-c0_g1	63	27	1.222392421	0.000151392	0.00115007
TR16162-c0_g1	49	21	1.222392421	0.000833691	0.005210973
TR10499-c0_g1	42	18	1.222392421	0.001978199	0.010859539
TR7594-c0_g1	42	18	1.222392421	0.001978199	0.010859539
TR3838-c0_g1	35	15	1.222392421	0.00474369	0.022853219
TR10318-c1_g7	51	22	1.212993723	0.00070464	0.004493552
TR15880-c0_g1	51	22	1.212993723	0.00070464	0.004493552
TR5594-c0_g1	51	22	1.212993723	0.00070464	0.004493552
TR6178-c0_g1	88	38	1.211504105	8.75696E-06	8.62572E-05
TR15431-c0_g1	44	19	1.211504105	0.001669002	0.009349955
TR14756-c0_g1	37	16	1.209453366	0.003992626	0.019388724
TR6207-c1_g1	351	152	1.207399707	8.29445E-19	2.77592E-17
TR11289-c1_g1	600	260	1.206450877	5.64979E-31	3.35853E-29
TR15477-c0_g1	180	78	1.206450877	2.33861E-10	4.30763E-09
TR14802-c0_g1	90	39	1.206450877	7.4327E-06	7.45521E-05
TR14633-c0_g1	60	26	1.206450877	0.000253614	0.001820084
TR4885-c0_g1	76	33	1.203533394	3.96348E-05	0.000345916
TR6766-c0_g2	76	33	1.203533394	3.96348E-05	0.000345916
TR18192-c0_g1	115	50	1.201633861	4.44352E-07	5.45851E-06
TR26739-c1_g4	115	50	1.201633861	4.44352E-07	5.45851E-06
TR11158-c1_g1	430	187	1.20129839	1.6307E-22	6.64848E-21
TR15275-c1_g1	85	37	1.199937571	1.45327E-05	0.000138262
TR13807-c2_g1	572	249	1.199869405	2.32281E-29	1.29904E-27
TR342-c0_g1	39	17	1.197939378	0.003359277	0.016837311
TR25894-c0_g2	71	31	1.195550809	7.78897E-05	0.000631186
TR4885-c0_g2	73	32	1.189824559	6.59259E-05	0.000544884
TR16137-c0_g1	41	18	1.187627003	0.002825697	0.014747006
TR958-c0_g1	50	22	1.184424571	0.001000478	0.006083497
TR14588-c0_g1	84	37	1.182864057	2.04098E-05	0.000188994
TR6524-c2_g1	120	53	1.178970141	3.80225E-07	4.72884E-06
TR13809-c0_g1	242	107	1.177396251	5.81421E-13	1.35596E-11
TR10015-c0_g1	52	23	1.176877762	0.00084324	0.005268725
TR14468-c0_g1	97	43	1.173648087	5.38437E-06	5.49469E-05

TR6100-c0_g2	97	43	1.173648087	5.38437E-06	5.49469E-05
TR6427-c0_g1	266	118	1.172639386	5.11763E-14	1.29281E-12
TR593-c2_g1	286	127	1.17118665	6.23908E-15	1.69716E-13
TR18147-c0_g1	54	24	1.169925001	0.00071063	0.004528353
TR22030-c0_g1	45	20	1.169925001	0.001998478	0.010921494
TR20324-c0_g1	247	110	1.167007518	4.95324E-13	1.15994E-11
TR21402-c0_g2	47	21	1.162271429	0.001680518	0.009398366
TR18254-c0_g1	38	17	1.160464672	0.004784337	0.023042511
TR2070-c0_g1	96	43	1.158697746	7.50937E-06	7.52767E-05
TR21589-c1_g2	569	255	1.157931405	1.14295E-27	6.01602E-26
TR25280-c0_g1	87	39	1.157541277	2.04321E-05	0.000189098
TR2070-c0_g2	58	26	1.157541277	0.000504581	0.003344463
TR6100-c0_g1	58	26	1.157541277	0.000504581	0.003344463
TR24883-c0_g1	524	235	1.156906055	1.41948E-25	6.62994E-24
TR25928-c0_g2	69	31	1.154328146	0.000153543	0.001164854
TR3412-c0_g1	405	182	1.153983458	4.723E-20	1.69762E-18
TR4829-c0_g2	60	27	1.152003093	0.000425157	0.002900575
TR950-c0_g1	71	32	1.14974712	0.000129564	0.001002921
TR10136-c0_g1	144	65	1.147557188	5.25904E-08	7.39541E-07
TR10559-c0_g1	42	19	1.144389909	0.00336374	0.016849746
TR18790-c0_g1	42	19	1.144389909	0.00336374	0.016849746
TR516-c1_g1	130	59	1.139724764	2.71748E-07	3.44525E-06
TR8500-c1_g1	2812	1277	1.138838069	1.2678E-126	3.1237E-124
TR15443-c0_g1	77	35	1.137503524	7.78372E-05	0.000631062
TR3299-c0_g1	46	21	1.131244533	0.002366346	0.012607652
TR529-c0_g1	179	82	1.126263773	2.29461E-09	3.77287E-08
TR26368-c0_g1	85	39	1.123988717	3.94589E-05	0.000344735
TR3893-c0_g1	50	23	1.120294234	0.001665895	0.009342828
TR4091-c0_g1	50	23	1.120294234	0.001665895	0.009342828
TR14474-c0_g1	252	116	1.119298928	1.73614E-12	3.93032E-11
TR11137-c0_g1	89	41	1.118181426	2.80983E-05	0.000252355
TR26278-c0_g1	115	53	1.117569596	1.94633E-06	2.14871E-05
TR8526-c4_g1	295	136	1.117108303	2.53226E-14	6.55278E-13
TR8065-c1_g1	117	54	1.115477217	1.64531E-06	1.83787E-05
TR13810-c3_g2	52	24	1.115477217	0.001398164	0.008139916
TR14882-c0_g1	52	24	1.115477217	0.001398164	0.008139916
TR4039-c0_g1	212	98	1.11321061	1.18686E-10	2.23953E-09
TR9522-c0_g1	54	25	1.111031312	0.001173693	0.006962435
TR3242-c0_g1	41	19	1.109624491	0.004731848	0.022809107
TR9464-c0_g1	41	19	1.109624491	0.004731848	0.022809107
TR8688-c0_g2	226	105	1.105933445	3.72932E-11	7.45046E-10
TR18171-c0_g1	43	20	1.10433666	0.003958635	0.019376588
TR12918-c1_g1	60	28	1.099535674	0.000695117	0.004437817
TR14544-c0_g1	77	36	1.096861539	0.0001259	0.000978278

TR21688-c0_g1	62	29	1.096215315	0.000583975	0.00380105
TR26938-c0_g1	47	22	1.095157233	0.002773658	0.014567361
TR4829-c0_g1	79	37	1.094327383	0.000106014	0.000835985
TR9524-c1_g1	2825	1324	1.093347745	4.4891E-119	1.0749E-116
TR6495-c0_g1	64	30	1.093109404	0.000490696	0.003256235
TR10299-c2_g2	1104	518	1.091716169	1.84599E-47	1.69644E-45
TR27174-c1_g1	49	23	1.091147888	0.002322927	0.012387968
TR21725-c0_g2	85	40	1.087462841	6.33381E-05	0.000525284
TR8470-c0_g1	51	24	1.087462841	0.001946095	0.010693664
TR20356-c0_g1	53	25	1.084064265	0.001630921	0.009205779
TR12609-c0_g1	178	84	1.083416008	7.88533E-09	1.21763E-07
TR21586-c0_g3	55	26	1.080919995	0.001367214	0.007985037
TR14891-c2_g1	1120	532	1.074000581	6.99416E-47	6.25842E-45
TR11123-c0_g1	80	38	1.074000581	0.000122713	0.00095526
TR10360-c0_g12	141	67	1.073462162	3.45332E-07	4.32335E-06
TR25850-c0_g2	61	29	1.072756342	0.000806872	0.005050772
TR13725-c0_g1	84	40	1.070389328	8.69163E-05	0.000698342
TR24338-c0_g1	84	40	1.070389328	8.69163E-05	0.000698342
TR13421-c0_g1	69	33	1.064130337	0.000400884	0.00275822
TR16170-c0_g1	71	34	1.062284278	0.000336765	0.002355186
TR14145-c0_g1	50	24	1.058893689	0.002694322	0.014216957
TR20348-c0_g1	52	25	1.056583528	0.002254571	0.012030978
TR21586-c0_g2	54	26	1.054447784	0.001887373	0.010421513
TR9369-c0_g1	58	28	1.050626073	0.001324173	0.007736324
TR12598-c0_g1	120	58	1.0489096	3.98658E-06	4.1709E-05
TR19797-c0_g1	120	58	1.0489096	3.98658E-06	4.1709E-05
TR25626-c0_g3	60	29	1.0489096	0.00110974	0.006657389
TR836-c0_g1	216	105	1.040641984	7.88391E-10	1.37756E-08
TR12128-c0_g14	72	35	1.040641984	0.000386975	0.002664675
TR20302-c0_g1	1129	549	1.040167432	7.04843E-45	6.02174E-43
TR7752-c0_g1	80	39	1.036525876	0.000192712	0.001433853
TR9534-c0_g1	209	102	1.03493379	1.75532E-09	2.91149E-08
TR449-c0_g1	86	42	1.033947332	0.000114499	0.000898725
TR16697-c0_g1	98	48	1.029747343	4.06167E-05	0.000351419
TR13121-c0_g1	49	24	1.029747343	0.003709766	0.01828673
TR21749-c0_g1	49	24	1.029747343	0.003709766	0.01828673
TR3985-c0_g1	118	58	1.024662054	7.30385E-06	7.33895E-05
TR9429-c0_g1	61	30	1.023846742	0.001272332	0.00745908
TR21258-c0_g2	132	65	1.022026306	2.21235E-06	2.41263E-05
TR10066-c0_g1	233	115	1.018696094	3.57976E-10	6.50916E-09
TR19963-c0_g1	150	75	1	7.2874E-07	8.62181E-06
TR21258-c0_g1	128	64	1	4.74343E-06	4.90538E-05
TR16384-c0_g1	116	58	1	1.32445E-05	0.000126787

TR25850-c0_g1	66	33	1	0.0010175	0.006153983
TR25203-c0_g1	58	29	1	0.002069535	0.011259148
TR7590-c0_g1	58	29	1	0.002069535	0.011259148
TR25928-c0_g1	56	28	1	0.002474081	0.013111797
TR12128-c0_g6	52	26	1	0.003541017	0.017608065
TR12596-c3_g1	101	202	-1	2.39556E-09	3.92367E-08
TR484-c0_g1	48	96	-1	3.88422E-05	0.000340095
TR23194-c0_g1	41	82	-1	0.000143293	0.001092289
TR710-c0_g1	30	60	-1	0.001143791	0.006789793
TR13721-c0_g1	386	774	-1.003732719	9.80163E-32	5.99427E-30
TR856-c0_g1	92	185	-1.007819504	9.04415E-09	1.389E-07
TR460-c5_g1	154	310	-1.009337865	9.33837E-14	2.33135E-12
TR515-c2_g1	117	236	-1.01227833	7.25946E-11	1.39458E-09
TR2562-c3_g1	195	394	-1.014721506	3.30121E-17	1.02231E-15
TR2820-c0_g1	45	91	-1.015941544	4.96794E-05	0.000421044
TR1663-c0_g1	32	65	-1.022367813	0.000568313	0.003717611
TR21017-c0_g1	31	63	-1.023083613	0.000686847	0.004391619
TR13635-c1_g1	111	226	-1.025763096	1.16414E-10	2.20155E-09
TR21979-c0_g1	308	628	-1.027834208	5.21331E-27	2.61455E-25
TR3233-c1_g1	329	672	-1.030373649	6.78219E-29	3.70759E-27
TR531-c1_g1	244	499	-1.032158668	6.28844E-22	2.49212E-20
TR24479-c0_g1	22	45	-1.032421478	0.003851035	0.018950126
TR11269-c2_g1	322	661	-1.037589583	9.4546E-29	5.13548E-27
TR3223-c1_g1	158	325	-1.04051516	5.40633E-15	1.48011E-13
TR20334-c0_g1	162	334	-1.04385429	1.94193E-15	5.41748E-14
TR6726-c1_g2	64	132	-1.044394119	5.84865E-07	7.09235E-06
TR2820-c0_g2	48	99	-1.044394119	1.51355E-05	0.000143192
TR1001-c0_g1	77	159	-1.046096415	4.00547E-08	5.70361E-07
TR11272-c3_g1	197	407	-1.046833165	1.48506E-18	4.88276E-17
TR9360-c0_g2	44	91	-1.048363022	3.17118E-05	0.000283312
TR6726-c1_g1	58	120	-1.0489096	1.75211E-06	1.95332E-05
TR5188-c0_g1	28	58	-1.050626073	0.000876422	0.005371424
TR15859-c1_g1	108	224	-1.05246742	5.79302E-11	1.12946E-09
TR7803-c0_g1	26	54	-1.054447784	0.001281899	0.007512572
TR153-c0_g1	128	266	-1.055282436	8.5504E-13	1.96976E-11
TR3989-c1_g1	163	339	-1.056413309	6.41018E-16	1.84402E-14
TR8653-c0_g2	85	177	-1.058214614	5.01228E-09	7.91229E-08
TR8467-c0_g1	282	588	-1.060120992	1.33974E-26	6.58304E-25
TR23919-c0_g1	35	73	-1.060541542	0.000169103	0.001269869
TR11281-c0_g1	58	121	-1.060882242	1.27417E-06	1.44418E-05
TR25318-c0_g1	44	92	-1.064130337	2.29731E-05	0.000210212
TR11159-c0_g1	22	46	-1.064130337	0.002756185	0.01449839
TR523-c1_g1	692	1448	-1.065217659	1.63066E-63	2.06891E-61
TR851-c7_g1	1527	3204	-1.069174086	2.5881E-139	7.3336E-137

TR14638-c0_g1	20	42	-1.070389328	0.004053274	0.019672004
TR27084-c0_g1	20	42	-1.070389328	0.004053274	0.019672004
TR15279-c0_g1	29	61	-1.072756342	0.000521002	0.003434557
TR9366-c0_g1	29	61	-1.072756342	0.000521002	0.003434557
TR15969-c0_g1	19	40	-1.074000581	0.004919496	0.023593771
TR10100-c1_g1	244	514	-1.074887212	6.08502E-24	2.65265E-22
TR14606-c0_g1	28	59	-1.075288127	0.000629036	0.004058612
TR2429-c0_g1	37	78	-1.075948853	8.39389E-05	0.000677622
TR13285-c0_g1	26	55	-1.080919995	0.000917748	0.005616608
TR802-c0_g1	110	233	-1.082826431	8.34293E-12	1.78866E-10
TR24965-c0_g2	25	53	-1.084064265	0.001109038	0.00665553
TR3583-c0_g2	102	217	-1.08912589	3.49197E-11	7.00095E-10
TR20308-c4_g1	145	309	-1.091553938	2.39356E-15	6.66019E-14
TR3583-c0_g1	99	211	-1.091742569	5.97392E-11	1.1634E-09
TR153-c0_g2	105	224	-1.093109404	1.48119E-11	3.071E-10
TR18778-c3_g1	566	1211	-1.097324907	6.75014E-56	7.7021E-54
TR8653-c0_g1	85	182	-1.098403704	1.01306E-09	1.74502E-08
TR27634-c0_g1	77	165	-1.099535674	5.89154E-09	9.23171E-08
TR4056-c0_g1	21	45	-1.099535674	0.002372996	0.012639118
TR19746-c0_g1	57	123	-1.109624491	4.16724E-07	5.1414E-06
TR10058-c1_g1	106	229	-1.111283334	4.69838E-12	1.03202E-10
TR597-c8_g1	165	357	-1.11345805	5.27871E-18	1.6933E-16
TR856-c0_g2	84	182	-1.115477217	6.31525E-10	1.11032E-08
TR18185-c0_g1	30	65	-1.115477217	0.000220395	0.001622963
TR8123-c0_g1	24	52	-1.115477217	0.00095194	0.005794572
TR20344-c0_g1	100	217	-1.117695043	1.3664E-11	2.84341E-10
TR27501-c0_g2	64	139	-1.118941073	6.09485E-08	8.45881E-07
TR19925-c0_g1	60	131	-1.126532406	1.25068E-07	1.6586E-06
TR24965-c0_g1	21	46	-1.131244533	0.001675259	0.00937512
TR9360-c0_g1	36	79	-1.133855747	3.69524E-05	0.000326019
TR18848-c0_g1	231	507	-1.134092896	1.3731E-25	6.44875E-24
TR15926-c4_g1	158	347	-1.135011104	4.92698E-18	1.58646E-16
TR3873-c0_g1	343	754	-1.136355947	2.31688E-37	1.68333E-35
TR25519-c0_g1	50	110	-1.137503524	1.05654E-06	1.22194E-05
TR10518-c0_g1	54	119	-1.139930261	3.6745E-07	4.59348E-06
TR4012-c0_g1	353	778	-1.140101972	1.09309E-38	8.04503E-37
TR8549-c5_g1	143	316	-1.143909411	9.56057E-17	2.87177E-15
TR15478-c0_g1	57	126	-1.144389909	1.53245E-07	2.02594E-06
TR7581-c0_g1	33	73	-1.14543044	6.39418E-05	0.000530032
TR26986-c0_g1	73	162	-1.150025444	2.30807E-09	3.79133E-08
TR13660-c0_g1	224	501	-1.161311871	3.338E-26	1.63076E-24
TR10025-c6_g1	2444	5474	-1.16335115	1.1304E-270	7.6873E-268
TR6752-c1_g1	25	56	-1.163498732	0.000393116	0.002705869
TR13764-c1_g1	203	455	-1.164386818	4.87891E-24	2.13234E-22

TR25853-c0_g1	41	92	-1.166009951	5.33667E-06	5.45911E-05
TR15452-c0_g1	94	211	-1.166510337	5.38386E-12	1.175E-10
TR15719-c0_g1	68	153	-1.169925001	3.98881E-09	6.3856E-08
TR10037-c0_g1	24	54	-1.169925001	0.000472625	0.003144911
TR25115-c0_g1	16	36	-1.169925001	0.004313343	0.020886512
TR11288-c3_g1	103	232	-1.171480468	4.04032E-13	9.55365E-12
TR7766-c1_g1	102	230	-1.173064709	4.81263E-13	1.12968E-11
TR26351-c0_g1	23	52	-1.176877762	0.000568175	0.003717611
TR17465-c0_g3	19	43	-1.178337241	0.001706967	0.009524354
TR7758-c8_g1	2418	5490	-1.182991905	1.3806E-278	9.7797E-276
TR22943-c1_g1	103	234	-1.183864192	2.051E-13	4.97428E-12
TR2777-c0_g1	22	50	-1.184424571	0.000682974	0.004370138
TR26993-c0_g1	54	123	-1.187627003	9.37901E-08	1.26552E-06
TR7536-c0_g1	28	64	-1.192645078	0.000111875	0.000880569
TR491-c0_g1	38	87	-1.195015982	6.44306E-06	6.53974E-05
TR13770-c0_g1	55	126	-1.19592021	5.5598E-08	7.78692E-07
TR10987-c0_g2	17	39	-1.197939378	0.002477054	0.013123464
TR10710-c0_g1	145	333	-1.199469277	8.53619E-19	2.85121E-17
TR8509-c0_g1	20	46	-1.201633861	0.000986365	0.005999826
TR3221-c0_g1	16	37	-1.209453366	0.00298309	0.015332054
TR502-c3_g1	127	294	-1.210987658	5.29472E-17	1.61321E-15
TR3108-c0_g1	22	51	-1.212993723	0.000475439	0.003159928
TR25839-c0_g2	72	168	-1.222392421	1.76714E-10	3.28706E-09
TR9193-c0_g1	30	70	-1.222392421	3.81751E-05	0.000335448
TR2777-c0_g4	21	49	-1.222392421	0.00056992	0.003726692
TR22796-c0_g1	18	42	-1.222392421	0.001423036	0.008265629
TR12701-c0_g1	15	35	-1.222392421	0.003591344	0.017762134
TR17448-c0_g1	35	82	-1.228268988	7.68168E-06	7.66914E-05
TR2403-c0_g1	67	157	-1.228531558	5.9486E-10	1.0502E-08
TR25260-c0_g1	34	80	-1.234465254	9.15783E-06	9.01536E-05
TR3881-c0_g1	28	66	-1.237039197	5.44201E-05	0.000457121
TR26722-c0_g1	14	33	-1.237039197	0.004321623	0.020920648
TR6511-c0_g1	235	556	-1.242424126	6.56745E-32	4.03088E-30
TR12879-c3_g1	134	318	-1.246793765	4.93717E-19	1.67877E-17
TR12701-c0_g2	16	38	-1.247927513	0.00204938	0.011156634
TR8569-c0_g1	140	334	-1.254421276	4.38544E-20	1.57962E-18
TR11195-c0_g1	31	74	-1.255257055	1.5481E-05	0.00014614
TR8511-c0_g1	36	86	-1.256339753	3.13002E-06	3.3595E-05
TR11850-c4_g1	41	98	-1.257157839	6.38269E-07	7.71794E-06
TR25685-c0_g1	30	72	-1.263034406	1.84249E-05	0.000172588
TR22318-c0_g2	15	36	-1.263034406	0.002456905	0.013024831
TR10570-c0_g1	22	53	-1.268488836	0.000226966	0.001661811
TR2032-c0_g1	17	41	-1.270089163	0.00117076	0.006947458
TR7661-c0_g1	24	58	-1.273018494	0.000109966	0.000865941

TR27305-c0_g1	19	46	-1.275634443	0.000561367	0.003675003
TR2653-c0_g1	21	51	-1.280107919	0.00027052	0.001937322
TR6452-c0_g1	170	414	-1.284096021	2.39358E-25	1.09392E-23
TR27571-c0_g1	16	39	-1.285402219	0.001398932	0.008139916
TR14962-c0_g1	18	44	-1.289506617	0.000669522	0.004285675
TR2777-c1_g1	18	44	-1.289506617	0.000669522	0.004285675
TR8154-c0_g1	44	108	-1.295455884	8.87122E-08	1.19891E-06
TR5876-c0_g1	22	54	-1.295455884	0.000155691	0.001179044
TR17936-c1_g1	48	118	-1.297680549	2.16946E-08	3.18787E-07
TR17590-c0_g1	13	32	-1.299560282	0.003520349	0.017510414
TR10507-c0_g1	15	37	-1.30256277	0.001669662	0.009349955
TR19056-c0_g1	15	37	-1.30256277	0.001669662	0.009349955
TR5498-c0_g2	15	37	-1.30256277	0.001669662	0.009349955
TR25444-c0_g1	17	42	-1.304854582	0.000797652	0.005005959
TR2653-c0_g2	21	52	-1.308122295	0.000185	0.00137949
TR20138-c0_g2	25	62	-1.310340121	4.35803E-05	0.000375341
TR8067-c0_g1	27	67	-1.311201688	2.12449E-05	0.00019598
TR10052-c0_g1	170	422	-1.311708253	1.33522E-26	6.57984E-25
TR17459-c0_g1	33	82	-1.313157885	2.49382E-06	2.70224E-05
TR17587-c0_g1	35	87	-1.313660479	1.22539E-06	1.39353E-05
TR26937-c5_g1	524	1308	-1.319723824	1.39094E-79	2.21007E-77
TR26416-c0_g1	54	135	-1.321928095	1.26043E-09	2.13863E-08
TR6483-c0_g1	44	110	-1.321928095	4.22284E-08	5.97784E-07
TR14586-c0_g1	36	90	-1.321928095	7.1244E-07	8.46431E-06
TR8111-c0_g1	36	90	-1.321928095	7.1244E-07	8.46431E-06
TR12422-c0_g1	18	45	-1.321928095	0.000455393	0.003071373
TR17993-c0_g2	18	45	-1.321928095	0.000455393	0.003071373
TR20153-c0_g1	18	45	-1.321928095	0.000455393	0.003071373
TR6151-c0_g2	14	35	-1.321928095	0.001989951	0.010888033
TR7526-c1_g1	213	533	-1.323282103	1.38553E-33	9.05995E-32
TR15864-c0_g1	85	213	-1.325318684	2.12898E-14	5.52602E-13
TR13773-c3_g1	495	1245	-1.330645312	9.19174E-77	1.43368E-74
TR11214-c0_g1	161	405	-1.33086122	3.98108E-26	1.91197E-24
TR10070-c0_g1	25	63	-1.333423734	2.97604E-05	0.000266298
TR27497-c0_g1	213	539	-1.339431843	1.53519E-34	1.0482E-32
TR20153-c0_g2	15	38	-1.341036918	0.001127448	0.006756472
TR6485-c0_g1	15	38	-1.341036918	0.001127448	0.006756472
TR25549-c0_g1	22	56	-1.347923303	7.22614E-05	0.000591497
TR12686-c0_g1	20	51	-1.350497247	0.000148941	0.001132973
TR851-c0_g1	190	485	-1.351985329	1.15005E-31	7.00804E-30
TR17577-c0_g1	38	97	-1.351985329	1.64794E-07	2.15849E-06
TR461-c0_g1	18	46	-1.353636955	0.000308095	0.002173455
TR3086-c0_g1	25	64	-1.35614381	2.02428E-05	0.000187652
TR27084-c0_g2	16	41	-1.357552005	0.000640071	0.004126682

TR8287-c2_g3	110	282	-1.358191639	3.30646E-19	1.13335E-17
TR21311-c2_g1	184	473	-1.362134417	2.96904E-31	1.78999E-29
TR25083-c0_g1	268	689	-1.362270982	9.28397E-45	7.89199E-43
TR20363-c0_g1	33	85	-1.364996817	7.97238E-07	9.393E-06
TR4717-c0_g1	19	49	-1.366782331	0.000175902	0.001318082
TR19567-c0_g1	31	80	-1.367731785	1.61992E-06	1.81071E-05
TR1149-c0_g1	72	186	-1.36923381	2.49629E-13	6.03701E-12
TR5498-c0_g1	17	44	-1.371968777	0.00036403	0.002532324
TR7552-c0_g1	17	44	-1.371968777	0.00036403	0.002532324
TR1445-c0_g2	378	979	-1.372922625	1.40846E-63	1.80043E-61
TR3913-c4_g1	22	57	-1.373458396	4.89058E-05	0.000414902
TR3977-c0_g1	25	65	-1.378511623	1.3716E-05	0.000131153
TR451-c4_g1	143	372	-1.379287474	2.26469E-25	1.03781E-23
TR1445-c0_g1	404	1051	-1.379335471	1.38043E-68	1.89267E-66
TR18158-c0_g1	56	146	-1.382469637	6.52363E-11	1.26322E-09
TR15914-c0_g1	26	68	-1.387023123	7.89256E-06	7.86081E-05
TR13052-c0_g1	21	55	-1.389042291	5.75385E-05	0.000481176
TR2777-c0_g3	21	55	-1.389042291	5.75385E-05	0.000481176
TR15948-c7_g1	128	336	-1.392317423	2.24312E-23	9.43959E-22
TR3981-c0_g1	78	205	-1.394077881	6.81595E-15	1.84523E-13
TR22000-c0_g1	19	50	-1.395928676	0.000118294	0.000925098
TR17524-c0_g1	22	58	-1.398549376	3.2959E-05	0.000293375
TR10018-c2_g1	166	438	-1.399747628	3.34529E-30	1.92794E-28
TR25685-c0_g2	35	93	-1.409875794	1.22798E-07	1.63231E-06
TR8057-c0_g1	38	101	-1.410283969	3.52183E-08	5.0528E-07
TR15854-c0_g1	18	48	-1.415037499	0.000138876	0.001061158
TR7543-c0_g1	184	492	-1.418952549	2.2492E-34	1.52348E-32
TR25083-c0_g2	340	912	-1.423499078	1.57188E-62	1.965E-60
TR4134-c1_g1	171	459	-1.424497829	2.33598E-32	1.44418E-30
TR24927-c0_g1	16	43	-1.426264755	0.000286263	0.002027853
TR4082-c1_g1	374	1006	-1.42752013	4.51784E-69	6.29584E-67
TR11388-c0_g1	13	35	-1.428843299	0.001049161	0.00632298
TR15476-c2_g1	139	375	-1.431805713	6.26745E-27	3.12478E-25
TR10085-c1_g1	10	27	-1.432959407	0.003922394	0.019251121
TR5202-c0_g1	10	27	-1.432959407	0.003922394	0.019251121
TR18972-c1_g1	71	192	-1.435215381	1.34832E-14	3.55398E-13
TR3822-c0_g1	17	46	-1.436099115	0.000162636	0.001226177
TR2798-c0_g1	100	271	-1.438292852	4.99133E-20	1.78276E-18
TR9513-c2_g1	108	293	-1.439869352	1.47394E-21	5.72123E-20
TR8854-c0_g2	32	87	-1.442943496	1.94729E-07	2.52336E-06
TR19910-c0_g1	54	147	-1.444784843	1.2748E-11	2.67571E-10
TR957-c0_g1	22	60	-1.447458977	1.47871E-05	0.000140526
TR19056-c0_g2	11	30	-1.447458977	0.002191132	0.01171083
TR26392-c0_g1	15	41	-1.450661409	0.000334937	0.002344329

TR554-c2_g1	264	722	-1.451460908	2.85958E-51	2.82655E-49
TR16036-c1_g1	184	504	-1.453717967	2.175E-36	1.56686E-34
TR8196-c2_g2	109	299	-1.455817349	2.67064E-22	1.07086E-20
TR19773-c0_g1	16	44	-1.459431619	0.000189912	0.001413639
TR22894-c0_g1	16	44	-1.459431619	0.000189912	0.001413639
TR9148-c0_g1	16	44	-1.459431619	0.000189912	0.001413639
TR3163-c0_g1	12	33	-1.459431619	0.001229055	0.007252883
TR934-c1_g1	274	754	-1.46038863	6.22364E-54	6.53149E-52
TR24294-c0_g1	361	998	-1.467040978	2.84718E-71	4.10219E-69
TR23635-c0_g1	70	194	-1.470629825	3.24236E-15	8.96333E-14
TR26941-c2_g1	245	680	-1.472752997	2.13896E-49	2.0662E-47
TR8148-c0_g1	27	75	-1.473931188	9.19859E-07	1.07336E-05
TR27643-c0_g1	18	50	-1.473931188	6.14024E-05	0.000510702
TR11850-c3_g1	73	203	-1.475511358	6.39408E-16	1.84251E-14
TR15873-c5_g1	2824	7861	-1.476972761	0	0
TR5153-c0_g2	89	248	-1.478462879	3.69341E-19	1.26344E-17
TR16472-c0_g1	33	92	-1.479167837	5.0347E-08	7.08581E-07
TR11859-c0_g1	40	112	-1.485426827	1.6193E-09	2.70701E-08
TR11850-c2_g1	25	70	-1.485426827	1.8549E-06	2.05578E-05
TR450-c5_g1	25	70	-1.485426827	1.8549E-06	2.05578E-05
TR12105-c1_g2	20	56	-1.485426827	1.99763E-05	0.000185283
TR21849-c0_g1	10	28	-1.485426827	0.00256237	0.013550132
TR22497-c0_g1	10	28	-1.485426827	0.00256237	0.013550132
TR9341-c0_g1	10	28	-1.485426827	0.00256237	0.013550132
TR9302-c0_g1	27	76	-1.493040011	6.12066E-07	7.41164E-06
TR12107-c0_g1	11	31	-1.494764692	0.001433231	0.008319161
TR13068-c0_g1	11	31	-1.494764692	0.001433231	0.008319161
TR16480-c0_g1	17	48	-1.497499659	7.12344E-05	0.000584216
TR1052-c0_g1	18	51	-1.502500341	4.05499E-05	0.00035102
TR20351-c0_g1	27	77	-1.511899039	4.06015E-07	5.01292E-06
TR13700-c0_g1	217	622	-1.519219538	2.39151E-47	2.17427E-45
TR4660-c0_g1	15	43	-1.519374159	0.000145074	0.001104546
TR11267-c3_g2	40	115	-1.523561956	4.78641E-10	8.58389E-09
TR7791-c0_g1	25	72	-1.526068812	8.13575E-07	9.57884E-06
TR18583-c0_g1	9	26	-1.530514717	0.002978146	0.015332054
TR25602-c0_g2	9	26	-1.530514717	0.002978146	0.015332054
TR4003-c3_g1	2714	7844	-1.531168815	0	0
TR93-c0_g2	10	29	-1.5360529	0.001661714	0.009342828
TR8465-c0_g1	25	73	-1.545968369	5.36235E-07	6.52125E-06
TR12274-c2_g1	101	295	-1.546359661	6.76792E-24	2.93942E-22
TR6452-c1_g1	90	263	-1.547065893	1.72115E-21	6.65043E-20
TR11798-c0_g2	16	47	-1.554588852	5.38246E-05	0.000452567
TR14100-c0_g1	17	50	-1.556393349	3.06292E-05	0.000273784
TR5139-c1_g2	71	209	-1.557612013	1.48296E-17	4.66032E-16

TR12593-c8_g1	143	421	-1.557805086	9.74088E-34	6.41891E-32
TR12805-c3_g1	356	1049	-1.559065532	1.4909E-81	2.50964E-79
TR15601-c6_g1	85	252	-1.567888987	4.99582E-21	1.90438E-19
TR568-c0_g1	45	134	-1.574236094	5.95175E-12	1.29396E-10
TR21504-c2_g1	45	135	-1.584962501	3.92042E-12	8.63373E-11
TR3798-c0_g1	39	117	-1.584962501	1.04185E-10	1.98797E-09
TR21990-c0_g1	36	108	-1.584962501	5.39161E-10	9.54839E-09
TR548-c0_g1	22	66	-1.584962501	1.22014E-06	1.38849E-05
TR17572-c0_g1	12	36	-1.584962501	0.000338843	0.002368745
TR1213-c0_g1	10	30	-1.584962501	0.00107021	0.006442982
TR1663-c0_g2	10	30	-1.584962501	0.00107021	0.006442982
TR20375-c0_g1	10	30	-1.584962501	0.00107021	0.006442982
TR11645-c0_g1	9	27	-1.584962501	0.00191242	0.010512017
TR17139-c0_g1	9	27	-1.584962501	0.00191242	0.010512017
TR2509-c0_g1	9	27	-1.584962501	0.00191242	0.010512017
TR27762-c0_g2	9	27	-1.584962501	0.00191242	0.010512017
TR9088-c0_g1	9	27	-1.584962501	0.00191242	0.010512017
TR12107-c0_g2	8	24	-1.584962501	0.003433402	0.01717845
TR18074-c1_g1	8	24	-1.584962501	0.003433402	0.01717845
TR3196-c0_g1	8	24	-1.584962501	0.003433402	0.01717845
TR562-c0_g1	8	24	-1.584962501	0.003433402	0.01717845
TR3982-c1_g1	81	244	-1.590887335	8.31948E-21	3.10862E-19
TR11169-c0_g1	32	97	-1.599912842	3.17843E-09	5.14256E-08
TR13256-c2_g1	627	1901	-1.600221184	8.4355E-152	2.6075E-149
TR16035-c0_g1	23	70	-1.605721061	4.56537E-07	5.59202E-06
TR3210-c0_g1	39	119	-1.609415544	4.48446E-11	8.88598E-10
TR19756-c0_g1	12	37	-1.624490865	0.000217987	0.001607835
TR16725-c0_g1	11	34	-1.628031223	0.000385814	0.002657756
TR1775-c0_g2	616	1906	-1.629545863	4.1607E-156	1.3347E-153
TR621-c5_g1	344	1066	-1.631726968	2.87848E-88	5.49864E-86
TR14386-c0_g1	10	31	-1.632268215	0.000684764	0.004379946
TR22538-c0_g1	107	332	-1.633572445	1.00013E-28	5.41516E-27
TR851-c6_g1	328	1019	-1.635386332	1.02191E-84	1.79112E-82
TR1104-c0_g1	9	28	-1.637429921	0.001219352	0.007198123
TR13784-c0_g1	9	28	-1.637429921	0.001219352	0.007198123
TR18277-c0_g1	9	28	-1.637429921	0.001219352	0.007198123
TR25876-c0_g1	9	28	-1.637429921	0.001219352	0.007198123
TR93-c0_g1	9	28	-1.637429921	0.001219352	0.007198123
TR1453-c0_g1	35	109	-1.638901308	1.70617E-10	3.17713E-09
TR25061-c0_g1	17	53	-1.640457613	8.36367E-06	8.29117E-05
TR546-c0_g1	17	53	-1.640457613	8.36367E-06	8.29117E-05
TR9369-c1_g1	17	53	-1.640457613	8.36367E-06	8.29117E-05
TR7782-c0_g1	16	50	-1.64385619	1.4641E-05	0.000139215
TR10358-c0_g1	8	25	-1.64385619	0.002179766	0.011653747

TR12274-c1_g1	8	25	-1.64385619	0.002179766	0.011653747
TR22186-c0_g1	8	25	-1.64385619	0.002179766	0.011653747
TR24305-c0_g1	8	25	-1.64385619	0.002179766	0.011653747
TR8266-c0_g1	8	25	-1.64385619	0.002179766	0.011653747
TR13763-c6_g1	406	1270	-1.645276864	5.1347E-106	1.0646E-103
TR12810-c1_g1	43	135	-1.650550842	9.18483E-13	2.11019E-11
TR16039-c0_g1	43	135	-1.650550842	9.18483E-13	2.11019E-11
TR10563-c0_g1	7	22	-1.652076697	0.003915043	0.019226144
TR11843-c0_g1	7	22	-1.652076697	0.003915043	0.019226144
TR21451-c0_g1	7	22	-1.652076697	0.003915043	0.019226144
TR3674-c0_g1	7	22	-1.652076697	0.003915043	0.019226144
TR9234-c0_g1	7	22	-1.652076697	0.003915043	0.019226144
TR15437-c5_g1	57	180	-1.658963082	1.26565E-16	3.77505E-15
TR11798-c0_g1	25	79	-1.659924558	4.12975E-08	5.8607E-07
TR572-c4_g1	58	184	-1.665580961	4.79491E-17	1.46618E-15
TR21504-c2_g2	40	127	-1.666756592	3.05969E-12	6.79983E-11
TR10786-c0_g2	17	54	-1.667424661	5.38287E-06	5.49469E-05
TR12918-c0_g2	16	51	-1.672425342	9.40699E-06	9.24992E-05
TR24280-c0_g1	30	96	-1.678071905	1.10895E-09	1.89865E-08
TR4129-c0_g1	103	330	-1.679821687	1.22565E-29	6.9459E-28
TR25479-c0_g2	9	29	-1.688055994	0.000772259	0.004880828
TR299-c0_g1	9	29	-1.688055994	0.000772259	0.004880828
TR11839-c2_g1	39	126	-1.691877705	2.21311E-12	4.96383E-11
TR16222-c0_g1	16	52	-1.700439718	6.02015E-06	6.12511E-05
TR22426-c0_g1	16	52	-1.700439718	6.02015E-06	6.12511E-05
TR27762-c0_g1	8	26	-1.700439718	0.001373799	0.008015239
TR9461-c0_g1	8	26	-1.700439718	0.001373799	0.008015239
TR18966-c0_g1	44	144	-1.710493383	3.9579E-14	1.00884E-12
TR11215-c3_g1	40	131	-1.711494907	5.36095E-13	1.25197E-11
TR13439-c0_g1	7	23	-1.716207034	0.002452562	0.013005865
TR19705-c0_g1	7	23	-1.716207034	0.002452562	0.013005865
TR8753-c0_g2	7	23	-1.716207034	0.002452562	0.013005865
TR3829-c0_g1	10	33	-1.722466024	0.000275267	0.001963787
TR8287-c2_g1	49	162	-1.725140159	7.11064E-16	2.04206E-14
TR20298-c0_g1	16	53	-1.727920455	3.83794E-06	4.02034E-05
TR6438-c0_g1	16	53	-1.727920455	3.83794E-06	4.02034E-05
TR2626-c8_g1	88	292	-1.73039294	1.86765E-27	9.74006E-26
TR922-c0_g1	36	120	-1.736965594	3.01577E-12	6.711E-11
TR1059-c0_g1	15	50	-1.736965594	6.68501E-06	6.77722E-05
TR10714-c0_g1	9	30	-1.736965594	0.000486017	0.003226446
TR13226-c0_g1	9	30	-1.736965594	0.000486017	0.003226446
TR23370-c0_g1	9	30	-1.736965594	0.000486017	0.003226446
TR14516-c0_g1	6	20	-1.736965594	0.004396589	0.021253291
TR16332-c0_g1	6	20	-1.736965594	0.004396589	0.021253291

TR19535-c0_g1	6	20	-1.736965594	0.004396589	0.021253291
TR4005-c0_g1	17	57	-1.745427173	1.40348E-06	1.57916E-05
TR18966-c0_g2	45	151	-1.746551643	3.95805E-15	1.08887E-13
TR9467-c0_g1	14	47	-1.74723393	1.16516E-05	0.000113067
TR489-c12_g1	107	360	-1.75038611	5.74108E-34	3.84275E-32
TR11751-c0_g1	38	128	-1.752072487	4.20398E-13	9.92683E-12
TR15889-c0_g1	8	27	-1.754887502	0.000859935	0.005276083
TR16774-c0_g1	8	27	-1.754887502	0.000859935	0.005276083
TR676-c0_g1	8	27	-1.754887502	0.000859935	0.005276083
TR10975-c0_g1	29	98	-1.756728849	2.09537E-10	3.87219E-09
TR14986-c1_g1	13	44	-1.7589919	2.03215E-05	0.000188279
TR17558-c0_g1	36	122	-1.760812336	1.23824E-12	2.83334E-11
TR25331-c0_g1	12	41	-1.772589504	3.54656E-05	0.000314268
TR27019-c0_g1	12	41	-1.772589504	3.54656E-05	0.000314268
TR12123-c0_g3	7	24	-1.777607579	0.001525	0.008759127
TR21953-c0_g1	7	24	-1.777607579	0.001525	0.008759127
TR23627-c0_g1	7	24	-1.777607579	0.001525	0.008759127
TR8284-c0_g1	7	24	-1.777607579	0.001525	0.008759127
TR2127-c0_g2	44	151	-1.778973121	1.78111E-15	4.99689E-14
TR16349-c0_g1	18	62	-1.784271309	3.25652E-07	4.10112E-06
TR10741-c0_g2	9	31	-1.784271309	0.000304052	0.002148438
TR24931-c0_g1	9	31	-1.784271309	0.000304052	0.002148438
TR25331-c0_g2	11	38	-1.788495895	6.19334E-05	0.000514387
TR13907-c0_g1	13	45	-1.791413378	1.28003E-05	0.000122673
TR7155-c0_g1	10	35	-1.807354922	0.000108212	0.000852525
TR27762-c0_g3	8	28	-1.807354922	0.000534836	0.003515818
TR21312-c0_g1	6	21	-1.807354922	0.002710859	0.014277628
TR23637-c0_g1	6	21	-1.807354922	0.002710859	0.014277628
TR25876-c0_g2	6	21	-1.807354922	0.002710859	0.014277628
TR3634-c0_g2	6	21	-1.807354922	0.002710859	0.014277628
TR17965-c0_g2	19	67	-1.818161677	7.52861E-08	1.02315E-06
TR6628-c0_g1	153	541	-1.822096941	6.77466E-53	6.93845E-51
TR22807-c0_g1	13	46	-1.823122238	8.0297E-06	7.9787E-05
TR20778-c0_g1	22	78	-1.8259706	5.89865E-09	9.23433E-08
TR5607-c0_g2	11	39	-1.8259706	3.87162E-05	0.000339292
TR2583-c1_g1	54	192	-1.830074999	5.97244E-20	2.11982E-18
TR7575-c0_g1	32	114	-1.832890014	1.74345E-12	3.94161E-11
TR12123-c0_g1	7	25	-1.836501268	0.000941696	0.005738959
TR10078-c0_g1	213	765	-1.844606317	2.96526E-75	4.54174E-73
TR8301-c0_g1	20	72	-1.847996907	1.73556E-08	2.56804E-07
TR12918-c0_g1	10	36	-1.847996907	6.73289E-05	0.000554056
TR14888-c0_g1	5	18	-1.847996907	0.004829757	0.023182455
TR16215-c0_g1	5	18	-1.847996907	0.004829757	0.023182455
TR26249-c0_g1	5	18	-1.847996907	0.004829757	0.023182455

TR8191-c0_g2	5	18	-1.847996907	0.004829757	0.023182455
TR16774-c0_g2	23	83	-1.851477475	1.36621E-09	2.30889E-08
TR1624-c0_g1	18	65	-1.852442812	8.13589E-08	1.1048E-06
TR15918-c0_g1	24	87	-1.857980995	5.02654E-10	8.99556E-09
TR27536-c0_g3	16	58	-1.857980995	3.83172E-07	4.74813E-06
TR14555-c0_g1	8	29	-1.857980995	0.000330641	0.002315215
TR14664-c0_g1	8	29	-1.857980995	0.000330641	0.002315215
TR22326-c0_g1	8	29	-1.857980995	0.000330641	0.002315215
TR1624-c0_g2	22	80	-1.862496476	2.33615E-09	3.83006E-08
TR20299-c0_g1	54	197	-1.867164317	6.08958E-21	2.30069E-19
TR17258-c0_g1	12	44	-1.874469118	8.65854E-06	8.53867E-05
TR5607-c0_g1	12	44	-1.874469118	8.65854E-06	8.53867E-05
TR23553-c0_g1	9	33	-1.874469118	0.000117037	0.000917795
TR465-c0_g1	6	22	-1.874469118	0.001658969	0.009342828
TR5460-c0_g2	6	22	-1.874469118	0.001658969	0.009342828
TR13734-c0_g1	65	239	-1.878498995	2.96783E-25	1.35274E-23
TR17155-c0_g1	53	195	-1.879409859	6.51207E-21	2.44942E-19
TR26926-c0_g1	65	240	-1.884522783	1.8729E-25	8.69992E-24
TR22326-c0_g2	10	37	-1.887525271	4.16915E-05	0.000359802
TR4836-c0_g1	10	37	-1.887525271	4.16915E-05	0.000359802
TR8157-c0_g1	10	37	-1.887525271	4.16915E-05	0.000359802
TR17993-c0_g3	21	78	-1.893084796	2.49797E-09	4.08353E-08
TR14664-c0_g2	7	26	-1.893084796	0.000577759	0.003773598
TR5460-c0_g1	7	26	-1.893084796	0.000577759	0.003773598
TR5887-c0_g2	7	26	-1.893084796	0.000577759	0.003773598
TR2832-c0_g1	18	67	-1.896164189	3.18097E-08	4.60262E-07
TR11751-c0_g2	29	108	-1.896906507	2.14919E-12	4.82683E-11
TR7745-c1_g1	69	257	-1.897100092	2.28915E-27	1.17935E-25
TR56-c0_g2	22	82	-1.898120386	9.16547E-10	1.59168E-08
TR6431-c0_g1	63	236	-1.905363126	2.11914E-25	9.79027E-24
TR7598-c1_g1	176	660	-1.906890596	4.9313E-68	6.70709E-66
TR6545-c0_g2	40	150	-1.906890596	9.79038E-17	2.93562E-15
TR12974-c0_g1	12	45	-1.906890596	5.36751E-06	5.48736E-05
TR3903-c3_g1	174	654	-1.91020333	1.39537E-67	1.88279E-65
TR10786-c0_g1	17	64	-1.912537159	5.42798E-08	7.6141E-07
TR1192-c0_g1	9	34	-1.91753784	7.20537E-05	0.000590081
TR13863-c0_g1	9	34	-1.91753784	7.20537E-05	0.000590081
TR15956-c0_g1	9	34	-1.91753784	7.20537E-05	0.000590081
TR20281-c0_g1	62	235	-1.922320636	1.40951E-25	6.60155E-24
TR10079-c0_g1	25	95	-1.925999419	2.84255E-11	5.76009E-10
TR18972-c0_g1	10	38	-1.925999419	2.56988E-05	0.000233519
TR3190-c0_g1	10	38	-1.925999419	2.56988E-05	0.000233519
TR14928-c0_g1	5	19	-1.925999419	0.002921433	0.015198354
TR6482-c0_g1	184	701	-1.929708678	2.95937E-73	4.37506E-71

TR2625-c2_g1	78	298	-1.933766302	3.14789E-32	1.93907E-30
TR13641-c0_g1	54	207	-1.938599455	5.87731E-23	2.43119E-21
TR3127-c0_g3	6	23	-1.938599455	0.001008237	0.006116087
TR5124-c0_g1	6	23	-1.938599455	0.001008237	0.006116087
TR345-c0_g1	20	77	-1.944858446	1.6466E-09	2.74723E-08
TR21760-c0_g1	14	54	-1.94753258	4.33458E-07	5.33625E-06
TR3234-c0_g1	14	54	-1.94753258	4.33458E-07	5.33625E-06
TR8256-c0_g3	14	54	-1.94753258	4.33458E-07	5.33625E-06
TR3983-c0_g1	53	205	-1.951559645	6.17453E-23	2.54794E-21
TR20343-c0_g1	16	62	-1.95419631	5.72565E-08	8.00523E-07
TR12118-c0_g1	9	35	-1.959358016	4.41478E-05	0.000379652
TR9369-c1_g2	9	35	-1.959358016	4.41478E-05	0.000379652
TR13815-c0_g1	66	258	-1.966833136	1.01995E-28	5.50493E-27
TR558-c3_g1	76	298	-1.971241007	5.29579E-33	3.39757E-31
TR15636-c0_g1	29	114	-1.974909019	1.26336E-13	3.12646E-12
TR24288-c0_g1	15	59	-1.975752454	9.70756E-08	1.30881E-06
TR12243-c0_g1	19	75	-1.980891177	1.72318E-09	2.86377E-08
TR14901-c1_g1	23	91	-1.984232684	3.125E-11	6.30987E-10
TR10054-c5_g1	14	56	-2	1.64198E-07	2.15233E-06
TR267-c1_g1	10	40	-2	9.63981E-06	9.46244E-05
TR25166-c0_g1	9	36	-2	2.6927E-05	0.000244027
TR2988-c0_g1	6	24	-2	0.000608844	0.003949748
TR797-c0_g1	6	24	-2	0.000608844	0.003949748
TR15975-c0_g1	5	20	-2	0.001754078	0.009745637
TR18109-c0_g1	5	20	-2	0.001754078	0.009745637
TR18411-c0_g1	5	20	-2	0.001754078	0.009745637
TR20861-c0_g2	5	20	-2	0.001754078	0.009745637
TR22060-c0_g1	5	20	-2	0.001754078	0.009745637
TR25857-c0_g1	5	20	-2	0.001754078	0.009745637
TR27289-c0_g1	5	20	-2	0.001754078	0.009745637
TR6649-c0_g1	5	20	-2	0.001754078	0.009745637
TR7462-c0_g2	5	20	-2	0.001754078	0.009745637
TR26281-c1_g1	40	161	-2.008988783	5.39085E-19	1.8221E-17
TR472-c4_g1	66	267	-2.016301812	1.42049E-30	8.35649E-29
TR19757-c0_g1	52	211	-2.020659471	1.43514E-24	6.35396E-23
TR10248-c0_g1	15	61	-2.023846742	3.66263E-08	5.25038E-07
TR499-c0_g1	24	98	-2.029747343	2.70086E-12	6.01811E-11
TR14739-c0_g1	9	37	-2.039528364	1.63528E-05	0.000153517
TR4573-c0_g1	8	33	-2.044394119	4.57794E-05	0.000392689
TR2731-c1_g1	44	182	-2.048363022	9.23739E-22	3.63537E-20
TR6634-c0_g1	14	58	-2.050626073	6.14662E-08	8.52371E-07
TR21056-c0_g1	7	29	-2.050626073	0.00012889	0.000999222
TR21056-c0_g2	7	29	-2.050626073	0.00012889	0.000999222
TR5619-c0_g1	7	29	-2.050626073	0.00012889	0.000999222

TR8071-c0_g1	34	141	-2.052088511	3.00788E-17	9.34881E-16
TR13769-c1_g1	40	166	-2.053111336	4.84122E-20	1.73644E-18
TR567-c1_g1	13	54	-2.054447784	1.68947E-07	2.20947E-06
TR16397-c0_g1	12	50	-2.058893689	4.6538E-07	5.69214E-06
TR15260-c0_g1	6	25	-2.058893689	0.00036549	0.002533146
TR22033-c0_g1	6	25	-2.058893689	0.00036549	0.002533146
TR23606-c0_g1	6	25	-2.058893689	0.00036549	0.002533146
TR27536-c0_g1	6	25	-2.058893689	0.00036549	0.002533146
TR8914-c0_g1	6	25	-2.058893689	0.00036549	0.002533146
TR1449-c0_g2	11	46	-2.064130337	1.28514E-06	1.4537E-05
TR23653-c0_g1	11	46	-2.064130337	1.28514E-06	1.4537E-05
TR2577-c3_g1	61	256	-2.069262662	2.58824E-30	1.50697E-28
TR3113-c0_g1	10	42	-2.070389328	3.55916E-06	3.78191E-05
TR3172-c0_g1	5	21	-2.070389328	0.001046083	0.006306663
TR9515-c0_g1	5	21	-2.070389328	0.001046083	0.006306663
TR21631-c0_g2	9	38	-2.078002512	9.89047E-06	9.69729E-05
TR7593-c0_g1	16	68	-2.087462841	3.04578E-09	4.94578E-08
TR25166-c0_g2	8	34	-2.087462841	2.75951E-05	0.000249019
TR3946-c0_g1	8	34	-2.087462841	2.75951E-05	0.000249019
TR4573-c0_g2	8	34	-2.087462841	2.75951E-05	0.000249019
TR18983-c0_g1	4	17	-2.087462841	0.00303182	0.015332054
TR22548-c0_g1	4	17	-2.087462841	0.00303182	0.015332054
TR3250-c0_g1	4	17	-2.087462841	0.00303182	0.015332054
TR3257-c0_g1	4	17	-2.087462841	0.00303182	0.015332054
TR3297-c0_g1	4	17	-2.087462841	0.00303182	0.015332054
TR7516-c0_g1	4	17	-2.087462841	0.00303182	0.015332054
TR9296-c0_g1	4	17	-2.087462841	0.00303182	0.015332054
TR19767-c0_g1	6	26	-2.115477217	0.0002182	0.001608014
TR9207-c0_g1	6	26	-2.115477217	0.0002182	0.001608014
TR21-c0_g1	11	48	-2.125530882	4.70183E-07	5.74675E-06
TR26940-c0_g1	16	70	-2.129283017	1.12334E-09	1.92136E-08
TR10134-c0_g1	5	22	-2.137503524	0.000620009	0.004001886
TR12974-c0_g2	5	22	-2.137503524	0.000620009	0.004001886
TR14621-c0_g1	7	31	-2.146841388	4.62242E-05	0.000395507
TR4942-c0_g1	7	31	-2.146841388	4.62242E-05	0.000395507
TR9163-c0_g1	7	31	-2.146841388	4.62242E-05	0.000395507
TR22991-c0_g1	16	71	-2.14974712	6.79925E-10	1.19171E-08
TR7650-c0_g1	62	279	-2.169925001	8.61837E-35	5.93213E-33
TR14705-c0_g1	12	54	-2.169925001	6.2132E-08	8.58802E-07
TR24153-c0_g2	12	54	-2.169925001	6.2132E-08	8.58802E-07
TR5153-c0_g1	12	54	-2.169925001	6.2132E-08	8.58802E-07
TR26115-c0_g1	10	45	-2.169925001	7.77446E-07	9.16617E-06
TR5139-c1_g1	6	27	-2.169925001	0.000129603	0.001002921
TR11553-c0_g1	4	18	-2.169925001	0.001778689	0.009850183

TR13299-c0_g1	4	18	-2.169925001	0.001778689	0.009850183
TR13362-c0_g1	4	18	-2.169925001	0.001778689	0.009850183
TR22548-c0_g2	4	18	-2.169925001	0.001778689	0.009850183
TR26121-c0_g1	4	18	-2.169925001	0.001778689	0.009850183
TR3851-c0_g1	4	18	-2.169925001	0.001778689	0.009850183
TR5195-c0_g1	4	18	-2.169925001	0.001778689	0.009850183
TR7015-c0_g2	4	18	-2.169925001	0.001778689	0.009850183
TR13317-c0_g1	64	290	-2.17990909	2.73029E-36	1.95859E-34
TR21-c0_g2	11	50	-2.184424571	1.69904E-07	2.22029E-06
TR19918-c0_g1	104	473	-2.185256655	2.94428E-58	3.47615E-56
TR4701-c0_g1	47	214	-2.186878135	2.55931E-27	1.31059E-25
TR17258-c0_g2	9	41	-2.187627003	2.13915E-06	2.3373E-05
TR16433-c0_g1	108	493	-2.190556334	8.07306E-61	9.80377E-59
TR22067-c0_g1	7	32	-2.192645078	2.74975E-05	0.000248667
TR8473-c0_g1	40	183	-2.193771743	1.10848E-23	4.75901E-22
TR913-c0_g1	12	55	-2.196397213	3.72944E-08	5.33266E-07
TR2653-c0_g4	15	69	-2.201633861	6.7027E-10	1.176E-08
TR12991-c0_g2	5	23	-2.201633861	0.000365399	0.002533146
TR19400-c0_g1	5	23	-2.201633861	0.000365399	0.002533146
TR573-c0_g1	5	23	-2.201633861	0.000365399	0.002533146
TR8284-c0_g2	5	23	-2.201633861	0.000365399	0.002533146
TR8813-c0_g2	11	51	-2.212993723	1.01691E-07	1.36563E-06
TR11851-c0_g1	12	56	-2.222392421	2.23274E-08	3.27519E-07
TR5319-c0_g2	9	42	-2.222392421	1.27514E-06	1.44431E-05
TR1449-c0_g1	10	47	-2.232660757	2.77488E-07	3.50756E-06
TR12200-c0_g1	14	66	-2.237039197	1.0875E-09	1.86569E-08
TR2724-c0_g2	7	33	-2.237039197	1.62906E-05	0.000153187
TR3040-c0_g1	7	33	-2.237039197	1.62906E-05	0.000153187
TR4942-c0_g2	7	33	-2.237039197	1.62906E-05	0.000153187
TR840-c0_g1	7	33	-2.237039197	1.62906E-05	0.000153187
TR17965-c0_g1	47	222	-2.239827015	4.60017E-29	2.53926E-27
TR12551-c0_g1	12	57	-2.247927513	1.33333E-08	1.99898E-07
TR16788-c0_g1	12	57	-2.247927513	1.33333E-08	1.99898E-07
TR15620-c0_g1	8	38	-2.247927513	3.49956E-06	3.72089E-05
TR13221-c0_g1	4	19	-2.247927513	0.001036953	0.006258281
TR17985-c0_g1	4	19	-2.247927513	0.001036953	0.006258281
TR3820-c0_g1	4	19	-2.247927513	0.001036953	0.006258281
TR66-c0_g1	4	19	-2.247927513	0.001036953	0.006258281
TR8776-c0_g3	4	19	-2.247927513	0.001036953	0.006258281
TR3845-c0_g1	17	81	-2.252387162	1.19159E-11	2.50416E-10
TR17687-c0_g1	9	43	-2.256339753	7.57666E-07	8.93915E-06
TR27361-c0_g2	9	43	-2.256339753	7.57666E-07	8.93915E-06
TR9411-c0_g2	10	48	-2.263034406	1.65049E-07	2.16016E-06
TR11961-c0_g1	5	24	-2.263034406	0.000214228	0.001581488

TR13302-c0_g1	5	24	-2.263034406	0.000214228	0.001581488
TR19722-c0_g1	5	24	-2.263034406	0.000214228	0.001581488
TR5579-c0_g1	5	24	-2.263034406	0.000214228	0.001581488
TR10047-c0_g1	24	116	-2.273018494	3.35801E-16	9.80938E-15
TR10671-c0_g1	6	29	-2.273018494	4.50895E-05	0.000387358
TR624-c0_g1	6	29	-2.273018494	4.50895E-05	0.000387358
TR24028-c0_g1	7	34	-2.280107919	9.61401E-06	9.44257E-05
TR24153-c0_g1	9	44	-2.289506617	4.48812E-07	5.50533E-06
TR9411-c0_g1	10	49	-2.292781749	9.78977E-08	1.31677E-06
TR11851-c0_g3	13	64	-2.299560282	1.04212E-09	1.79325E-08
TR56-c0_g1	52	259	-2.31636857	6.17409E-35	4.2844E-33
TR11621-c0_g1	13	65	-2.321928095	6.18531E-10	1.09086E-08
TR21235-c0_g2	8	40	-2.321928095	1.21998E-06	1.38849E-05
TR22285-c0_g1	8	40	-2.321928095	1.21998E-06	1.38849E-05
TR2724-c0_g1	8	40	-2.321928095	1.21998E-06	1.38849E-05
TR18853-c0_g1	6	30	-2.321928095	2.64269E-05	0.000239623
TR975-c0_g1	6	30	-2.321928095	2.64269E-05	0.000239623
TR12350-c0_g2	5	25	-2.321928095	0.000124999	0.000971717
TR22755-c0_g1	5	25	-2.321928095	0.000124999	0.000971717
TR3886-c0_g1	5	25	-2.321928095	0.000124999	0.000971717
TR12576-c0_g1	4	20	-2.321928095	0.000601108	0.003909577
TR12301-c0_g1	3	15	-2.321928095	0.002964202	0.015332054
TR21640-c0_g1	3	15	-2.321928095	0.002964202	0.015332054
TR21640-c0_g2	3	15	-2.321928095	0.002964202	0.015332054
TR21988-c0_g1	3	15	-2.321928095	0.002964202	0.015332054
TR9150-c0_g1	3	15	-2.321928095	0.002964202	0.015332054
TR918-c0_g1	3	15	-2.321928095	0.002964202	0.015332054
TR9836-c0_g2	13	66	-2.343954401	3.6637E-10	6.65468E-09
TR22354-c0_g1	12	61	-2.345774837	1.6563E-09	2.76072E-08
TR13327-c0_g1	23	117	-2.346802764	6.75839E-17	2.04088E-15
TR8813-c0_g1	10	51	-2.350497247	3.41712E-08	4.90671E-07
TR24234-c0_g1	8	41	-2.357552005	7.16931E-07	8.51172E-06
TR17615-c0_g1	15	77	-2.359895945	1.06957E-11	2.2533E-10
TR15933-c0_g1	7	36	-2.362570079	3.31274E-06	3.53331E-05
TR807-c0_g1	7	36	-2.362570079	3.31274E-06	3.53331E-05
TR4868-c0_g1	15	78	-2.378511623	6.32449E-12	1.36974E-10
TR10073-c0_g1	5	26	-2.378511623	7.26135E-05	0.000594093
TR2729-c0_g1	14	73	-2.382469637	2.84011E-11	5.76009E-10
TR17603-c0_g1	17	89	-2.38827059	1.86409E-13	4.54039E-12
TR16788-c0_g3	12	63	-2.392317423	5.76223E-10	1.01835E-08
TR12676-c0_g2	4	21	-2.392317423	0.000346672	0.00241751
TR20861-c0_g1	4	21	-2.392317423	0.000346672	0.00241751
TR6281-c0_g1	4	21	-2.392317423	0.000346672	0.00241751
TR9630-c0_g2	4	21	-2.392317423	0.000346672	0.00241751

TR12692-c0_g1	3	16	-2.415037499	0.00169017	0.009436823
TR13320-c0_g1	3	16	-2.415037499	0.00169017	0.009436823
TR8408-c0_g1	3	16	-2.415037499	0.00169017	0.009436823
TR9188-c0_g1	3	16	-2.415037499	0.00169017	0.009436823
TR470-c6_g1	152	811	-2.415630591	8.7964E-111	1.9422E-108
TR11851-c0_g2	14	75	-2.421463768	9.85863E-12	2.0873E-10
TR16788-c0_g2	14	75	-2.421463768	9.85863E-12	2.0873E-10
TR3409-c0_g1	22	118	-2.423211431	1.29872E-17	4.09648E-16
TR2327-c0_g1	19	102	-2.424497829	1.9288E-15	5.39344E-14
TR13301-c0_g1	21	113	-2.42786154	5.76224E-17	1.74316E-15
TR26990-c1_g1	18	97	-2.429987841	8.5836E-15	2.28376E-13
TR2609-c7_g1	135	728	-2.430979043	2.3884E-100	4.7772E-98
TR7059-c0_g2	16	87	-2.442943496	1.70463E-13	4.16392E-12
TR4868-c0_g2	15	82	-2.450661409	7.6081E-13	1.75744E-11
TR21235-c0_g1	6	33	-2.459431619	5.20371E-06	5.33594E-05
TR11676-c0_g2	4	22	-2.459431619	0.000199008	0.001477466
TR7015-c0_g1	4	22	-2.459431619	0.000199008	0.001477466
TR7059-c0_g1	15	83	-2.468148836	4.4635E-13	1.05104E-11
TR9836-c0_g1	15	83	-2.468148836	4.4635E-13	1.05104E-11
TR883-c0_g1	13	72	-2.469485283	1.52092E-11	3.14954E-10
TR4024-c0_g1	53	295	-2.47665069	1.37067E-42	1.10968E-40
TR10567-c0_g1	7	39	-2.478047297	6.54236E-07	7.87182E-06
TR25501-c0_g1	7	39	-2.478047297	6.54236E-07	7.87182E-06
TR27484-c0_g1	7	39	-2.478047297	6.54236E-07	7.87182E-06
TR15680-c0_g1	5	28	-2.485426827	2.42138E-05	0.000220732
TR4137-c0_g1	5	28	-2.485426827	2.42138E-05	0.000220732
TR6653-c0_g1	13	73	-2.489384841	8.89547E-12	1.89993E-10
TR11621-c0_g2	11	62	-2.494764692	3.05712E-10	5.60076E-09
TR10671-c0_g2	6	34	-2.502500341	3.00737E-06	3.23398E-05
TR12602-c0_g1	6	34	-2.502500341	3.00737E-06	3.23398E-05
TR807-c0_g2	6	34	-2.502500341	3.00737E-06	3.23398E-05
TR25479-c0_g1	3	17	-2.502500341	0.00095864	0.00583327
TR4218-c0_g1	7	40	-2.514573173	3.78831E-07	4.71841E-06
TR4896-c0_g1	12	69	-2.523561956	2.31822E-11	4.73712E-10
TR16532-c0_g1	8	46	-2.523561956	4.82148E-08	6.80262E-07
TR16829-c0_g2	4	23	-2.523561956	0.000113761	0.000894168
TR2638-c0_g2	4	23	-2.523561956	0.000113761	0.000894168
TR3797-c0_g1	4	23	-2.523561956	0.000113761	0.000894168
TR11241-c0_g1	41	236	-2.525091045	3.93795E-35	2.74386E-33
TR6486-c0_g1	21	121	-2.526545814	8.16141E-19	2.73678E-17
TR6502-c0_g1	52	301	-2.533179959	1.74819E-44	1.47136E-42
TR5840-c0_g1	5	29	-2.5360529	1.39076E-05	0.00013291
TR5221-c0_g1	12	70	-2.544320516	1.34885E-11	2.8235E-10
TR7566-c0_g1	12	70	-2.544320516	1.34885E-11	2.8235E-10

TR14476-c0_g1	45	263	-2.547065893	2.5891E-39	1.94771E-37
TR16582-c0_g1	11	66	-2.584962501	3.48374E-11	6.9927E-10
TR3169-c0_g4	9	54	-2.584962501	2.07173E-09	3.41963E-08
TR13273-c0_g1	8	48	-2.584962501	1.61032E-08	2.38896E-07
TR19567-c0_g2	8	48	-2.584962501	1.61032E-08	2.38896E-07
TR10330-c0_g1	7	42	-2.584962501	1.26051E-07	1.66903E-06
TR8906-c0_g2	7	42	-2.584962501	1.26051E-07	1.66903E-06
TR24297-c0_g1	6	36	-2.584962501	9.9572E-07	1.15711E-05
TR22958-c0_g1	4	24	-2.584962501	6.47821E-05	0.000535691
TR16368-c0_g1	3	18	-2.584962501	0.000541208	0.003549864
TR24117-c0_g3	3	18	-2.584962501	0.000541208	0.003549864
TR784-c0_g1	3	18	-2.584962501	0.000541208	0.003549864
TR10649-c0_g1	2	12	-2.584962501	0.004733192	0.022809107
TR10649-c0_g2	2	12	-2.584962501	0.004733192	0.022809107
TR19864-c0_g1	2	12	-2.584962501	0.004733192	0.022809107
TR5617-c0_g1	2	12	-2.584962501	0.004733192	0.022809107
TR9592-c0_g1	19	115	-2.597562538	1.82305E-18	5.96043E-17
TR17763-c0_g1	16	98	-2.614709844	4.5629E-16	1.32607E-14
TR479-c0_g1	13	81	-2.639410285	1.15415E-13	2.86035E-12
TR8299-c1_g2	35	220	-2.652076697	1.42035E-34	9.73703E-33
TR25922-c0_g2	10	63	-2.655351829	5.14817E-11	1.01069E-09
TR946-c0_g1	25	158	-2.659924558	2.25533E-25	1.03631E-23
TR8906-c0_g1	6	38	-2.662965013	3.26229E-07	4.10534E-06
TR15119-c0_g2	3	19	-2.662965013	0.000304296	0.002148438
TR27356-c0_g2	3	19	-2.662965013	0.000304296	0.002148438
TR9630-c0_g1	3	19	-2.662965013	0.000304296	0.002148438
TR11261-c0_g1	19	123	-2.694586992	2.30756E-20	8.40076E-19
TR14676-c0_g2	6	39	-2.700439718	1.86067E-07	2.4148E-06
TR13371-c0_g1	2	13	-2.700439718	0.002616324	0.013822549
TR25092-c0_g1	2	13	-2.700439718	0.002616324	0.013822549
TR25092-c0_g2	2	13	-2.700439718	0.002616324	0.013822549
TR23591-c0_g1	27	176	-2.704544116	1.51741E-28	8.1639E-27
TR530-c0_g1	21	137	-2.70571466	1.36771E-22	5.58963E-21
TR26862-c0_g1	8	53	-2.727920455	1.00233E-09	1.72829E-08
TR1985-c0_g1	3	20	-2.736965594	0.000170476	0.001278646
TR27610-c0_g1	20	134	-2.744161096	1.95707E-22	7.90328E-21
TR25922-c0_g1	10	67	-2.744161096	5.58346E-12	1.217E-10
TR10744-c0_g1	23	155	-2.752562449	8.97524E-26	4.23864E-24
TR10343-c0_g1	4	27	-2.754887502	1.17299E-05	0.000113567
TR2638-c0_g1	4	27	-2.754887502	1.17299E-05	0.000113567
TR3959-c0_g1	4	27	-2.754887502	1.17299E-05	0.000113567
TR931-c0_g1	4	27	-2.754887502	1.17299E-05	0.000113567
TR8524-c0_g1	26	176	-2.7589919	4.152E-29	2.29933E-27
TR6777-c0_g1	23	156	-2.761840263	5.16304E-26	2.45878E-24

TR13819-c0_g1	5	34	-2.765534746	8.31855E-07	9.76402E-06
TR10330-c0_g2	6	41	-2.772589504	6.01415E-08	8.35363E-07
TR10744-c0_g2	22	151	-2.778973121	2.20847E-25	1.01753E-23
TR14903-c0_g1	15	103	-2.779609932	8.01435E-18	2.54681E-16
TR3749-c0_g1	10	69	-2.786596362	1.8236E-12	4.10645E-11
TR4006-c0_g1	11	76	-2.788495895	1.37444E-13	3.38658E-12
TR268-c0_g1	15	105	-2.807354922	2.62665E-18	8.52225E-17
TR2936-c0_g1	12	84	-2.807354922	5.94214E-15	1.62158E-13
TR17924-c0_g1	3	21	-2.807354922	9.5202E-05	0.000757753
TR19388-c0_g1	3	21	-2.807354922	9.5202E-05	0.000757753
TR9712-c0_g1	3	21	-2.807354922	9.5202E-05	0.000757753
TR10152-c0_g1	2	14	-2.807354922	0.001440675	0.008350968
TR13731-c0_g5	2	14	-2.807354922	0.001440675	0.008350968
TR19484-c0_g1	2	14	-2.807354922	0.001440675	0.008350968
TR3058-c0_g1	2	14	-2.807354922	0.001440675	0.008350968
TR6777-c0_g2	22	156	-2.8259706	1.36924E-26	6.7086E-25
TR795-c0_g1	11	78	-2.8259706	4.47476E-14	1.13547E-12
TR11219-c12_g2	335	2408	-2.845602391	0	0
TR13790-c0_g5	5	36	-2.847996907	2.65096E-07	3.36845E-06
TR840-c0_g2	5	36	-2.847996907	2.65096E-07	3.36845E-06
TR16335-c0_g1	4	29	-2.857980995	3.70326E-06	3.90573E-05
TR13818-c14_g1	397	2891	-2.864357696	0	0
TR23633-c0_g1	8	59	-2.882643049	3.39046E-11	6.82965E-10
TR3410-c0_g1	10	74	-2.887525271	1.08836E-13	2.70519E-12
TR24291-c0_g1	14	104	-2.893084796	1.18262E-18	3.91932E-17
TR4183-c0_g1	9	67	-2.896164189	1.44187E-12	3.28163E-11
TR9507-c0_g1	14	105	-2.906890596	6.72467E-19	2.26392E-17
TR22375-c0_g1	2	15	-2.906890596	0.000790819	0.004964909
TR23307-c0_g1	2	15	-2.906890596	0.000790819	0.004964909
TR27356-c0_g1	2	15	-2.906890596	0.000790819	0.004964909
TR5469-c0_g1	2	15	-2.906890596	0.000790819	0.004964909
TR6869-c0_g1	2	15	-2.906890596	0.000790819	0.004964909
TR15485-c0_g1	9	68	-2.91753784	8.16556E-13	1.88365E-11
TR15232-c1_g1	50	380	-2.925999419	2.00912E-64	2.5877E-62
TR9799-c0_g1	5	38	-2.925999419	8.38257E-08	1.13648E-06
TR10353-c0_g3	3	23	-2.938599455	2.94499E-05	0.000263797
TR25252-c0_g1	3	23	-2.938599455	2.94499E-05	0.000263797
TR3813-c0_g1	3	23	-2.938599455	2.94499E-05	0.000263797
TR22394-c0_g1	14	108	-2.94753258	1.23083E-19	4.33245E-18
TR14498-c0_g1	19	147	-2.951744831	3.5048E-26	1.70246E-24
TR19723-c0_g1	4	31	-2.95419631	1.15886E-06	1.33212E-05
TR5220-c0_g1	4	31	-2.95419631	1.15886E-06	1.33212E-05
TR8520-c0_g1	4	31	-2.95419631	1.15886E-06	1.33212E-05

TR3876-c1_g1	60	467	-2.960388144	1.2644E-79	2.02797E-77
TR26988-c3_g1	164	1298	-2.984522663	1.3859E-219	6.2005E-217
TR14639-c0_g1	35	278	-2.989658056	1.42918E-48	1.34988E-46
TR11262-c0_g1	3	24	-3	1.63231E-05	0.000153323
TR20707-c0_g1	3	24	-3	1.63231E-05	0.000153323
TR21317-c0_g1	2	16	-3	0.000432985	0.002943347
TR23771-c0_g1	2	16	-3	0.000432985	0.002943347
TR5674-c0_g1	2	16	-3	0.000432985	0.002943347
TR8442-c0_g1	2	16	-3	0.000432985	0.002943347
TR5810-c0_g1	24	193	-3.007494537	1.96269E-34	1.33473E-32
TR609-c1_g1	57	461	-3.015732926	5.44803E-80	8.99259E-78
TR8256-c0_g2	31	253	-3.028797264	6.88884E-45	5.91512E-43
TR8299-c1_g1	23	188	-3.031026896	8.05826E-34	5.33078E-32
TR21069-c1_g1	5	41	-3.03562391	1.47214E-08	2.19547E-07
TR13349-c2_g1	155	1305	-3.073709686	7.0892E-227	3.6523E-224
TR2989-c0_g1	16	135	-3.076815597	4.45637E-25	2.02038E-23
TR8774-c0_g1	9	76	-3.078002512	8.33231E-15	2.22736E-13
TR14639-c0_g2	32	271	-3.082149041	1.03448E-48	9.82541E-47
TR19405-c0_g2	4	34	-3.087462841	2.00139E-07	2.59149E-06
TR27356-c0_g3	2	17	-3.087462841	0.000236569	0.001729146
TR22256-c0_g1	9	77	-3.096861539	4.67878E-15	1.28299E-13
TR10424-c0_g1	5	43	-3.10433666	4.58432E-09	7.27727E-08
TR17576-c0_g1	3	26	-3.115477217	4.98654E-06	5.13494E-05
TR20304-c7_g1	147	1277	-3.118870465	5.306E-225	2.5774E-222
TR15232-c1_g2	13	113	-3.119739244	1.67065E-21	6.47001E-20
TR19803-c0_g1	7	61	-3.123382416	2.54259E-12	5.68781E-11
TR7797-c7_g1	147	1294	-3.137949557	3.2217E-229	1.7116E-226
TR4595-c0_g3	43	381	-3.147382433	5.10508E-69	7.05635E-67
TR6467-c0_g2	34	302	-3.150941898	3.87356E-55	4.3326E-53
TR18649-c0_g1	6	54	-3.169925001	3.33728E-11	6.7305E-10
TR4898-c0_g1	5	45	-3.169925001	1.42078E-09	2.39634E-08
TR4898-c0_g2	5	45	-3.169925001	1.42078E-09	2.39634E-08
TR16497-c0_g2	2	18	-3.169925001	0.000129036	0.000999443
TR24117-c0_g1	2	18	-3.169925001	0.000129036	0.000999443
TR906-c0_g1	92	829	-3.171666335	5.9812E-149	1.784E-146
TR19896-c0_g1	4	37	-3.209453366	3.41246E-08	4.90417E-07
TR25673-c0_g2	3	28	-3.222392421	1.51406E-06	1.69684E-05
TR8895-c1_g2	3	28	-3.222392421	1.51406E-06	1.69684E-05
TR2160-c1_g1	13	122	-3.230297619	9.1922E-24	3.96649E-22
TR5215-c0_g1	5	47	-3.232660757	4.38504E-10	7.89816E-09
TR6467-c0_g1	36	339	-3.235216462	4.42051E-63	5.567E-61
TR2114-c0_g1	7	66	-3.237039197	1.37767E-13	3.38962E-12
TR5812-c0_g1	4	38	-3.247927513	1.88791E-08	2.78377E-07
TR13933-c0_g1	2	19	-3.247927513	7.02867E-05	0.000577279

TR15119-c0_g3	2	19	-3.247927513	7.02867E-05	0.000577279
TR16497-c0_g3	2	19	-3.247927513	7.02867E-05	0.000577279
TR3119-c1_g1	11	105	-3.254813899	8.44211E-21	3.14752E-19
TR13736-c3_g3	9	86	-3.256339753	2.51538E-17	7.86117E-16
TR26375-c0_g1	46	441	-3.26107289	4.70446E-82	7.99821E-80
TR14778-c0_g1	19	183	-3.267772325	3.78908E-35	2.651E-33
TR5480-c0_g1	15	145	-3.273018494	3.10695E-28	1.66108E-26
TR7016-c0_g1	3	29	-3.273018494	8.32749E-07	9.76402E-06
TR8751-c0_g3	3	29	-3.273018494	8.32749E-07	9.76402E-06
TR27642-c0_g1	8	78	-3.285402219	5.75679E-16	1.66734E-14
TR5536-c0_g1	9	88	-3.289506617	7.8226E-18	2.49053E-16
TR636-c3_g1	290	2861	-3.302394692	0	0
TR18093-c0_g1	10	99	-3.307428525	5.96396E-20	2.11982E-18
TR7790-c3_g1	272	2710	-3.316614295	0	0
TR10016-c3_g1	66	658	-3.317549654	3.0207E-123	7.3366E-121
TR11165-c1_g1	14	140	-3.321928095	1.2161E-27	6.38127E-26
TR8525-c0_g1	10	100	-3.321928095	3.32396E-20	1.19982E-18
TR8072-c0_g1	4	40	-3.321928095	5.76181E-09	9.03676E-08
TR7016-c0_g2	3	30	-3.321928095	4.57548E-07	5.60038E-06
TR15119-c0_g1	2	20	-3.321928095	3.8245E-05	0.000335448
TR16497-c0_g1	2	20	-3.321928095	3.8245E-05	0.000335448
TR16497-c0_g4	2	20	-3.321928095	3.8245E-05	0.000335448
TR7795-c0_g1	2	20	-3.321928095	3.8245E-05	0.000335448
TR10962-c0_g1	1	10	-3.321928095	0.003594212	0.017762134
TR14172-c0_g1	1	10	-3.321928095	0.003594212	0.017762134
TR16850-c0_g1	1	10	-3.321928095	0.003594212	0.017762134
TR18509-c0_g1	1	10	-3.321928095	0.003594212	0.017762134
TR21457-c0_g1	1	10	-3.321928095	0.003594212	0.017762134
TR27333-c0_g1	1	10	-3.321928095	0.003594212	0.017762134
TR3894-c0_g1	1	10	-3.321928095	0.003594212	0.017762134
TR5121-c0_g1	1	10	-3.321928095	0.003594212	0.017762134
TR5358-c0_g1	1	10	-3.321928095	0.003594212	0.017762134
TR5469-c0_g2	1	10	-3.321928095	0.003594212	0.017762134
TR4595-c0_g5	32	322	-3.330916878	1.80607E-61	2.22504E-59
TR17568-c3_g1	73	735	-3.331775881	6.3088E-138	1.73E-135
TR11676-c0_g1	8	82	-3.357552005	5.51447E-17	1.67277E-15
TR10291-c1_g2	4	41	-3.357552005	3.17906E-09	5.14256E-08
TR14350-c0_g2	3	31	-3.36923381	2.51171E-07	3.20348E-06
TR19405-c0_g1	3	31	-3.36923381	2.51171E-07	3.20348E-06
TR19751-c0_g1	161	1672	-3.376442254	0	0
TR23997-c0_g1	13	136	-3.387023123	2.62738E-27	1.34141E-25
TR23605-c0_g1	11	116	-3.398549376	1.36422E-23	5.76952E-22
TR4595-c0_g2	27	287	-3.410019425	5.0996E-56	5.85811E-54
TR8018-c0_g2	4	43	-3.426264755	9.65706E-10	1.67363E-08

TR4595-c0_g4	26	280	-3.428843299	6.24345E-55	6.84819E-53
TR4595-c0_g1	22	238	-3.435386145	5.00232E-47	4.49979E-45
TR6467-c0_g3	25	275	-3.459431619	2.36802E-54	2.5643E-52
TR1120-c0_g1	7	77	-3.459431619	2.13262E-16	6.31661E-15
TR17626-c0_g1	4	44	-3.459431619	5.31752E-10	9.427E-09
TR13731-c0_g6	2	22	-3.459431619	1.12982E-05	0.000109825
TR3885-c0_g1	2	22	-3.459431619	1.12982E-05	0.000109825
TR7829-c0_g1	2	22	-3.459431619	1.12982E-05	0.000109825
TR10729-c0_g2	1	11	-3.459431619	0.001904776	0.010490352
TR13731-c0_g4	1	11	-3.459431619	0.001904776	0.010490352
TR19043-c0_g2	1	11	-3.459431619	0.001904776	0.010490352
TR9830-c0_g1	1	11	-3.459431619	0.001904776	0.010490352
TR9830-c0_g2	1	11	-3.459431619	0.001904776	0.010490352
TR9830-c0_g3	1	11	-3.459431619	0.001904776	0.010490352
TR9830-c0_g4	1	11	-3.459431619	0.001904776	0.010490352
TR9830-c0_g5	1	11	-3.459431619	0.001904776	0.010490352
TR10103-c0_g1	23	263	-3.515357033	1.08363E-52	1.10318E-50
TR5319-c0_g1	9	103	-3.516575526	1.16029E-21	4.54526E-20
TR20309-c0_g1	10	116	-3.5360529	2.73933E-24	1.20653E-22
TR12605-c0_g1	23	268	-3.542527234	5.75879E-54	6.08119E-52
TR27294-c0_g1	5	60	-3.584962501	1.96551E-13	4.78058E-12
TR16937-c0_g1	2	24	-3.584962501	3.33199E-06	3.54716E-05
TR25673-c0_g1	2	24	-3.584962501	3.33199E-06	3.54716E-05
TR8287-c2_g4	2	24	-3.584962501	3.33199E-06	3.54716E-05
TR10150-c0_g1	1	12	-3.584962501	0.001010874	0.006116087
TR10150-c0_g2	1	12	-3.584962501	0.001010874	0.006116087
TR10729-c0_g1	1	12	-3.584962501	0.001010874	0.006116087
TR17186-c0_g1	1	12	-3.584962501	0.001010874	0.006116087
TR17985-c0_g2	1	12	-3.584962501	0.001010874	0.006116087
TR1913-c0_g1	1	12	-3.584962501	0.001010874	0.006116087
TR23268-c0_g2	1	12	-3.584962501	0.001010874	0.006116087
TR25354-c0_g1	1	12	-3.584962501	0.001010874	0.006116087
TR2962-c0_g1	1	12	-3.584962501	0.001010874	0.006116087
TR4706-c0_g1	1	12	-3.584962501	0.001010874	0.006116087
TR9449-c0_g1	1	12	-3.584962501	0.001010874	0.006116087
TR13006-c0_g1	3	37	-3.624490865	6.78566E-09	1.05453E-07
TR8018-c0_g1	3	37	-3.624490865	6.78566E-09	1.05453E-07
TR23984-c0_g1	5	62	-3.632268215	5.96158E-14	1.49271E-12
TR11156-c0_g1	23	287	-3.641344971	8.05471E-59	9.57628E-57
TR4688-c2_g1	107	1342	-3.64870197	2.0235E-268	1.3232E-265
TR15355-c0_g1	11	138	-3.649092838	3.18971E-29	1.7722E-27
TR10536-c0_g1	9	113	-3.650253961	3.12779E-24	1.37407E-22
TR73-c0_g1	5	63	-3.655351829	3.28192E-14	8.39052E-13
TR18750-c0_g1	7	89	-3.668378509	1.73361E-19	6.02732E-18

TR16449-c0_g1	11	140	-3.669851398	9.76587E-30	5.55293E-28
TR15695-c0_g1	4	51	-3.672425342	8.06149E-12	1.73489E-10
TR8808-c0_g1	8	103	-3.686500527	2.23909E-22	8.99939E-21
TR15021-c0_g1	12	155	-3.691161905	7.15444E-33	4.55562E-31
TR16013-c0_g1	5	65	-3.700439718	9.9395E-15	2.63627E-13
TR3926-c0_g1	3	39	-3.700439718	2.03031E-09	3.35452E-08
TR3084-c0_g1	2	26	-3.700439718	9.82166E-07	1.14214E-05
TR8076-c0_g1	2	26	-3.700439718	9.82166E-07	1.14214E-05
TR10162-c0_g1	1	13	-3.700439718	0.000537367	0.003528759
TR24040-c0_g1	1	13	-3.700439718	0.000537367	0.003528759
TR13403-c0_g1	8	105	-3.714245518	6.8259E-23	2.80991E-21
TR20325-c0_g1	9	119	-3.724892762	8.90469E-26	4.21703E-24
TR10023-c0_g1	24	320	-3.736965594	1.47986E-66	1.95035E-64
TR18330-c0_g1	9	123	-3.772589504	8.28001E-27	4.11612E-25
TR8751-c0_g1	2	28	-3.807354922	2.89635E-07	3.65566E-06
TR17985-c0_g3	1	14	-3.807354922	0.000286184	0.002027853
TR21031-c0_g1	1	14	-3.807354922	0.000286184	0.002027853
TR21036-c0_g1	1	14	-3.807354922	0.000286184	0.002027853
TR25529-c0_g1	1	14	-3.807354922	0.000286184	0.002027853
TR7086-c0_g1	1	14	-3.807354922	0.000286184	0.002027853
TR9143-c0_g1	1	14	-3.807354922	0.000286184	0.002027853
TR12865-c0_g1	11	155	-3.816692787	1.34193E-33	8.80869E-32
TR3950-c0_g1	8	113	-3.820178962	5.86531E-25	2.65208E-23
TR17943-c0_g1	15	213	-3.827819025	1.30495E-45	1.13773E-43
TR13401-c0_g1	18	258	-3.841302254	5.38984E-55	5.98918E-53
TR10339-c0_g1	3	43	-3.841302254	1.81544E-10	3.36954E-09
TR13006-c0_g2	3	43	-3.841302254	1.81544E-10	3.36954E-09
TR10041-c0_g1	18	261	-3.857980995	9.11784E-56	1.03344E-53
TR14350-c0_g1	2	29	-3.857980995	1.57344E-07	2.06888E-06
TR15026-c0_g1	2	29	-3.857980995	1.57344E-07	2.06888E-06
TR25673-c0_g3	2	29	-3.857980995	1.57344E-07	2.06888E-06
TR10116-c0_g1	27	397	-3.878107695	3.82489E-84	6.5685E-82
TR21696-c0_g1	226	3364	-3.895783028	0	0
TR25421-c0_g4	4	60	-3.906890596	3.63836E-14	9.28782E-13
TR9455-c0_g1	2	30	-3.906890596	8.55078E-08	1.15836E-06
TR24430-c0_g1	1	15	-3.906890596	0.000152712	0.001159067
TR5244-c0_g1	1	15	-3.906890596	0.000152712	0.001159067
TR15590-c0_g1	3	46	-3.938599455	2.96976E-11	6.01069E-10
TR21535-c0_g2	2	31	-3.95419631	4.64883E-08	6.56447E-07
TR683-c9_g1	145	2270	-3.968567492	0	0
TR14788-c0_g1	4	63	-3.977279923	6.01116E-15	1.63778E-13
TR1253-c1_g1	5	80	-4	1.26191E-18	4.17395E-17
TR5358-c0_g2	1	16	-4	8.1658E-05	0.000660778
TR13664-c0_g1	9	145	-4.009984089	1.72233E-32	1.0726E-30

TR662-c0_g1	46	745	-4.017534659	1.2383E-159	4.386E-157
TR7687-c0_g1	7	114	-4.025535092	5.90487E-26	2.80421E-24
TR6743-c0_g5	45	734	-4.027783157	1.5293E-157	5.1999E-155
TR6743-c0_g2	53	872	-4.04026387	3.9953E-187	1.5796E-184
TR13733-c0_g1	13	214	-4.041027268	2.43557E-47	2.20255E-45
TR662-c0_g2	53	883	-4.058349173	5.9501E-190	2.4673E-187
TR6789-c1_g2	16	269	-4.071462363	2.67984E-59	3.20851E-57
TR13240-c0_g1	4	68	-4.087462841	2.99651E-16	8.79874E-15
TR19948-c0_g1	4	68	-4.087462841	2.99651E-16	8.79874E-15
TR11703-c0_g2	2	34	-4.087462841	7.49348E-09	1.16028E-07
TR8751-c0_g2	2	34	-4.087462841	7.49348E-09	1.16028E-07
TR12445-c0_g1	1	17	-4.087462841	4.3757E-05	0.000376672
TR953-c0_g1	8	137	-4.098032083	3.65805E-31	2.19759E-29
TR7798-c0_g1	6	103	-4.101538026	7.54933E-24	3.26587E-22
TR15629-c1_g1	126	2164	-4.10220486	0	0
TR26994-c0_g1	12	209	-4.122396631	8.65506E-47	7.70405E-45
TR10871-c0_g1	4	70	-4.129283017	9.03969E-17	2.72012E-15
TR13730-c1_g1	2	35	-4.129283017	4.08291E-09	6.52396E-08
TR21535-c0_g3	2	36	-4.169925001	2.22606E-09	3.66369E-08
TR22325-c0_g1	1	18	-4.169925001	2.34985E-05	0.000214442
TR10626-c0_g1	0	9	-4.169925001	0.002788147	0.014567361
TR10962-c0_g2	0	9	-4.169925001	0.002788147	0.014567361
TR12712-c0_g1	0	9	-4.169925001	0.002788147	0.014567361
TR13813-c0_g7	0	9	-4.169925001	0.002788147	0.014567361
TR14592-c0_g1	0	9	-4.169925001	0.002788147	0.014567361
TR1467-c0_g2	0	9	-4.169925001	0.002788147	0.014567361
TR16713-c3_g1	0	9	-4.169925001	0.002788147	0.014567361
TR22028-c0_g1	0	9	-4.169925001	0.002788147	0.014567361
TR23698-c6_g2	0	9	-4.169925001	0.002788147	0.014567361
TR24087-c0_g1	0	9	-4.169925001	0.002788147	0.014567361
TR27262-c0_g1	0	9	-4.169925001	0.002788147	0.014567361
TR27339-c0_g1	0	9	-4.169925001	0.002788147	0.014567361
TR4536-c0_g1	0	9	-4.169925001	0.002788147	0.014567361
TR4794-c0_g1	0	9	-4.169925001	0.002788147	0.014567361
TR5666-c0_g1	0	9	-4.169925001	0.002788147	0.014567361
TR6879-c0_g1	0	9	-4.169925001	0.002788147	0.014567361
TR8043-c0_g1	0	9	-4.169925001	0.002788147	0.014567361
TR8199-c0_g2	0	9	-4.169925001	0.002788147	0.014567361
TR9449-c0_g3	0	9	-4.169925001	0.002788147	0.014567361
TR9449-c0_g4	0	9	-4.169925001	0.002788147	0.014567361
TR23278-c0_g1	3	55	-4.196397213	1.31489E-13	3.24455E-12
TR803-c0_g1	11	202	-4.198779864	1.01049E-45	8.85548E-44
TR6789-c1_g1	17	317	-4.220876189	6.43348E-71	9.1914E-69
TR14789-c1_g1	150	2801	-4.222907578	0	0

TR1653-c0_g1	1	19	-4.247927513	1.26469E-05	0.000121272
TR23200-c0_g1	1	19	-4.247927513	1.26469E-05	0.000121272
TR2420-c0_g2	1	19	-4.247927513	1.26469E-05	0.000121272
TR21026-c0_g2	6	117	-4.285402219	1.80305E-27	9.43209E-26
TR2691-c0_g1	353	6949	-4.299065292	0	0
TR12386-c0_g1	1	20	-4.321928095	6.82167E-06	6.88293E-05
TR10631-c0_g1	0	10	-4.321928095	0.001465685	0.008435542
TR1203-c0_g1	0	10	-4.321928095	0.001465685	0.008435542
TR1305-c0_g2	0	10	-4.321928095	0.001465685	0.008435542
TR14001-c0_g1	0	10	-4.321928095	0.001465685	0.008435542
TR14064-c0_g1	0	10	-4.321928095	0.001465685	0.008435542
TR1431-c0_g1	0	10	-4.321928095	0.001465685	0.008435542
TR1467-c0_g4	0	10	-4.321928095	0.001465685	0.008435542
TR17087-c0_g1	0	10	-4.321928095	0.001465685	0.008435542
TR20056-c0_g1	0	10	-4.321928095	0.001465685	0.008435542
TR2014-c0_g1	0	10	-4.321928095	0.001465685	0.008435542
TR20701-c0_g2	0	10	-4.321928095	0.001465685	0.008435542
TR22909-c0_g1	0	10	-4.321928095	0.001465685	0.008435542
TR23183-c0_g1	0	10	-4.321928095	0.001465685	0.008435542
TR23533-c0_g1	0	10	-4.321928095	0.001465685	0.008435542
TR24061-c0_g1	0	10	-4.321928095	0.001465685	0.008435542
TR25378-c0_g1	0	10	-4.321928095	0.001465685	0.008435542
TR25378-c0_g2	0	10	-4.321928095	0.001465685	0.008435542
TR26518-c0_g6	0	10	-4.321928095	0.001465685	0.008435542
TR27768-c0_g1	0	10	-4.321928095	0.001465685	0.008435542
TR9851-c0_g1	0	10	-4.321928095	0.001465685	0.008435542
TR27760-c0_g1	3	61	-4.345774837	3.59999E-15	9.93581E-14
TR9348-c0_g1	3	63	-4.392317423	1.08869E-15	3.06442E-14
TR1653-c0_g2	1	21	-4.392317423	3.68772E-06	3.89175E-05
TR2420-c0_g1	1	21	-4.392317423	3.68772E-06	3.89175E-05
TR26687-c0_g1	1	21	-4.392317423	3.68772E-06	3.89175E-05
TR5943-c0_g1	1	21	-4.392317423	3.68772E-06	3.89175E-05
TR4656-c0_g1	5	109	-4.44625623	3.92643E-26	1.89174E-24
TR11527-c0_g1	0	11	-4.459431619	0.000775714	0.004880888
TR11703-c0_g1	0	11	-4.459431619	0.000775714	0.004880888
TR1467-c0_g1	0	11	-4.459431619	0.000775714	0.004880888
TR1467-c0_g3	0	11	-4.459431619	0.000775714	0.004880888
TR19043-c0_g1	0	11	-4.459431619	0.000775714	0.004880888
TR2449-c0_g1	0	11	-4.459431619	0.000775714	0.004880888
TR25052-c0_g2	0	11	-4.459431619	0.000775714	0.004880888
TR26036-c0_g2	0	11	-4.459431619	0.000775714	0.004880888
TR3169-c0_g1	0	11	-4.459431619	0.000775714	0.004880888
TR5461-c0_g1	0	11	-4.459431619	0.000775714	0.004880888
TR6229-c0_g1	0	11	-4.459431619	0.000775714	0.004880888

TR3947-c0_g1	2	45	-4.491853096	9.79394E-12	2.07878E-10
TR19780-c0_g1	3	68	-4.502500341	5.51971E-17	1.67277E-15
TR7352-c0_g1	5	114	-4.510961919	2.03011E-27	1.05228E-25
TR16713-c1_g1	3	69	-4.523561956	3.04471E-17	9.446E-16
TR13813-c0_g4	1	23	-4.523561956	1.08484E-06	1.25383E-05
TR23698-c6_g1	12	282	-4.554588852	1.55298E-65	2.01548E-63
TR10333-c1_g1	0	12	-4.584962501	0.000413158	0.002819846
TR1158-c0_g1	0	12	-4.584962501	0.000413158	0.002819846
TR11670-c0_g1	0	12	-4.584962501	0.000413158	0.002819846
TR11670-c0_g2	0	12	-4.584962501	0.000413158	0.002819846
TR14720-c0_g1	0	12	-4.584962501	0.000413158	0.002819846
TR17594-c0_g1	0	12	-4.584962501	0.000413158	0.002819846
TR18099-c0_g3	0	12	-4.584962501	0.000413158	0.002819846
TR18949-c0_g1	0	12	-4.584962501	0.000413158	0.002819846
TR21535-c0_g1	0	12	-4.584962501	0.000413158	0.002819846
TR23548-c0_g1	0	12	-4.584962501	0.000413158	0.002819846
TR25075-c0_g2	0	12	-4.584962501	0.000413158	0.002819846
TR21026-c0_g1	4	97	-4.599912842	9.44527E-24	4.06537E-22
TR11674-c0_g2	1	25	-4.64385619	3.21937E-07	4.05735E-06
TR10374-c2_g6	1	26	-4.700439718	1.75945E-07	2.28867E-06
TR15442-c0_g1	1	26	-4.700439718	1.75945E-07	2.28867E-06
TR15742-c0_g1	1	26	-4.700439718	1.75945E-07	2.28867E-06
TR11527-c0_g2	0	13	-4.700439718	0.000221374	0.001622963
TR13813-c0_g2	0	13	-4.700439718	0.000221374	0.001622963
TR17257-c0_g2	0	13	-4.700439718	0.000221374	0.001622963
TR20701-c0_g1	0	13	-4.700439718	0.000221374	0.001622963
TR25075-c0_g1	0	13	-4.700439718	0.000221374	0.001622963
TR26518-c0_g2	0	13	-4.700439718	0.000221374	0.001622963
TR26556-c0_g1	0	13	-4.700439718	0.000221374	0.001622963
TR4028-c0_g1	0	13	-4.700439718	0.000221374	0.001622963
TR5605-c0_g1	0	13	-4.700439718	0.000221374	0.001622963
TR5605-c0_g2	0	13	-4.700439718	0.000221374	0.001622963
TR6174-c0_g1	0	13	-4.700439718	0.000221374	0.001622963
TR10085-c1_g2	8	209	-4.707359132	1.20652E-49	1.17214E-47
TR1933-c0_g1	7	184	-4.716207034	6.0305E-44	5.02581E-42
TR11881-c0_g2	36	965	-4.744460131	5.3702E-223	2.5361E-220
TR15004-c0_g1	2	55	-4.781359714	2.54901E-14	6.58609E-13
TR12121-c0_g2	8	222	-4.794415866	6.04988E-53	6.23369E-51
TR17475-c0_g1	2	56	-4.807354922	1.41242E-14	3.70293E-13
TR11583-c0_g1	0	14	-4.807354922	0.000119286	0.000930715
TR16645-c0_g1	0	14	-4.807354922	0.000119286	0.000930715
TR19511-c0_g1	0	14	-4.807354922	0.000119286	0.000930715
TR21514-c1_g1	0	14	-4.807354922	0.000119286	0.000930715
TR9333-c0_g1	0	14	-4.807354922	0.000119286	0.000930715

TR17196-c1_g1	4	113	-4.820178962	7.75564E-28	4.13342E-26
TR9254-c0_g1	2	57	-4.832890014	7.83309E-15	2.10717E-13
TR13436-c0_g1	0	15	-4.906890596	6.46226E-05	0.000534632
TR13803-c1_g1	0	15	-4.906890596	6.46226E-05	0.000534632
TR17336-c0_g1	0	15	-4.906890596	6.46226E-05	0.000534632
TR7065-c0_g2	0	15	-4.906890596	6.46226E-05	0.000534632
TR8490-c0_g1	6	181	-4.914883386	8.14114E-44	6.75171E-42
TR23698-c6_g3	5	151	-4.916476644	7.63023E-37	5.52017E-35
TR3169-c0_g3	2	61	-4.930737338	7.47405E-16	2.13561E-14
TR11224-c19_g1	36	1108	-4.943817165	5.0007E-259	3.0364E-256
TR27757-c0_g1	4	126	-4.977279923	3.99506E-31	2.3916E-29
TR8601-c0_g2	12	382	-4.992466327	7.77679E-91	1.51972E-88
TR8601-c0_g1	15	479	-4.99699125	1.94E-113	4.3976E-111
TR16467-c0_g1	4	128	-5	1.25373E-31	7.61254E-30
TR1180-c0_g1	1	32	-5	4.89763E-09	7.7385E-08
TR13142-c0_g1	1	32	-5	4.89763E-09	7.7385E-08
TR11644-c0_g2	0	16	-5	3.51878E-05	0.000312397
TR14284-c0_g1	0	16	-5	3.51878E-05	0.000312397
TR15608-c0_g1	0	16	-5	3.51878E-05	0.000312397
TR25290-c0_g1	0	16	-5	3.51878E-05	0.000312397
TR594-c0_g1	5	164	-5.03562391	4.10606E-40	3.1026E-38
TR3471-c1_g1	5	165	-5.044394119	2.30615E-40	1.76655E-38
TR7480-c0_g1	3	99	-5.044394119	6.88331E-25	3.10412E-23
TR24887-c0_g1	2	66	-5.044394119	4.04077E-17	1.24454E-15
TR11644-c0_g1	0	17	-5.087462841	1.92537E-05	0.000178873
TR13790-c0_g4	0	17	-5.087462841	1.92537E-05	0.000178873
TR15754-c0_g2	0	17	-5.087462841	1.92537E-05	0.000178873
TR18371-c0_g1	0	17	-5.087462841	1.92537E-05	0.000178873
TR22136-c0_g1	0	17	-5.087462841	1.92537E-05	0.000178873
TR26615-c0_g1	0	17	-5.087462841	1.92537E-05	0.000178873
TR2997-c0_g1	0	17	-5.087462841	1.92537E-05	0.000178873
TR7065-c0_g1	0	17	-5.087462841	1.92537E-05	0.000178873
TR11303-c0_g1	6	207	-5.108524457	2.47129E-50	2.42862E-48
TR6466-c0_g1	2	70	-5.129283017	3.97566E-18	1.28257E-16
TR10289-c1_g2	1	36	-5.169925001	4.6804E-10	8.41152E-09
TR11550-c0_g1	0	18	-5.169925001	1.05841E-05	0.000103297
TR18875-c0_g1	0	18	-5.169925001	1.05841E-05	0.000103297
TR20155-c0_g1	0	18	-5.169925001	1.05841E-05	0.000103297
TR25324-c0_g2	0	18	-5.169925001	1.05841E-05	0.000103297
TR26518-c0_g3	0	18	-5.169925001	1.05841E-05	0.000103297
TR4395-c0_g1	0	18	-5.169925001	1.05841E-05	0.000103297
TR4657-c0_g1	0	18	-5.169925001	1.05841E-05	0.000103297
TR11881-c0_g3	1	37	-5.209453366	2.61458E-10	4.80554E-09
TR26427-c0_g1	0	19	-5.247927513	5.84421E-06	5.95322E-05

TR17194-c0_g1	2	79	-5.303780748	2.26219E-20	8.28305E-19
TR25183-c0_g1	5	200	-5.321928095	4.75713E-49	4.54368E-47
TR15528-c0_g1	1	40	-5.321928095	4.60764E-11	9.07717E-10
TR17091-c0_g1	1	40	-5.321928095	4.60764E-11	9.07717E-10
TR5212-c0_g1	1	40	-5.321928095	4.60764E-11	9.07717E-10
TR15742-c0_g2	0	20	-5.321928095	3.24081E-06	3.4631E-05
TR15754-c0_g1	0	20	-5.321928095	3.24081E-06	3.4631E-05
TR26604-c0_g1	0	20	-5.321928095	3.24081E-06	3.4631E-05
TR15229-c0_g3	4	167	-5.383704292	2.61584E-41	2.08792E-39
TR11674-c0_g1	0	21	-5.392317423	1.80452E-06	2.00256E-05
TR13790-c0_g6	0	21	-5.392317423	1.80452E-06	2.00256E-05
TR1986-c0_g1	0	21	-5.392317423	1.80452E-06	2.00256E-05
TR25321-c1_g1	0	21	-5.392317423	1.80452E-06	2.00256E-05
TR25324-c0_g1	0	21	-5.392317423	1.80452E-06	2.00256E-05
TR23361-c0_g3	5	218	-5.44625623	1.87195E-53	1.94058E-51
TR13813-c0_g8	0	22	-5.459431619	1.00876E-06	1.16827E-05
TR16745-c0_g4	0	22	-5.459431619	1.00876E-06	1.16827E-05
TR17848-c0_g2	0	22	-5.459431619	1.00876E-06	1.16827E-05
TR25319-c0_g2	0	22	-5.459431619	1.00876E-06	1.16827E-05
TR6538-c0_g3	0	22	-5.459431619	1.00876E-06	1.16827E-05
TR7787-c0_g1	5	221	-5.465974465	3.48408E-54	3.7254E-52
TR5496-c0_g2	0	23	-5.523561956	5.6606E-07	6.87412E-06
TR21349-c0_g1	6	277	-5.528779665	1.61294E-67	2.15922E-65
TR8538-c0_g1	12	557	-5.536571017	7.4332E-134	1.9442E-131
TR13472-c0_g1	0	24	-5.584962501	3.18811E-07	4.02093E-06
TR18780-c0_g1	1	50	-5.64385619	1.57861E-13	3.86721E-12
TR25108-c0_g2	0	25	-5.64385619	1.80196E-07	2.34038E-06
TR25321-c0_g1	0	25	-5.64385619	1.80196E-07	2.34038E-06
TR6675-c0_g1	1	51	-5.672425342	9.02709E-14	2.25695E-12
TR24738-c0_g1	0	26	-5.700439718	1.02198E-07	1.37136E-06
TR16525-c0_g1	2	107	-5.741466986	3.49837E-27	1.78075E-25
TR23646-c0_g1	3	162	-5.754887502	2.74736E-40	2.08521E-38
TR23698-c6_g4	0	27	-5.754887502	5.81548E-08	8.0909E-07
TR25108-c0_g1	0	27	-5.754887502	5.81548E-08	8.0909E-07
TR13194-c0_g1	0	28	-5.807354922	3.31989E-08	4.79546E-07
TR9802-c0_g1	0	28	-5.807354922	3.31989E-08	4.79546E-07
TR11881-c0_g1	60	3436	-5.839623726	0	0
TR4168-c0_g1	5	297	-5.892391026	2.51068E-72	3.67973E-70
TR1397-c0_g1	0	30	-5.906890596	1.09201E-08	1.65912E-07
TR10354-c0_g1	0	31	-5.95419631	6.2909E-09	9.82126E-08
TR13730-c1_g3	0	31	-5.95419631	6.2909E-09	9.82126E-08
TR13803-c0_g1	0	31	-5.95419631	6.2909E-09	9.82126E-08
TR495-c0_g1	5	310	-5.95419631	2.29657E-75	3.54952E-73
TR18934-c0_g1	1	64	-6	7.18654E-17	2.16633E-15

TR21409-c0_g2	0	33	-6.044394119	2.10561E-09	3.47218E-08
TR22981-c1_g1	8	531	-6.052568051	1.5121E-127	3.7806E-125
TR24283-c3_g1	8	537	-6.068778278	6.2663E-129	1.5901E-126
TR26441-c0_g1	0	34	-6.087462841	1.22318E-09	2.0775E-08
TR5496-c0_g1	0	34	-6.087462841	1.22318E-09	2.0775E-08
TR14553-c0_g1	0	35	-6.129283017	7.12445E-10	1.24614E-08
TR25319-c0_g1	0	35	-6.129283017	7.12445E-10	1.24614E-08
TR21464-c0_g2	0	38	-6.247927513	1.42931E-10	2.67622E-09
TR27361-c0_g3	0	38	-6.247927513	1.42931E-10	2.67622E-09
TR8835-c0_g1	1	79	-6.303780748	2.49205E-20	9.03372E-19
TR22984-c0_g1	2	164	-6.357552005	2.30673E-40	1.76655E-38
TR16713-c0_g1	0	42	-6.392317423	1.73539E-11	3.57624E-10
TR16623-c0_g2	0	43	-6.426264755	1.03013E-11	2.1729E-10
TR10357-c1_g4	0	44	-6.459431619	6.12792E-12	1.33056E-10
TR14433-c0_g1	0	45	-6.491853096	3.65295E-12	8.08659E-11
TR25264-c0_g1	1	91	-6.50779464	5.07611E-23	2.11004E-21
TR16595-c0_g1	0	50	-6.64385619	2.8334E-13	6.80388E-12
TR16745-c0_g3	0	50	-6.64385619	2.8334E-13	6.80388E-12
TR6538-c0_g2	0	52	-6.700439718	1.03261E-13	2.57038E-12
TR27448-c0_g1	1	109	-6.768184325	6.01973E-27	3.0101E-25
TR12283-c0_g2	0	56	-6.807354922	1.40094E-14	3.68698E-13
TR2816-c0_g1	1	113	-6.820178962	8.38326E-28	4.45395E-26
TR1713-c0_g1	0	57	-6.832890014	8.53859E-15	2.27535E-13
TR21409-c0_g1	1	120	-6.906890596	2.7432E-29	1.52911E-27
TR12283-c0_g1	1	138	-7.108524457	4.90445E-33	3.17042E-31
TR3477-c4_g1	12	1726	-7.168254248	0	0
TR13803-c1_g2	0	75	-7.22881869	1.48769E-18	4.88276E-17
TR947-c4_g1	11	1736	-7.302119614	0	0
TR20214-c0_g4	0	80	-7.321928095	1.45145E-19	5.07748E-18
TR26637-c0_g1	0	84	-7.392317423	2.304E-20	8.40076E-19
TR15034-c0_g1	0	85	-7.409390936	1.45843E-20	5.36692E-19
TR4443-c0_g1	0	88	-7.459431619	3.72354E-21	1.42579E-19
TR2802-c0_g1	0	91	-7.50779464	9.59866E-22	3.76882E-20
TR20214-c0_g2	0	93	-7.539158811	3.90812E-22	1.55605E-20
TR15257-c1_g1	0	106	-7.727920455	1.24911E-24	5.55928E-23
TR14255-c0_g1	0	121	-7.918863237	1.98461E-27	1.03183E-25
TR17031-c0_g1	0	166	-8.375039431	2.09081E-35	1.46886E-33
TR26865-c0_g1	0	196	-8.614709844	1.91747E-40	1.4818E-38
TR8190-c0_g1	0	225	-8.813781191	3.84363E-45	3.3171E-43
TR8214-c0_g1	0	227	-8.826548487	1.84632E-45	1.60153E-43
TR23704-c0_g1	0	230	-8.845490051	6.16489E-46	5.45892E-44
TR10313-c3_g1	0	2094	-12.03204573	1.7188E-247	9.7406E-245
TR10348-c1_g1	0	3349	-12.70951466	0	0
